# The Role of lncRNA AF117829.1 in the Immunological Pathogenesis of Severe Aplastic Anaemia

**DOI:** 10.1155/2021/5587921

**Published:** 2021-03-16

**Authors:** Yang Li, Ling Deng, Xiaofeng Pan, Chunyan Liu, Rong Fu

**Affiliations:** Department of Haematology, Tianjin Medical University General Hospital, Tianjin 300052, China

## Abstract

**Objective:**

Severe aplastic anaemia (SAA) is an autoimmune disease with immune tolerance dysfunction mediated by hyperactivated T lymphocytes that target the haematopoietic system. Numerous studies suggest that long noncoding RNAs (lncRNAs) play a significant role in almost every level of gene function/regulation. However, their specific mechanisms in SAA remain undetermined. This study is aimed at determining the role of key lncRNAs in CD8+ T lymphocytes in the mechanisms of SAA.

**Methods:**

RNA-seq was performed to detect all lncRNAs and mRNAs in peripheral CD8+ T lymphocytes from SAA patients and healthy controls. The lncRNA targets were predicted by bioinformatics, Gene Ontology (GO) analysis, and Kyoto Encyclopedia of Genes and Genomes (KEGG) analysis. RT-qPCR was used to verify the expression of key lncRNAs and their predicted targets. We screened lncRNA AF117829.1, which was correlated with autoimmune diseases and downregulated in CD8+ T lymphocytes, and further validated its effects on CD8+ T lymphocytes from SAA patients.

**Results:**

We systematically described the lncRNA/mRNA expression changes in CD8+ T lymphocytes in SAA patients and assessed their possible biological functions and signalling pathways. A total of 194 lncRNAs and 2099 mRNAs were changed in SAA patients versus healthy controls. These differentially expressed lncRNAs/mRNAs were associated with organelle components, catalytic activity, the response to stimulation, signal transduction, the immune system and metabolic processes. The downregulated expression of one altered factor, lncRNA AF117829.1, in CD8+ T lymphocytes from SAA patients increased CD8+ T lymphocyte immune function by promoting RIP2 expression. lncRNA AF117829.1 overexpression in CD8+ T lymphocytes reduced perforin and granzyme B expression. The same effect was achieved with GSK583, a RIP2 kinase inhibitor.

**Conclusions:**

The proliferation and overactivation of CD8+ T lymphocytes, also known as cytotoxic T cells (CTLs), directly induce bone marrow (BM) failure in SAA patients, but the specific mechanism remains unclear. We found that lncRNA AF117829.1 and its target genes were associated with T cell proliferation, differentiation, and immune dysregulation and that lncRNA AF117829.1 regulated CD8+ T lymphocyte function in SAA patients by promoting RIP2 expression. These findings improve our understanding of the molecular mechanism of immune pathogenesis and provide potential targets for SAA diagnosis and treatment.

## 1. Introduction

Severe aplastic anaemia (SAA) refers to a haematological disease represented by pancytopenia in association with bone marrow (BM) hypoplasia/aplasia. The typical clinical symptoms of SAA comprise severe anaemia, infection, and bleeding/bruising; SAA is an acute condition that progresses rapidly [1]. If effective treatment is not given in time, patients often die from severe bleeding or infection. At present, immunosuppressive therapy (IST) and haematopoietic therapy based on antithymocyte globulin, antilymphocyte globulin (ATG/ALG), and cyclosporine A (CsA) have been widely used in the treatment of this disease with an effective rate of up to more than 70%, but these approaches are ineffective in nearly 30% of SAA patients, some of which die from the disease [2]. Even after treatment, 10% of patients in remission may relapse [2]. Therefore, further exploration of the immune pathogenesis of SAA is of great significance for determining new treatment directions and establishing effective diagnostic and prognostic indicators.

Current research has revealed that environmental factors (cytotoxic chemicals, drugs, viral infection, or ionizing radiation) facilitate SAA development by inducing autoimmune damage (mainly mediated by CD8+ T lymphocytes) to haematopoietic stem cells or by inducing other immune responses; the features of these immune cells have aroused great attention from scholars [3]. CD8+ T lymphocytes exert their cytotoxic function on target cells mainly through perforin and granzyme B and the Fas-FasL interaction [4]. We found that the number of activated inhibitory T lymphocytes (mostly CD8+ T lymphocytes) increased and that the cells were in a hyperactive state in SAA patients [5–7]. CD8+ T lymphocytes isolated from SAA patients can cause meaningful damage to BM cells in vitro [8]. Therefore, CD8+ T lymphocytes play a critical role in the pathogenic mechanism of SAA.

Long noncoding RNAs (lncRNAs) were first proposed by Brannan et al. in the 1990s [9]. Studies on lncRNAs are increasing due to state-of-the-art high-throughput sequencing techniques. lncRNAs are greater than 200 nt in length and do not have obvious protein-coding functions [10–12]. In recent decades, increasing evidence has shown that lncRNAs have crucial regulatory effects on mammalian biology [13]. The regulatory role of lncRNAs in autoimmune diseases has attracted widespread attention. The persistent expression of lncRNA-NEAT1 might be a potential cause of the increased production of multiple cytokines in patients with systemic lupus erythaematosus (SLE) [14]. The lipopolysaccharide- (LPS-) induced expression of chemokines and cytokines, including IL-6 and CXCL10, was significantly attenuated as a result of NEAT1 silencing, and lncRNA-NEAT1 might be a target in SLE treatment. Li et al. analysed the lncRNA and mRNA expression profiles of peripheral blood mononuclear cells from immune thrombocytopenia (ITP) patients by gene chip technology [15]. They found that 1177 lncRNAs and 632 mRNAs were significantly downregulated in newly diagnosed ITP patients compared to healthy controls. Furthermore, CD4+ T lymphocytes from ITP patients exhibited increased expression of lncRNA maternally expressed gene 3 (MEG3) [16]. Overexpression of lncRNA MEG3 inhibited miR-125A-5P transcription in CD4+ T lymphocytes under the action of dexamethasone. In vitro experiments showed that downregulation of MEG3 or overexpression of miR-125A-5P could facilitate Foxp3 transcription, inhibit ROR*γ*t expression, and lead to Treg/Th17 imbalance, which provided new ideas for elucidating the molecular mechanism of ITP. There are thousands of lncRNAs in the mammalian genome that regulate gene expression in immunocytes, such as T lymphocytes and dendritic cells (DC), during various immune processes [17, 18]. lncRNA CD244 mediates the methylation of H3K27 at the IFN-*γ*/TNF-*α* gene site and then inhibits IFN-*γ*/TNF-*α* secretion from CD8+ T lymphocytes [19]. Mao et al. found that IL-36*β* enhances the activity of CD8+ T lymphocytes in killing tumour cells by regulating lncRNA-GM16343 [20].

In summary, lncRNAs play an essential role in the development and activation of immune cells. Nevertheless, the expression of lncRNAs in CD8+ T lymphocytes and the mechanism by which they influence the biological activity of CD8+ T lymphocytes from SAA patients are still unknown. To further address these gaps in knowledge, we assessed lncRNA expression and the function of CD8+ T lymphocytes in SAA to provide new insights for the effective diagnosis and management of SAA.

## 2. Materials and Methods

### 2.1. Study Populations

The study enrolled patients and control subjects, each with a signed informed consent form, and was approved by the Ethical Committee of Tianjin Medical University General Hospital. From September 2017 to January 2020, we enrolled 48 newly diagnosed SAA patients who had never received IST; the median age was 44.5 (24-59) years old. We also enrolled 33 patients in remission after IST (R-SAA) and 38 healthy volunteers to serve as controls. The diagnosis of SAA was made according to standard criteria [21].

### 2.2. CD8+ T Lymphocyte Isolation, Enrichment Analysis, and Functional Test

We isolated CD8+ T lymphocytes from the peripheral blood of newly diagnosed SAA patients and healthy controls using CD8 MicroBeads (Miltenyi Biotec, Germany) in accordance with the manufacturer's protocol. To assess CD8+ T lymphocyte enrichment, the cells were stained with antibodies against CD3-APC and CD8-FITC and analysed with the Beckman Coulter CytoFLEX™ Flow Cytometer (CytoFLEX) according to a standard procedure. Perforin and granzyme B expression and cell apoptosis were also analysed with CytoFLEX according to standard procedures.

### 2.3. RNA-seq

RNA-seq was performed as described by Springer Nature Experiments (http://experiments.springernature.com/articles/10.1007/978-1-4939-6539-7_10) [22–24]. After that, Gene Ontology (GO) analysis and Kyoto Encyclopedia of Genes and Genomes (KEGG) enrichment analysis were applied to predict the function of lncRNAs and perform gene annotation [25, 26]. Bedtools (v2.17.0) and Blast (v2.2.28+) software were used to predict whether specific lncRNAs regulate gene expression in cis or in trans, respectively.

### 2.4. RNA Extraction and RT-qPCR

After total RNA was extracted from CD8+ T lymphocytes with TRIzol, the samples were subjected to subsequent reverse transcription to synthesize complementary DNA using the FastQuant RT Kit (with gDNase) according to the manufacturer's instructions. Then, we performed RT-qPCR on a Bio-Rad iQ5 Real-Time System to detect the relative expression of lncRNA AF117829.1, TCONS_00043638, TCONS_00329529, TCONS_00002554, RIP2, ADAM8, DUOX2, and BCR with the primers shown in Table 1 (normalized to the expression of *β*-actin). The data were analysed by using a modification of the 2−*ΔΔ*Ct method.

### 2.5. Western Blotting

Western blotting was carried out as described by Abcam (http://www.abcam.com/ps/pdf/protocols/WB-beginner.pdf). We incubated the blots overnight at 4°C with rabbit anti-GAPDH, rabbit anti-RIP2, rabbit anti-MAPK, and rabbit NF-*κ*B primary antibodies from Cell Signalling Technology to probe the target proteins. Band density was quantified by ImageJ and normalized to that of GAPDH.

### 2.6. Lentivirus Transduction

Lentiviruses expressing lncRNA AF117829.1 were constructed by GeneChem (Shanghai, China). Empty vectors were also purchased from GeneChem. After CD8+ T lymphocyte activation with the T Cell Activation/Expansion Kit (Miltenyi Biotec, Germany), cell transfections of lentivirus expressing lncRNA AF117829.1 or negative control lentivirus were performed in strict accordance with the manufacturer's instructions at a multiplicity of infection (MOI) = 100. Puromycin dihydrochloride (Thermo Fisher) was used as the antibiotic selection agent. The infection rate and subsequent perforin and granzyme B expression were analysed by flow cytometry (FCM) as described above.

### 2.7. Treatment of CD8+ T Lymphocytes with a RIP2 Kinase Inhibitor (GSK583)

After cell magnetic separation and purification, peripheral blood CD8+ T lymphocytes from SAA patients and healthy controls were treated with GSK583 (80 nM) or an equivalent volume of DMSO for 24 h [27]. Apoptosis and perforin and granzyme B expression were detected by FCM as previously described.

### 2.8. Enzyme-Linked Immunosorbent Assay (ELISA)

After culturing the cells for 24 h, the cell supernatants were obtained by centrifugation, and the changes in IL-6 and IL-1*β* were detected by using IL-6 and IL-1*β* ELISA kits (Nanjing SenBeiJia Biological Technology Co., Ltd.).

### 2.9. Statistical Analysis

Normally distributed data were presented as the mean ± standard error, and nonnormally distributed data were presented as the median (25%, 75%). Student's *t*-test (two-tailed), chi-squared *t*-test, one-way ANOVA, and Mann-Whitney *U* test were used to analyse the experimental data. Data analysis was performed with GraphPad Prism (version 7.0) and SPSS 24.0. *p* < 0.05 was deemed to indicate significance.

## 3. Results

### 3.1. Analysis of the Expression Profiles of lncRNAs and mRNAs in Peripheral CD8+ T Lymphocytes from Patients with SAA

**Figure 1:**
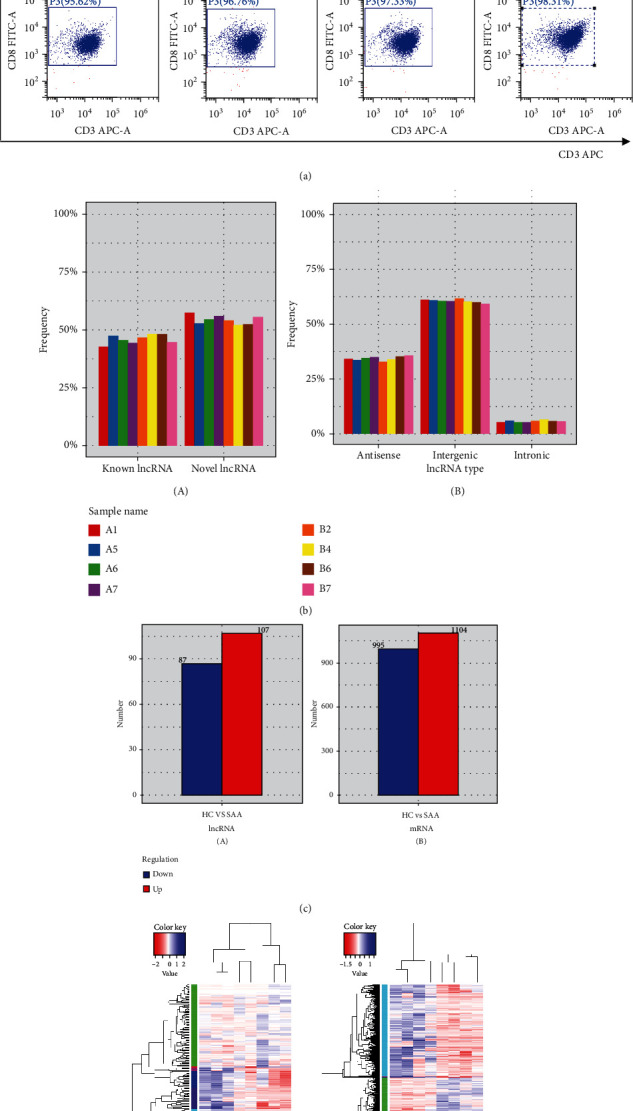
Analysis of the lncRNA and mRNA expression profiles of CD8+ T lymphocytes from patients with SAA. (a) Magnetic-activated cell sorting (MACS) was used to sort CD8+ T lymphocytes from the peripheral mononuclear cells of 4 newly diagnosed SAA patients and 4 healthy controls, and the enrichment of CD8+ T lymphocytes (>90%) was detected by FCM. (b) Description of lncRNAs and related statistics. When annotating known lncRNAs, we first integrated the lncRNA information of the Ensembl database, GENCODE database, University of California, Santa Cruz (UCSC) database, and other authoritative databases. We also used CuffCompare software for annotation (A). Based on the information regarding the position of lncRNAs on the reference genome, the obtained lncRNAs were divided into the following three categories: intergenic lncRNAs, antisense lncRNAs, and intronic lncRNAs, and the number of each type of lncRNA was statistically analysed (B). (c) Screening of differentially expressed target genes of lncRNAs and mRNAs. The test results were screened according to the significance criteria for differences (gene expression change > 2‐fold and FDR ≤ 0.05), and we assessed whether the significant differentially expressed target genes of lncRNAs (A) and mRNAs (B) were up- or downregulated. Blue represents downregulation of the target gene, whereas red represents upregulation of the target gene. There were 194 lncRNAs (107 upregulated and 87 downregulated) and 2099 mRNAs (1104 upregulated and 995 downregulated) with significant expression changes. (d) Hierarchical clustering analysis based on the lncRNA (A) and mRNA (B) expression profiles. Cluster analysis is used to determine the similarity of the data and classify the data according to the similarity. This method was used to cluster closely related genes or genes with the same function into groups, to identify the function of unknown genes or the unknown function of known genes, and to infer whether the genes are involved in the same metabolic process or cell pathway. Different coloured regions represent different clusters and groupings, and the gene expression patterns within each group are similar, indicating that genes within each group may have similar functions or participate in the same biological processes. B2, B4, B6, and B7 represented 4 SAA patients. A1, A5, A6, and A7 represented 4 healthy controls. The blue column indicates highly expressed genes, and the red column indicates genes with lower expression; the log10 (fragments per kilobase of transcript per million mapped reads (FPKM) + 1) value was used for clustering. The colour sequence from red to blue represents an increase in gene expression.

**Figure 2:**
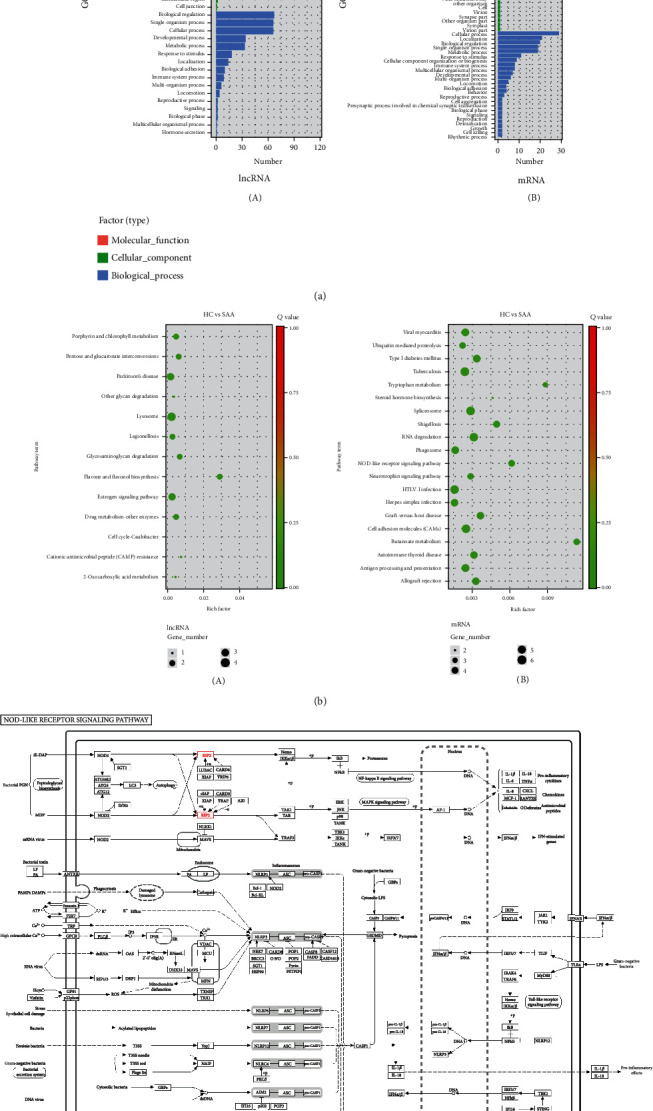
GO and KEGG analysis of the lncRNA and mRNA expression profiles of CD8+ T lymphocytes from patients with SAA. (a) GO analysis of differentially expressed lncRNAs (A) and mRNAs (B). (b) KEGG enrichment bubble plot for differentially expressed lncRNAs (A) and mRNAs (B). (c) The pathway related to our study from KEGG analysis. In this pathway map, RIP2 was upregulated, which is represented in red. The MAPK signalling pathway is closely related to various biological functions, such as T cell differentiation and proliferation. After activation of the NOD1/NOD2 signalling pathway by the NOD-like receptor (NLR) family, the NOD-RIP2 interaction leads to the activation of nuclear factor-*κ*B (NF-*κ*B) and the MAPK pathway and promotes the transcription of proinflammatory cytokines (IL-6, IL-1*β*).

The expression profiles of lncRNAs and mRNAs in CD8+ T lymphocytes were analysed by RNA-seq technology in 4 newly diagnosed SAA patients and 4 healthy controls. The basic characteristics of the populations are shown in Table 2. A total of 2099 altered mRNAs (1104 upregulated mRNAs and 995 downregulated mRNAs) were observed (Figure 1) (Supplementary file S1). GO analysis indicated that the differentially expressed mRNAs were enriched in catalysis, transduction activity, molecular function regulation, cell metabolism, immune system, and other functions. KEGG analysis showed that the differentially expressed mRNAs were mainly enriched in the c-Jun N-terminal kinase (JNK), RAS, nucleotide-binding oligomerization domain 2/receptor-interacting protein 2 (NOD2/RIP2), and mitogen-activated protein kinase (MAPK) signalling pathways. A total of 194 lncRNAs, including 107 upregulated and 87 downregulated lncRNAs, were altered in SAA patients versus healthy controls (Figure 1) (Supplementary file S2). GO analysis showed that the differentially expressed lncRNAs were associated with organelle components, catalytic activity, the response to stimulation, signal transduction inside and outside of cells, biological regulation, and metabolic processes. KEGG analysis revealed that the differentially expressed lncRNAs were involved in ubiquitin-mediated proteomics, antigen processing and presentation, NOD, and other signalling pathways (Figure 2).

According to the microarray data, the lncRNAs related to immune regulation included TCONS_00379951 (lncRNA AF117829.1), TCONS_00043638, TCONS_00329529, and TCONS_00002554. The predicted target gene transcripts related to the above lncRNAs included ENST00000540020, ENST00000415217, ENST00000606851, and ENST00000487968 (Table 3).

## 4. The Expression of lncRNA AF117829.1 in the CD8+ T Lymphocytes of SAA Patients

### 4.1. lncRNA and mRNA Expression in CD8+ T Lymphocytes

**Figure 3:**
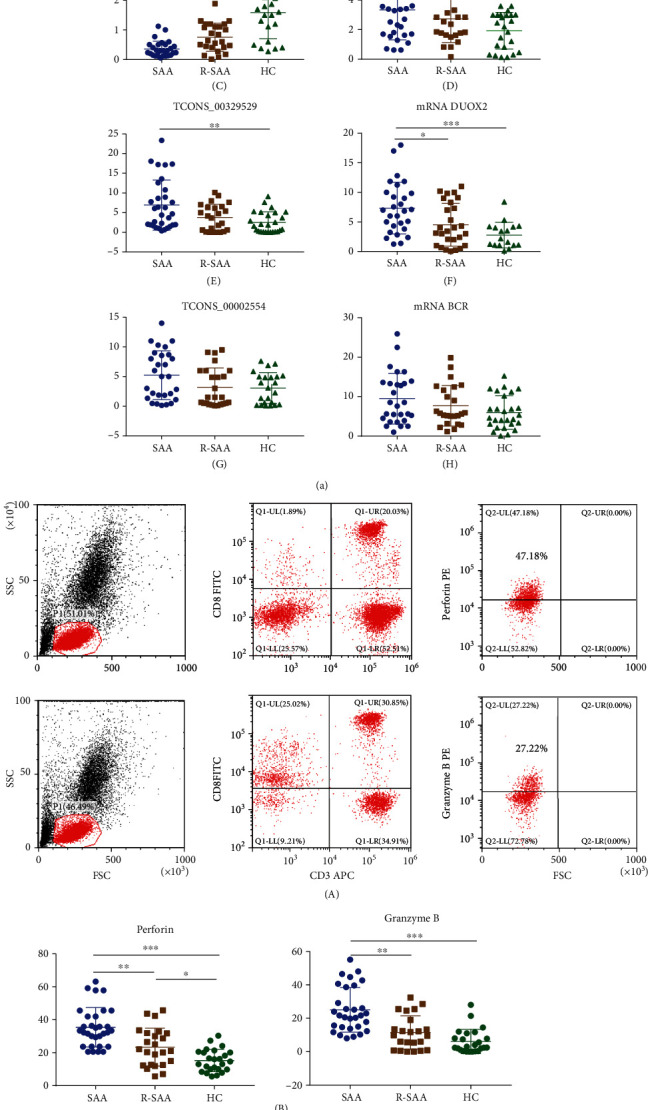
The expression of lncRNA AF117829.1 in the CD8+ T lymphocytes of patients with SAA. (a) lncRNA and mRNA expression in CD8+ T lymphocytes. (b) Perforin and granzyme B expression in CD8+ T lymphocytes detected by FCM (A), and those expressions were upregulated in the CD8+ T lymphocytes of newly diagnosed SAA patients (B). (c) Expression of relevant proteins involved in the RIP2 signalling pathway.

We enrolled 48 newly diagnosed SAA patients, 33 R-SAA patients, and 38 healthy volunteers. The baseline characteristics are shown in Table 4. Among all subjects, differential lncRNA and mRNA expression was considered to be meaningful if the following conditions (gene expression change > 2‐fold and FDR ≤ 0.05) were met. As shown in Table 5, we found that lncRNA AF117829.1 expression was notably decreased in the SAA and R-SAA groups compared with the healthy control group (*p* = 0.0109; *p* = 0.00005). The mRNA expression of its target gene, RIP2, was increased in the SAA and R-SAA groups compared with the healthy control group (*p* = 0.9897; *p* = 0.0003) (Figure 3(a), A and B). lncRNA TCONS_00043638 (*p* = 0.00006; *p* = 0.0174) and its target gene mRNA ADAM8 (*p* = 0.9532; *p* = 0.0362) had the same expression tendency as lncRNA AF117829.1 (Figure 3(a), C and D). The expression of lncRNA TCONS_00329529 (*p* = 0.1423; *p* = 0.0048) and its target gene mRNA DUOX2 was increased in both the SAA and R-SAA groups compared with the healthy control group (*p* = 0.0256; *p* = 0.0008) (Figure 3(a), E and F). The expression of lncRNA TCONS_00002554 and its target gene mRNA BCR was not significantly different between the groups (Figure 3(a), G and H). The above results were basically consistent with the sequencing results.

### 4.2. Perforin and Granzyme B Expression Was Increased in CD8+ T Lymphocytes from SAA Patients

The expression of both perforin and granzyme B was upregulated in the CD8+ T lymphocytes of newly diagnosed SAA patients (33.34% (23.7%, 41.95%); 21.68% (14.45%, 38.9%)) compared with those of R-SAA patients (21.55% (12.57%, 31.85%); 9.8% (0.9%, 19%)) and healthy controls (14.6% (9.91%, 20.33%); 2.75% (0.517%, 10.25%)) (*p* < 0.05) (Figure 3(b)).

### 4.3. The Correlation between the Expression of the Previously Mentioned lncRNAs and the Expression of Perforin and Granzyme B in CD8+ T Lymphocytes

**Figure 4:**
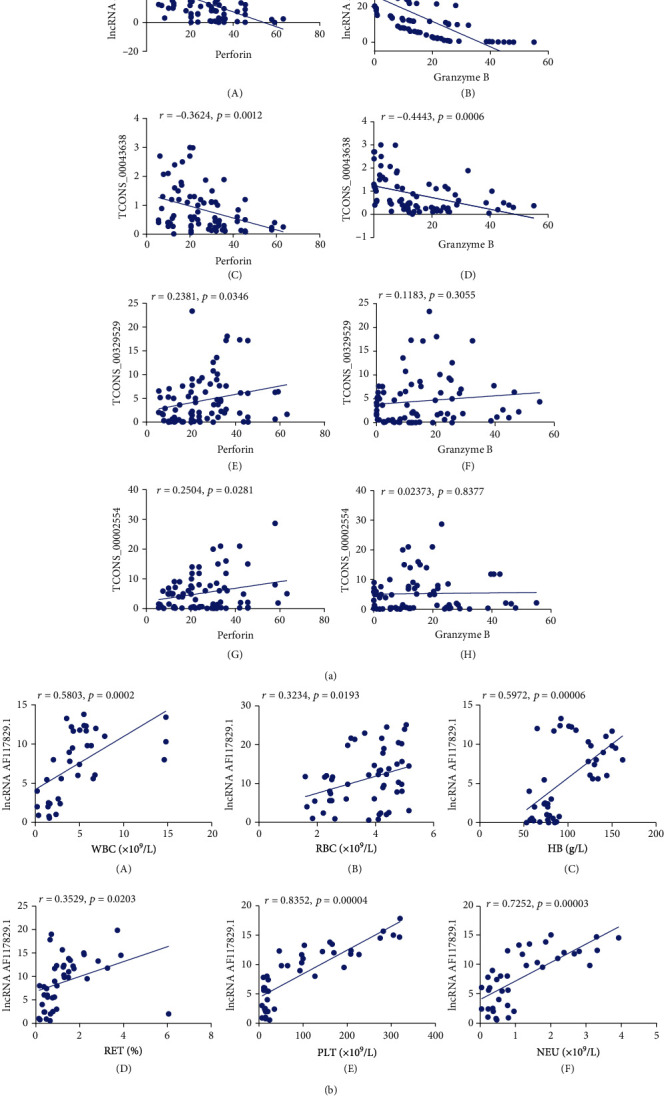
The correlation between the expression of screened lncRNAs, the expression of molecules related to CD8+ T lymphocyte function, and SAA patient clinical characteristics. (a) The correlation between lncRNA expression and perforin and granzyme B expression in the CD8+ T lymphocytes of SAA patients, including lncRNA AF117829.1 (A, B), lncRNA TCONS_00043638 (C, D), lncRNA TCONS_00329529 (E, F), and lncRNA TCONS_00002554 (G, H). (b) The correlation between lncRNA AF117829.1 expression in CD8+ T lymphocytes and the clinical indexes of SAA patients, including WBC (A), RBC (B), Hb (C), RET% (D), PLT (E), and NEU (F).

lncRNA AF117829.1 expression had a negative correlation with perforin and granzyme B expression. lncRNA TCONS_00043638 expression was also negatively correlated with perforin and granzyme B expression. The expression of both lncRNA TCONS_00329529 and lncRNA TCONS_00002554 was positively correlated with the perforin expression but not the granzyme B expression (Figure 4(a)).

### 4.4. The Correlation between lncRNA AF117829.1 Expression in CD8+ T Lymphocytes and Clinical Indexes

The results showed that lncRNA AF117829.1 expression was positively correlated with the clinical indexes of SAA patients, including the white blood cell count (WBC), red blood cell count (RBC), haemoglobin (Hb), percentage of reticulocytes (RET%), platelet count (PLT), and absolute neutrophil count (NEU). All *p* values were less than 0.05 (Figure 4(b)).

### 4.5. RIP2 Signalling Pathway-Related Protein Levels Were Significantly Increased in SAA Patients

Considering the above RT-qPCR results for lncRNAs and mRNAs, we further verified the signalling pathways that might be regulated by lncRNAs. According to the sequencing results, the mRNA expression of RIP2, the predicted target gene of lncRNA AF117829.1, satisfied the differential expression conditions of high-throughput omics (difference > 2-fold, *p* value < 0.05). The downstream targets of RIP2 could regulate the NF-*κ*B and MAPK pathways, which are closely related to T cell proliferation. Therefore, we used western blotting to detect the levels of key proteins, including RIP2, NF-*κ*B, and MAPK. The results showed that the RIP2, NF-*κ*B, and MAPK protein levels were significantly increased in the CD8+ T lymphocytes of SAA patients compared to those of R-SAA patients and healthy controls (Figure 3(c)).

## 5. lncRNA AF117829.1 Mediated the Effect of CD8+ T Lymphocytes in SAA Patients

### 5.1. The Expression of lncRNA AF117829.1 and mRNA RIP2 in lncRNA AF117829.1-Overexpressing CD8+ T Lymphocytes

**Figure 5:**
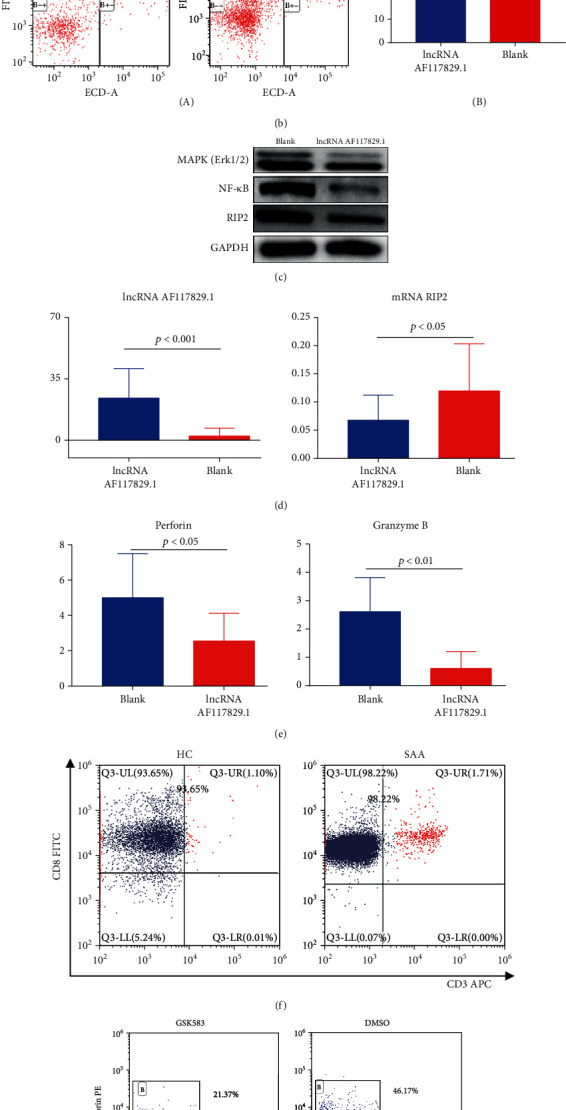
lncRNA AF117829.1 mediated the effect of CD8+ T lymphocytes in SAA patients. (a) FCM to detect the transfection rate of lncRNA AF117829.1 in the CD8+ T lymphocytes of newly diagnosed SAA patients, R-SAA patients, and healthy controls. (b) FCM to detect the apoptosis rate of CD8+ T lymphocytes overexpressing lncRNA AF117829.1 (A), and lncRNA AF117829.1 overexpression suppressed the apoptosis of CD8+ T lymphocytes (B). (c) Western blotting to detect RIP2, NF-*κ*B, and MAPK protein levels in lncRNA AF117829.1-overexpressing CD8+ T lymphocytes. (d) lncRNA AF117829.1 and mRNA RIP2 expression detected by RT-qPCR in the lncRNA AF117829.1-overexpressing CD8+ T lymphocytes. (e) Perforin and granzyme B expression in the lncRNA AF117829.1-overexpressing CD8+ T lymphocytes. (f) The purity of CD8+ T lymphocytes isolated from newly diagnosed SAA patients as detected by FCM. (g) Perforin and granzyme B expression decreased in CD8+ T lymphocytes treated with GSK583, as determined by FCM. (h) RIP2 and MAPK protein levels detected by western blotting in CD8+ T lymphocytes treated with GSK583. (i) The changes in IL-6 and IL-1*β* levels tested by ELISA in the culture supernatants of cells treated with GSK583.

We used FCM to detect the transfection rate of lncRNA AF117829.1 in the CD8+ T lymphocytes of newly diagnosed SAA patients, R-SAA patients, and healthy controls. The transfection rate reached more than 40% (Figure 5(a)). lncRNA AF117829.1 expression was increased in CD8+ T lymphocytes of the lncRNA AF117829.1 lentivirus group (27.36 ± 4.026) compared with those of the blank group (2.375 ± 1.016) (*p* < 0.001). RIP2 mRNA expression was significantly decreased (0.06786 ± 0.0148; 0.1353 ± 0.02897) (*p* < 0.05) (Figure 5(d)).

### 5.2. lncRNA AF117829.1 Overexpression Suppressed the Apoptosis of CD8+ T Lymphocyte

We assessed the changes in the apoptosis rate of CD8+ T lymphocytes transfected with lncRNA AF117829.1 by FCM. The results showed that lncRNA AF117829.1-overexpressing CD8+ T cells had lower apoptosis rates (22.85 ± 3.767%) than blank control cells (38.48 ± 1.566%) (*p* = 0.0086) (Figure 5(b)).

### 5.3. Perforin and Granzyme B Expression in lncRNA AF117829.1-Overexpressing CD8+ T Lymphocytes

Next, we detected perforin and granzyme B expression in CD8+ T lymphocytes transfected with lncRNA AF117829.1 lentivirus by FCM. Perforin and granzyme B expression was significantly downregulated in lncRNA AF117829.1-overexpressing CD8+ T cells (2.549 ± 0.5219%; 0.6118 ± 0.2415%) compared with blank control cells (5.012 ± 0.8285%; 2.549 ± 0.5219%) (*p* = 0.022; *p* = 0.003) (Figure 5(e)).

### 5.4. RIP2, NF-*κ*B, and MAPK Protein Levels in lncRNA AF117829.1-Overexpressing CD8+ T Lymphocytes

The protein levels of RIP2, NF-*κ*B, and MAPK showed a tendency to decrease in the overexpression group compared with the blank group. The RIP2 and NF-*κ*B protein levels were significantly reduced (Figure 5(c)).

### 5.5. Perforin and Granzyme B Expression Decreased in CD8+ T Lymphocytes Treated with GSK583

GSK583, a RIP2 kinase inhibitor, was used to treat CD8+ T lymphocytes collected from the peripheral blood of SAA patients in vitro. MACS was used to sort CD8+ T lymphocytes, and the purity was greater than 90%, as detected by FCM (Figure 5(f)). Perforin expression in GSK583-treated CD8+ T lymphocytes was decreased compared with that in DMSO-treated cells (22.23 ± 2.517%; 35.2 ± 4.237%; *p* = 0.025). The expression of granzyme B tended to decrease but was not significantly different between the two groups (38.63 ± 3.392%; 46.35 ± 6.345%; *p* = 0.33) (Figure 5(g)).

### 5.6. RIP2 and MAPK Protein Levels Were Also Decreased in CD8+ T Lymphocytes Treated with GSK583

Protein was extracted from CD8+ T lymphocytes after GSK583 intervention, and western blotting was used to detect the RIP2 and MAPK protein levels. The results suggested that RIP2 and MAPK were significantly downregulated in CD8+ T lymphocytes treated with GSK583 (Figure 5(h)).

### 5.7. Changes in IL-6 and IL-1*β* in the Culture Supernatants after GSK583 Intervention

Changes in IL-6 and IL-1*β* were detected in the culture supernatants after GSK583 intervention by ELISA. The results showed that the IL-6 levels decreased with GSK583 treatment compared with DMSO treatment (2.95 ± 0.337; 4.538 ± 0.579; *p* = 0.036). The IL-1*β* levels exhibited a decreasing trend but did not show a significant difference (5.793 ± 0.275; 6.575 ± 0.2527; *p* = 0.828) (Figure 5(i)).

## 6. Discussion

SAA is an autoimmune disease, and its immune tolerance mechanism is dysregulated by hyperfunctional T lymphocytes targeting the haematopoietic system [28]. The abnormal immune regulation mechanism is the main pathogenesis mode. First, myeloid dendritic cell (mDC) dysfunction leads to an insufficient number of T regulatory cells (Tregs), which causes Th1/Th2 imbalance. Then, haematopoietic stem/progenitor cells are attacked by activated CD8+ T lymphocytes through the Fas/FasL pathway, perforin, granzyme B, and TNF-*β*, causing serious pancytopenia [29, 30]. CD8+ T lymphocytes play very important roles in the pathogenesis of SAA, which is characterized by an increase in the number and activity of CD8+ T lymphocytes [31, 32]. Moreover, oligoclonal T cell expansion exists in autoimmune diseases such as SLE [33, 34]. Our group detected the expression of tumour necrosis factor-related apoptosis-inducing ligand (TRAIL) in the CD8+ T lymphocytes of SAA patients and found that TRAIL expression was negatively correlated with the expression of perforin and granzyme B in CD8+ T lymphocytes and apoptosis. The TRAIL pathway may activate CD8+ T lymphocytes abnormally and induce the pathogenesis of SAA [35]. The frequency of mutations/polymorphisms of the T cell receptor (TCR)/CD3 signalling complex seems to be decreased in autoimmune disease, chronic inflammation, and malignant tumour states compared to healthy states, which might be caused by T lymphocyte immunodeficiency [36, 37]. The above findings indicate that abnormal immune responses to haematopoietic stem cells/progenitor cells are due to dysregulation of the T cell activation pathway [8].

Based on our previous high-throughput sequencing results and further studies, we found that lncRNA AF117829.1 expression was notably decreased in CD8+ T lymphocytes from patients with SAA, whose function was associated with T lymphocyte proliferation and activation. lncRNAs regulate gene expression independent of protein coding and interact with protein-coding genes in various cell types through multiple processes, such as epigenetic regulation of transcription, mRNA stability, and protein localization [38]. lncRNAs have been shown to be closely related to various immune diseases [39–41]. Downregulation of lncRNA ITSN1-2 is associated with rheumatoid arthritis (RA). lncRNA ITSN1-2 may reduce the proliferation and immunoreaction of fibroblast-like synoviocytes by inhibiting the NOD2/RIP2 signalling pathway in RA patients [42]. Qiu et al. found that lncRNA MEG3 in peripheral blood CD4+ T lymphocytes could affect the balance of Treg/Th17 cells by regulating microRNA-17 in asthma patients [43].

At present, there are few studies on lncRNAs in SAA. In our study, we performed high-throughput sequencing and predicted the target genes of differentially expressed lncRNAs in CD8+ T lymphocytes from patients with SAA. The GO analysis showed that the predicted target genes of lncRNAs were mainly related to signal transduction activity, metabolic process, the response to stimuli, and organelle components. The KEGG analysis showed that the predicted target genes of lncRNAs were related to antigen processing and presentation and the NOD, MAPK, and PKC signalling pathways. The MAPK signalling pathway plays an extremely crucial role in various biological functions, such as T lymphocyte differentiation and proliferation [44]. After activation of the NOD1/NOD2 signalling pathway, the interaction between NOD and RIP2 activates the NF-*κ*B and MAPK pathways, which promotes the activation of proinflammatory cytokine transcription [45]. Our results are consistent with the sequencing results. lncRNA AF117829.1 expression in CD8+ T lymphocytes was significantly decreased in SAA patients compared with healthy controls and was significantly negatively correlated with perforin and granzyme expression. lncRNA TCONS_00043638 expression was negatively correlated with perforin and granzyme B expression. lncRNA TCONS_00329529 and lncRNA TCONS_00002554 expression was positively correlated with perforin expression. This suggests that these lncRNAs could regulate CD8+ T lymphocyte function in the pathogenesis of SAA.

Among these lncRNAs, lncRNA AF117829.1 expression had a significantly negative correlation with perforin and granzyme B expression and a significant positive correlation with the blood cell counts of SAA patients. RIP2, the predicted target gene of lncRNA AF117829.1, is closely related to immunity. The downstream signalling pathway of RIP2 can regulate the NF-*κ*B and MAPK pathways. RIP2 participates in the TCR signalling pathway, activates NF-*κ*B and MAPK signalling, and induces the transcription of inflammatory factors by recruiting TNFR1 and CD14 receptor signalling complexes [46]. However, there are no relevant reports on the mechanism of RIP2 in CD8+ T lymphocytes in SAA. We found that the protein levels of RIP2, NF-*κ*B, and MAPK were significantly increased in CD8+ T lymphocytes in SAA patients. Overexpression of lncRNA AF117829.1 in CD8+ T lymphocytes from SAA patients decreased the expression of mRNA RIP2 and related proteins (RIP2, NF-*κ*B, and MAPK) in the RIP2 signalling pathway. Similarly, perforin and granzyme B expression decreased, and the apoptosis of CD8+ T lymphocytes decreased. Our results suggest that the overexpression of lncRNA AF117829.1 may affect the RIP2 signalling pathway and regulate the apoptosis and inflammation of CD8+ T lymphocytes in SAA. RIP2 kinase is a multidomain, dual-specific kinase [47]. After phosphorylation and ubiquitination, RIP2 kinase can activate NF-*κ*B and MAPK to achieve downstream signal transduction and the transcription of inflammatory cytokines [48, 49]. RIP2 kinase is considered to be a therapeutic target to treat several autoimmune diseases. GSK583 is a highly effective and selective RIP2 kinase inhibitor. Haile et al. demonstrated the effectiveness of GSK583 in blocking downstream NOD2/RIP2 signals in cell models, in vivo animal models, and isolated human disease models [50]. In animal models of experimental colitis, RIP2 kinase inhibitors can effectively reduce inflammation [51]. In our experiments, we found that perforin and granzyme B expression significantly decreased in CD8+ T lymphocytes with GSK583 intervention. In addition, IL-1*β* and IL-6 secretion also decreased.

In summary, lncRNAs are mostly involved in the pathogenesis of diseases through indirect regulation of immune pathogenesis via pathways such as the cytokine-cytokine receptor interaction pathway and the RIP2, NF-*κ*B, and MAPK signalling pathways. In this study, we verified that, at low levels, lncRNA AF117829.1 mediates the RIP2 signalling pathway to activate CD8+ T lymphocyte function and participate in the pathogenesis of SAA immune abnormalities.

## 7. Conclusions

SAA is recognized as an autoimmune disease with immune tolerance dysfunction mediated by hyperactivated T lymphocytes that target the haematopoietic system. Previous studies have shown that the proliferation and overactivation of CD8+ T lymphocytes, also known as CTLs, is a direct factor causing bone marrow failure in SAA patients, but the specific mechanism remains unclear. Our present research displayed the RNA-seq results, possible biological functions, and signalling pathways of CD8+ T lymphocytes from patients with SAA. A total of 194 lncRNAs and 2099 mRNAs exhibited expression changes in the CD8+ T lymphocytes of SAA patients versus R-SAA patients and healthy controls. The predicted target genes of differentially expressed lncRNAs are related to biological processes such as catalytic activity, the response to stimuli, and intra- and extracellular signal transduction. The decreased expression of lncRNA AF117829.1 is closely related to the overactivation of CD8+ T lymphocytes in SAA patients. lncRNA AF117829.1 can regulate the function of CD8+ T lymphocytes by promoting the expression of its target gene RIP2, and the function of CD8+ T lymphocytes is inhibited by treatment with GSK583, a RIP2 kinase inhibitor. Our findings reveal a possible mechanism of lncRNA AF117829.1 in the CD8+ T lymphocytes of patients with SAA, which is helpful for understanding the molecular mechanism of immune pathogenesis and provides potential targets for the diagnosis and treatment of SAA.

## Figures and Tables

**Table 1:** Primer sequences.

Primer	Sequence (5′ to 3′)
*β*-Actin forward	TGGACATCCGCAAAGACCTGT
*β*-Actin reverse	CACACGGAGTACTTGCGCTCA
lncRNA AF117829.1 forward	GGAGTCAGTGTCAGAGTTGGAGTG
lncRNA AF117829.1 reverse	ACCTGTATTGCTTGGCTCATGGC
TCONS_00043638 forward	GAGGACAGAAGGTGGAAGTCAAGC
TCONS_00043638 reverse	AAGACCGCAGAGGAGAGTGAGAC
TCONS_00329529 forward	TCCGAACGCCTCGTGGACTG
TCONS_00329529 reverse	ACAACGCAGAATGAAGGAGGTCAG
TCONS_00002554 forward	AGCAGTGGCTCATGCCTATAATCC
TCONS_00002554 reverse	GAGTTGGAGACCAGCCTGGATAAC
mRNA RIP2 forward	ATTCTGTGATCACAAGACCACT
mRNA RIP2 reverse	CCTTGTAGGCTTGGTACTAACA
mRNA ADAM8 forward	TGAATCACGTGGACAAGCTATA
mRNA ADAM8 reverse	GAACCTGTCCTGACTATTCCAA
mRNA DUOX2 forward	TTGCTCAGGTGCTGGACATCAAC
mRNA DUOX2 reverse	GAAGGACAGGTAGCCATTGCCATC
mRNA BCR forward	CAGCCTATCACCATGACTGACAGC
mRNA BCR reverse	GACTTCGGTGGAGAACAGGATGC

**Table 2:** Clinical data and laboratory values for 4 newly diagnosed SAA patients and 4 healthy controls.

No.	Sex	Age	WBC (×10^9^/L)	RBC (×10^12^/L)	Hb (g/L)	RET (×10^9^/L)	PLT (×10^9^/L)	NEU (×10^9^/L)
SAA1	F	59	1.59	2.61	85	15.3	24	0.47
SAA2	M	24	1.55	1.84	53	4.7	15	0.45
SAA3	F	57	0.36	2.21	68	3.1	17	0.22
SAA4	M	32	2.35	1.65	61	3.6	15	0.37
HC1	F	61	6.86	3.29	84	88.2	166	2.77
HC2	M	33	4.09	4.22	109	54.1	161	4.11
HC3	F	56	5.76	3.16	65	64.8	275	3.31
HC4	M	30	5.53	3.63	104	52.9	319	4.62

**Table 3:** Predicted lncRNA target genes and correlated transcripts.

lncRNA	Acting	Transcript ID	Effect	Gene ID	Regulation
TCONS_00379951	Cis^∗^	ENST00000540020	Promote	ENSG00000104312	Down
TCONS_00043638	Cis^∗^	ENST00000415217	Promote	ENSG00000151651	Down
TCONS_00329529	Cis^∗^	ENST00000606851	Promote	ENSG00000272168	Up
TCONS_00002554	Trans^#^	ENST00000487968	Inhibit	ENSG00000186716	Down
TCONS_00026350	Cis^∗^	ENST00000261465	Promote	ENSG00000117594	Down
TCONS_00380395	Cis^∗^	ENST00000521559	Inhibit	ENSG00000132549	Down
TCONS_00117395	Trans^#^	ENST00000481617	Inhibit	ENSG00000133138	Down

^∗^Cis refers to cis-acting regulation. Cis-acting regulation usually refers to the way in which DNA sequences on the same chromosome directly regulate the expression of other adjacent genes. ^#^Trans refers to trans-acting regulation. Trans-acting regulation is another mechanism by which lncRNAs can regulate target genes. In this mode, lncRNAs and target genes directly recognize each other through base pairing independent of chromosome position.

**Table 4:** Baseline characteristics of SAA and R-SAA patients and healthy controls.

	Sex (M/F)	Median age (range)	WBC (×10^9^/L)	RBC (×10^12^/L)	Hb (g/L)	RET (×10^9^/L)	PLT (×10^9^/L)	NEU (×10^9^/L)
SAA	23/25	44.5 (15-79)	1.53 ± 0.17	1.73 ± 1.13	67.4 ± 2.59	8.93 ± 1.45	40.40 ± 6.13	0.37 ± 0.13
R-SAA	19/14	30 (11-64)	5.01 ± 0.64	4.16 ± 0.56	121 ± 8.13	73.13 ± 9.77	98.67 ± 17.89	2.77 ± 0.54
HC	15/23	38 (20-52)	5.2 ± 0.34	4.39 ± 1.02	117 ± 6.23	77.88 ± 3.55	263 ± 10.57	3.83 ± 1.09

**Table 5:** lncRNA and mRNA expression in CD8+ T lymphocytes as detected by RT-qPCR. The data were represented as the median (25%, 75%).

	SAA	R-SAA	HC
AF117829.1	0.74 (0.431, 1.205)	2.215 (0.8442, 5.136)	6.688 (3.342, 10.32)
RIP2	5.494 (2.144, 30.95)	4.274 (2.398, 9.056)	1.991 (0.4553, 4.655)
TCONS_00043638	0.3 (0.15, 0.5)	0.6373 (0.4093, 1.15)	1.6 (0.6, 2.4)
ADAM8	3.327 (1.678, 4.467)	2.454 (1.624, 4.226)	2.328 (0.5487, 2.98)
TCONS_00329529	4.691 (1.873, 10.79)	3.9 (0.2907, 6.495)	1.739 (0.152, 5.036)
DUOX2	6.923 (3.937, 9.961)	3.443 (1, 8.3)	2.17 (1.075, 4.175)

## Data Availability

The RNA-seq data used to support the findings of this study are included within the article and the supplementary information files. And the RNA-seq data used to support the findings of this study are available from the corresponding author upon request.

## Supplementary Materials

Supplementary 1 Supplementary file S1: the RNA-seq results of 2099 differentially expressed mRNAs (1104 upregulated and 995 downregulated mRNAs) in CD8+ T lymphocytes from SAA patients, including transcript ID, its length, log2 fold change, *p* values, adjusted *p* values, expression (up- or downregulation), and gene ID and the corresponding gene symbol.

Supplementary 2 Supplementary file S2: the RNA-seq results of 194 differentially expressed lncRNAs (107 upregulated and 87 downregulated lncRNAs) in CD8+ T lymphocytes from SAA patients, including lncRNA ID, its length, log2 fold change, *p* values, adjusted *p* values, and expression (up- or downregulation).
