# Supplementary material for: The Role of lncRNA AF117829.1 in the Immunological Pathogenesis of Severe Aplastic Anaemia
Source: Oxid Med Cell Longev. 2021 Mar 16;2021:5587921. doi: 10.1155/2021/5587921 (PMC10435305; doi:10.1155/2021/5587921)
Supplement: Supplementary 1 — Supplementary file S1: the RNA-seq results of 2099 differentially expressed mRNAs (1104 upregulated and 995 downregulated mRNAs) in CD8+ T lymphocytes from SAA patients, including transcript ID, its length, log2 fold change, p values, adjusted p values, expression (up- or downregulation), and gene ID and the corresponding gene symbol. [file 5587921.f1.pdf]

gene_id	length	log2FoldChange	pvalue	padj	Regulation	geneID	GeneSymbol
ENST00000243997	3665	-6.680328058	1.09E-14	9.06E-11	Down	ENSG00000124172	ATP5E
ENST00000352127	2563	-8.605542587	1.29E-14	9.06E-11	Down	ENSG00000126215	XRCC3
ENST00000395663	697	-7.373410752	2.34E-14	1.1E-10	Down	ENSG00000124172	ATP5E
ENST00000430065	2483	-7.338015822	1.42E-09	0.00000498	Down		
ENST00000495204	4294	6.950760215	5.78E-08	0.000162752	Ups	ENSG00000076864	RAP1GAP
ENST00000594368	1471	-6.600842317	7.69E-08	0.000180448	Down	ENSG00000063322	MED29
ENST00000589228	4256	6.682185626	0.000000229	0.000460097	Ups	ENSG00000108946	PRKAR1A
ENST00000515829	5629	6.589779639	0.000000266	0.000467638	Ups	ENSG00000116698	SMG7
ENST00000506152	3531	-6.049783935	0.000000491	0.000767269	Down	ENSG00000237075	RP11-497H16.2
ENST00000360635	4859	-3.837799724	0.000000678	0.000954274	Down	ENSG00000119185	ITGB1BP1
ENST00000395044	1807	4.38406634	0.000000798	0.000980962	Ups	ENSG00000213585	VDAC1
ENST00000400275	741	6.473889638	0.000000837	0.000980962	Ups	ENSG00000215414	PSMA6P1
ENST00000538486	2819	6.329518935	0.00000157	0.001704803	Ups	ENSG00000157110	RBPMS
ENST00000595437	4926	6.282368231	0.00000214	0.002145988	Ups	ENSG00000152767	FARP1
ENST00000509075	1180	-6.175545052	0.00000307	0.002884117	Down	ENSG00000250918	RP11-497H16.4
ENST00000288774	2038	-1.623110789	0.00000398	0.003504174	Down	ENSG00000157911	PEX10
ENST00000342210	3005	-5.90397608	0.00000428	0.003541434	Down	ENSG00000187554	TLR5
ENST00000357771	2313	5.872618409	0.00000539	0.00409262	Ups	ENSG00000131233	GJA9
ENST00000465161	4617	3.160218586	0.00000581	0.00409262	Ups		
ENST00000528080	3968	6.029897055	0.00000591	0.00409262	Ups	ENSG00000166471	TMEM41B
ENST00000418290	1513	6.047126643	0.00000611	0.00409262	Ups	ENSG00000228411	CDY4P
ENST00000308739	498	5.958840674	0.00000822	0.005257304	Ups	ENSG00000230593	AC090804.1
ENST00000527717	1799	-4.87410806	0.0000164	0.010047197	Down	ENSG00000137726	FXYD6
ENST00000347615	5788	-5.722239772	0.0000213	0.01185772	Down	ENSG00000116698	SMG7
ENST00000598222	1950	1.961187308	0.000022	0.01185772	Ups	ENSG00000268852	AC132872.2
ENST00000429828	3286	5.730236698	0.0000224	0.01185772	Ups	ENSG00000188199	NUTM2B
ENST00000457710	7304	-2.113591468	0.0000228	0.01185772	Down	ENSG00000004139	SARM1
ENST00000374316	9870	-5.711083392	0.000024	0.012058502	Down	ENSG00000096433	ITPR3
ENST00000372360	510	5.528993855	0.0000282	0.013681422	Ups	ENSG00000138326	RPS24
ENST00000430238	911	5.419927618	0.000033	0.01547811	Ups		
ENST00000503865	1678	5.443889844	0.0000457	0.020747148	Ups	ENSG00000092421	SEMA6A
ENST00000251607	2276	5.624686568	0.000048	0.020770835	Ups	ENSG00000072756	TRNT1
ENST00000252595	3594	-3.702246857	0.0000487	0.020770835	Down	ENSG00000130304	SLC27A1
ENST00000315658	2685	-3.534157445	0.000051	0.020904104	Down	ENSG00000115459	ELMOD3
ENST00000609136	2046	3.25663873	0.0000523	0.020904104	Ups		
ENST00000557826	587	3.214929306	0.0000535	0.020904104	Ups	ENSG00000166411	IDH3A

ENST00000380759	1334	3.157904568	0.0000568	0.021592196	Ups	ENSG00000253203	GUSBP3
ENST00000468886	2508	-5.325240143	0.0000668	0.02346651	Down	ENSG00000137878	GCOM1
ENST00000368445	3472	-2.345725622	0.0000683	0.02346651	Down	ENSG00000160691	SHC1
ENST00000329134	4325	5.427899171	0.0000686	0.02346651	Ups	ENSG00000183878	UTY
ENST00000434240	2242	2.824947287	0.0000693	0.02346651	Ups	ENSG00000230638	RP11-486B10.4
ENST00000576853	4314	3.130647512	0.00007	0.02346651	Ups		
ENST00000529435	1011	5.37562269	0.0000782	0.025583024	Ups	ENSG00000166012	TAF1D
ENST00000456530	2500	3.381595588	0.0000922	0.029477727	Ups	ENSG00000174748	RPL15
ENST00000285737	8199	1.601631033	0.00011373	0.035564717	Ups	ENSG00000102910	LONP2
ENST00000336509	2538	5.30433302	0.000128356	0.0383318	Ups	ENSG00000092931	MFSD11
ENST00000559896	705	-4.108547294	0.000130349	0.0383318	Down	ENSG00000259174	RP11-1180F24.1
ENST00000264346	3779	-5.240320083	0.000130751	0.0383318	Down	ENSG00000138642	HERC6
ENST00000467294	3113	-1.912527669	0.000146716	0.042134549	Down	ENSG00000114388	NPRL2
ENST00000541105	1682	5.194824522	0.000160551	0.045185543	Ups	ENSG00000175336	APOF
ENST00000406369	4589	5.244844171	0.000171021	0.04718848	Ups	ENSG00000152683	SLC30A6
ENST00000539054	2977	-5.027945436	0.000207027	0.053623701	Down	ENSG00000239264	TXNDC5
ENST00000336452	1248	-5.025321006	0.000207794	0.053623701	Down	ENSG00000132507	EIF5A
ENST00000502752	1584	3.058828025	0.000208733	0.053623701	Ups	ENSG00000151422	FER
ENST00000256319	2633	3.738210622	0.000211596	0.053623701	Ups	ENSG00000133935	C14orf1
ENST00000265970	8227	3.685143159	0.000213397	0.053623701	Ups	ENSG00000011405	PIK3C2A
ENST00000418231	1950	2.228618149	0.000230487	0.056044631	Ups	ENSG00000232928	DDX3YP1
ENST00000590918	3289	3.196342554	0.000230997	0.056044631	Ups	ENSG00000010310	GIPR
ENST00000379846	1962	5.120047838	0.000240539	0.057370702	Ups	ENSG00000139631	CSAD
ENST00000262482	1915	4.980116231	0.000251117	0.058895413	Ups	ENSG00000108523	RNF167
ENST00000324366	2531	-4.949345292	0.00025197	NA	Down	ENSG00000100462	PRMT5
ENST00000420021	3895	-5.123379484	0.000283509	0.065402195	Down	ENSG00000230246	SPATA31C1
ENST00000259056	6171	3.163018451	0.000292377	0.065438154	Ups	ENSG00000136542	GALNT5
ENST00000567149	497	2.233136148	0.000297567	0.065438154	Ups	ENSG00000260229	RP11-391L3.5
ENST00000378150	2457	4.908531914	0.000297615	0.065438154	Ups	ENSG00000152465	NMT2
ENST00000547575	3122	4.93875988	0.000309123	0.06692276	Ups	ENSG00000173588	CCDC41
ENST00000338784	1971	-3.523290899	0.000326331	0.069577728	Down	ENSG00000161955	TNFSF13
ENST00000580571	952	-4.953933995	0.000341511	0.071727501	Down	ENSG00000125457	MIF4GD
ENST00000370501	4132	4.86928419	0.000356594	0.072904739	Ups	ENSG00000101188	NTSR1
ENST00000481804	3248	-1.737667026	0.000357478	0.072904739	Down	ENSG00000162004	CCDC78
ENST00000603779	1070	1.898160635	0.000376573	0.075701926	Ups	ENSG00000270772	RP11-332H21.2
ENST00000459650	1639	1.873396094	0.00040853	0.080969543	Ups	ENSG00000244556	ODCP
ENST00000398922	1368	4.898698075	0.000417376	0.081103162	Ups	ENSG00000214756	METTL12

ENST00000470649	760	1.614416396	0.000420731	0.081103162	Ups	ENSG00000011295	TTC19
ENST00000554327	1965	4.970892091	0.000433413	0.081533405	Ups	ENSG00000135424	ITGA7
ENST00000590400	1812	3.7395297	0.000436007	0.081533405	Ups	ENSG00000197256	KANK2
ENST00000381641	758	4.86027647	0.000444133	0.081533405	Ups	ENSG00000120210	INSL6
ENST00000519196	689	2.801670869	0.000448818	0.081533405	Ups	ENSG00000094755	GABRP
ENST00000356962	1468	4.881059926	0.000455119	0.081533405	Ups	ENSG00000118520	ARG1
ENST00000543538	6992	3.151461026	0.000457727	0.081533405	Ups	ENSG00000163697	APBB2
ENST00000602775	1466	4.910187575	0.00046788	0.082300038	Ups	ENSG00000213523	SRA1
ENST00000475443	731	3.963210714	0.000499529	0.086782382	Ups	ENSG00000166171	DPCD
ENST00000341369	1212	4.769348282	0.000522026	0.089584754	Ups	ENSG00000187514	PTMA
ENST00000610153	2070	-4.901202839	0.000532362	0.090257838	Down	ENSG00000118518	RNF146
ENST00000428060	556	4.800816052	0.000575655	0.096126057	Ups	ENSG00000223915	DPPA2P1
ENST00000572949	7862	-4.402563619	0.000585366	0.096126057	Down	ENSG00000129204	USP6
ENST00000361136	4477	-4.259250389	0.000587467	0.096126057	Down	ENSG00000196428	TSC22D2
ENST00000534313	3521	-4.77261407	0.000640651	0.103623456	Down	ENSG00000213445	SIPA1
ENST00000396976	5595	-4.770834139	0.000653742	0.104539336	Down	ENSG00000140265	ZSCAN29
ENST00000298173	1677	-3.73268975	0.000676987	0.107040008	Down	ENSG00000165417	GTF2A1
ENST00000580202	534	4.768270129	0.000686953	0.10710564	Ups		
ENST00000366709	19907	4.779915546	0.000692625	0.10710564	Ups	ENSG00000154358	OBSCN
ENST00000463609	554	1.652744224	0.00082582	0.126314543	Ups	ENSG00000198947	DMD
ENST00000584501	2251	2.597149858	0.000890259	0.133288692	Ups	ENSG00000141750	STAC2
ENST00000370267	1647	-4.735333826	0.000890359	0.133288692	Down	ENSG00000117505	DR1
ENST00000468293	737	-3.378934555	0.000921513	0.135206287	Down	ENSG00000164867	NOS3
ENST00000579280	498	-4.701402775	0.000931139	0.135206287	Down	ENSG00000175224	ATG13
ENST00000346246	4271	-4.346022304	0.000931993	0.135206287	Down	ENSG00000179361	ARID3B
ENST00000608786	738	-3.170906966	0.000988149	0.140887694	Down	ENSG00000101608	MYL12A
ENST00000263276	3378	-2.742721106	0.00099441	0.140887694	Down	ENSG00000104812	GYS1
ENST00000571656	1045	3.014238756	0.001001192	0.140887694	Ups		
ENST00000521462	1465	-4.644533854	0.001020555	NA	Down	ENSG00000179921	GPBAR1
ENST00000392794	808	2.474811604	0.001028716	0.143188423	Ups	ENSG00000213188	YBX1P4
ENST00000391480	1710	-1.039803354	0.001058104	0.143188423	Down	ENSG00000184682	C11orf89
ENST00000440192	890	3.996622752	0.001061253	0.143188423	Ups	ENSG00000226705	SDCBPP1
ENST00000454295	4376	4.626829211	0.001062934	0.143188423	Ups	ENSG00000152939	MARVELD2
ENST00000573058	1627	2.733869098	0.001068418	0.143188423	Ups	ENSG00000157637	SLC38A10
ENST00000336617	1629	-4.647485691	0.001078685	0.143200515	Down	ENSG00000136104	RNASEH2B
ENST00000574731	5320	-4.725477545	0.001100781	0.144421465	Down		
ENST00000464301	571	-3.516441164	0.001124977	0.144421465	Down	ENSG00000196950	SLC39A10

ENST00000535028	3886	3.562802228	0.001126514	0.144421465	Ups	ENSG00000177239	MAN1B1
ENST00000580086	2638	1.290802452	0.001128934	0.144421465	Ups	ENSG00000188895	MSL1
ENST00000425172	596	-2.814996493	0.00115762	0.146756999	Down	ENSG00000186480	INSIG1
ENST00000603815	3167	-4.567198525	0.001206995	NA	Down	ENSG00000197111	PCBP2
ENST00000395069	2059	3.309504523	0.001212646	0.152360329	Ups	ENSG00000178401	DNAJC22
ENST00000464807	566	-4.525201232	0.001298519	NA	Down	ENSG00000099949	LZTR1
ENST00000344135	4065	3.59424904	0.001304408	0.162143329	Ups	ENSG00000038382	TRIO
ENST00000299353	2670	4.550639884	0.001323272	0.162143329	Ups	ENSG00000270316	C10orf32-ASMT
ENST00000559289	864	2.406221133	0.001325077	0.162143329	Ups	ENSG00000185634	SHC4
ENST00000338832	1541	2.547132385	0.001349482	0.162733014	Ups	ENSG00000188771	PLET1
ENST00000569689	786	-3.402893738	0.001353025	0.162733014	Down	ENSG00000167977	KCTD5
ENST00000446488	5093	4.584030708	0.001411351	0.167791224	Ups	ENSG00000170322	NFRKB
ENST00000551043	2288	-4.545791249	0.001413454	NA	Down	ENSG00000111596	CNOT2
ENST00000406819	1057	1.816008644	0.001418928	0.167791224	Ups	ENSG00000219249	AMZ2P2
ENST00000361901	4114	4.433677323	0.001428113	NA	Ups	ENSG00000122786	CALD1
ENST00000470889	3161	2.067052049	0.001445736	0.169536632	Ups	ENSG00000158234	FAIM
ENST00000358344	1796	4.534068267	0.001477624	0.170966205	Ups	ENSG00000197451	HNRNPAB
ENST00000439961	304	2.381007601	0.001482225	0.170966205	Ups	ENSG00000235082	SUMO1P3
ENST00000374550	634	-2.134415968	0.001507178	0.171280394	Down	ENSG00000142676	RPL11
ENST00000462572	1280	4.031635945	0.001509293	0.171280394	Ups	ENSG00000241136	PAICSP6
ENST00000397685	1931	-4.506053671	0.001515853	NA	Down	ENSG00000160298	C21orf58
ENST00000445935	1931	-4.506053671	0.001515853	NA	Down	ENSG00000160298	C21orf58
ENST00000553700	9080	-4.521670215	0.001530671	NA	Down	ENSG00000092148	HECTD1
ENST00000308158	2038	-4.471065547	0.001533989	NA	Down	ENSG00000175309	PHYKPL
ENST00000535325	4635	3.409698411	0.001542313	0.173627478	Ups	ENSG00000163820	FYCO1
ENST00000581220	2384	4.40385682	0.001567941	NA	Ups	ENSG00000101782	RIOK3
ENST00000272559	1479	-3.081734735	0.001584341	0.176943207	Down	ENSG00000144152	FBLN7
ENST00000260746	3844	1.421849964	0.001620433	0.17789623	Ups	ENSG00000138175	ARL3
ENST00000569217	814	-4.481665278	0.001623968	NA	Down	ENSG00000178952	TUFM
ENST00000437827	1995	3.3325662	0.001626634	0.17789623	Ups	ENSG00000163788	SNRK
ENST00000343214	2395	-4.577614983	0.001651038	0.17789623	Down	ENSG00000106348	IMPDH1
ENST00000337227	3861	1.680643678	0.001654428	0.17789623	Ups	ENSG00000089050	RBBP9
ENST00000299397	2326	-4.587278876	0.001656083	0.17789623	Down	ENSG00000166311	SMPD1
ENST00000536790	2328	4.464378414	0.001686919	0.179835805	Ups	ENSG00000196387	ZNF140
ENST00000480906	1802	2.18781288	0.001701069	0.179980754	Ups	ENSG00000114331	ACAP2
ENST00000441671	1576	-3.34451246	0.001744205	0.183167559	Down	ENSG00000089692	LAG3
ENST00000509887	1186	-4.500598943	0.001765724	0.183392025	Down	ENSG00000164211	STARD4

ENST00000577831	4584	4.438216917	0.001772407	0.183392025	Ups	ENSG00000125686	MED1
ENST00000429492	7723	1.666461569	0.001798387	0.184721948	Ups	ENSG00000144645	OSBPL10
ENST00000301096	2599	-4.407638605	0.001830987	NA	Down	ENSG00000167766	ZNF83
ENST00000512224	4335	-2.496108697	0.001832083	0.186819327	Down	ENSG00000205565	RP11-497H16.6
ENST00000564146	985	4.444585782	0.001860724	0.188374851	Ups	ENSG00000225526	C3orf83
ENST00000538262	612	-4.45303678	0.001877689	NA	Down	ENSG00000244716	RP11-20O24.4
ENST00000368035	1463	-2.670600535	0.00191306	0.192289909	Down	ENSG00000122224	LY9
ENST00000423386	429	4.343316562	0.001924123	NA	Ups	ENSG0000238168	RP11-686G8.1
ENST00000493321	1134	4.378116224	0.00198069	NA	Ups	ENSG00000140403	DNAJA4
ENST00000569540	2331	4.352346453	0.001985012	NA	Ups	ENSG00000166822	TMEM170A
ENST00000449428	1881	2.545853087	0.002008333	0.199657343	Ups	ENSG00000182534	MXRA7
ENST00000496353	2428	-2.196740117	0.002014734	0.199657343	Down	ENSG00000127054	CPSF3L
ENST00000255152	2773	-4.406205587	0.002046234	NA	Down	ENSG00000132801	ZSWIM3
ENST00000535008	3174	4.39567192	0.002053947	0.201775292	Ups	ENSG00000187416	LHFPL3
ENST00000581503	5586	3.181998651	0.002064784	0.201775292	Ups		
ENST00000357903	3543	-4.435064304	0.00208238	NA	Down	ENSG00000142192	APP
ENST00000341702	1311	1.990501896	0.002095411	0.203356046	Ups	ENSG00000196131	VN1R2
ENST00000525640	2523	-4.504313423	0.002110869	0.203453111	Down	ENSG00000136874	STX17
ENST00000547344	2474	-4.342052329	0.00216138	NA	Down	ENSG00000111012	CYP27B1
ENST00000564000	1088	-2.844640307	0.002183451	0.209017117	Down	ENSG00000103254	FAM173A
ENST00000398581	564	-2.388243202	0.002213342	0.210446961	Down	ENSG00000213860	RPL21P75
ENST00000447648	6564	1.636823624	0.002237592	0.211324812	Ups	ENSG00000205356	TECPR1
ENST00000494426	1017	-3.501805269	0.002279483	0.213845899	Down	ENSG00000169583	CLIC3
ENST00000577503	1167	-4.397214884	0.002303343	NA	Down		
ENST00000395169	4107	4.385775961	0.002367281	0.219032665	Ups	ENSG00000109339	MAPK10
ENST00000487283	3273	2.88883925	0.002373254	0.219032665	Ups	ENSG00000115085	ZAP70
ENST00000594385	1484	-2.559554747	0.002381467	0.219032665	Down	ENSG00000197013	ZNF429
ENST00000412997	2587	4.33513561	0.002427166	NA	Ups	ENSG00000129810	SGOL1
ENST00000430725	2944	-1.419239631	0.00243314	0.220897731	Down	ENSG00000179364	PACS2
ENST00000605330	2944	-1.419239631	0.00243314	0.220897731	Down		
ENST00000493354	4616	-3.631398783	0.002461003	NA	Down		
ENST00000584176	6516	1.567539391	0.002482259	0.223826239	Ups	ENSG00000161509	GRIN2C
ENST00000369781	2082	-4.440052968	0.00250121	0.223826239	Down	ENSG00000168679	SLC16A4
ENST00000479114	832	4.266815802	0.002522163	NA	Ups	ENSG00000165209	STRBP
ENST00000604882	1037	-1.235149626	0.002533116	0.223826239	Down	ENSG00000271043	MTRNR2L2
ENST00000368802	10701	-4.425026307	0.002537233	0.223826239	Down	ENSG00000009413	REV3L
ENST00000336314	6595	-2.915472406	0.002544926	0.223826239	Down	ENSG00000155506	LARP1

ENST00000506487	634	2.749173425	0.0026177	0.227775066	Ups	ENSG00000114686	MRPL3
ENST00000491847	5042	-1.746308712	0.002634513	0.227775066	Down	ENSG00000114737	CISH
ENST00000306507	2068	-3.384297323	0.002638384	0.227775066	Down	ENSG00000184492	FOXD4L1
ENST00000555408	2598	-4.317515364	0.002658745	NA	Down	ENSG00000123374	CDK2
ENST00000335012	5901	2.747191763	0.002667877	0.228916826	Ups	ENSG00000187068	C3orf70
ENST00000422127	24030	-2.802259429	0.002697451	0.230051738	Down	ENSG00000154358	OBSCN
ENST00000585553	2241	4.304539552	0.002724608	NA	Ups	ENSG00000181513	ACBD4
ENST00000504137	2877	-3.635548963	0.002745396	0.232730154	Down	ENSG00000237765	FAM200B
ENST00000445820	512	-2.644326502	0.002768048	0.233245328	Down		
ENST00000314100	8486	1.614853711	0.002813018	0.234773034	Ups	ENSG00000183826	BTBD9
ENST00000326219	597	-4.376543935	0.002831418	0.234773034	Down	ENSG00000176358	TAC4
ENST00000541602	4427	3.015767094	0.002836229	0.234773034	Ups	ENSG00000109906	ZBTB16
ENST00000332142	4113	2.89357096	0.002857235	0.235128712	Ups	ENSG00000184611	KCNH7
ENST00000244576	4079	3.465478012	0.002874205	NA	Ups	ENSG00000124613	ZNF391
ENST00000477715	2169	-3.121864232	0.002896986	0.236524118	Down	ENSG00000145757	SPATA9
ENST00000491053	590	-1.532660257	0.002907808	0.236524118	Down	ENSG00000136878	USP20
ENST00000522290	1614	-4.234468791	0.002934263	NA	Down	ENSG00000061337	LZTS1
ENST00000477327	3608	2.498608235	0.002985633	0.240416462	Ups	ENSG00000128708	HAT1
ENST00000510685	716	2.170633437	0.003033608	0.240416462	Ups	ENSG00000053108	FSTL4
ENST00000567244	723	4.203534056	0.00303716	NA	Ups	ENSG00000103051	COG4
ENST00000262352	3722	2.34783813	0.003058218	0.240416462	Ups	ENSG00000106688	SLC1A1
ENST00000416336	1350	-3.468032233	0.003075253	0.240416462	Down		
ENST00000429592	1350	-3.468032233	0.003075253	0.240416462	Down		
ENST00000436988	1350	-3.468032233	0.003075253	0.240416462	Down		
ENST00000446599	1350	-3.468032233	0.003075253	0.240416462	Down		
ENST00000551887	760	-4.253788922	0.003083069	NA	Down	ENSG00000139626	ITGB7
ENST00000521331	1087	4.185803105	0.003115025	NA	Ups	ENSG00000253729	PRKDC
ENST00000255977	3151	-3.525953183	0.003127415	NA	Down	ENSG00000133606	MKRN1
ENST00000583249	544	4.247992415	0.003134747	NA	Ups	ENSG00000175224	ATG13
ENST00000469543	2666	-2.247377274	0.003148799	0.244806083	Down	ENSG00000026025	VIM
ENST00000382192	2062	4.234473085	0.003197682	NA	Ups	ENSG00000122386	ZNF205
ENST00000553901	3395	1.246760957	0.003207299	0.246794545	Ups	ENSG00000151327	FAM177A1
ENST00000372638	1290	-4.238005586	0.003208031	NA	Down	ENSG00000179862	CITED4
ENST00000601142	2280	-4.340105039	0.003209452	0.246794545	Down	ENSG00000168661	ZNF30
ENST00000521006	4656	-3.768070928	0.003267388	0.249884174	Down	ENSG00000095539	SEMA4G
ENST00000464716	452	-4.225594371	0.00329516	NA	Down	ENSG00000138326	RPS24
ENST00000490569	1802	4.240251208	0.003300218	NA	Ups	ENSG00000156508	EEF1A1

ENST00000592474	1898	2.407679047	0.003321054	0.251144377	Ups	ENSG00000161542	PRPSAP1
ENST00000586052	6551	3.352483481	0.003332953	0.251144377	Ups	ENSG00000187775	DNAH17
ENST00000303846	2971	-2.319449186	0.003337408	0.251144377	Down	ENSG00000170374	SP7
ENST00000490188	647	-4.181818936	0.003361767	NA	Down	ENSG00000074582	BCS1L
ENST00000263125	3285	4.230961985	0.003386872	NA	Ups	ENSG00000065675	PRKCQ
ENST00000488088	695	1.444315924	0.003393285	0.252371197	Ups	ENSG00000130052	STARD8
ENST00000258411	2547	-3.747546501	0.003403158	0.252371197	Down	ENSG00000135925	WNT10A
ENST00000256649	3515	4.309582159	0.003407513	0.252371197	Ups	ENSG00000134253	TRIM45
ENST00000530776	546	-4.319754056	0.003426859	0.252475169	Down	ENSG00000147789	ZNF7
ENST00000503216	1171	-4.309458859	0.003463968	0.253879998	Down	ENSG00000169230	PRELID1
ENST00000341776	5755	1.764366087	0.00354631	0.258568255	Ups	ENSG00000008083	JARID2
ENST00000350274	3165	1.494874504	0.003568581	0.258850914	Ups	ENSG00000134539	KLRD1
ENST00000339437	976	4.201702528	0.00360643	NA	Ups	ENSG00000072756	TRNT1
ENST00000581767	2244	2.151101696	0.00362692	0.259514619	Ups	ENSG00000198231	DDX42
ENST00000559199	2059	1.867859997	0.00363892	0.259514619	Ups	ENSG00000090487	SPG21
ENST00000305364	4229	-2.666042465	0.003648386	0.259514619	Down	ENSG00000104228	TRIM35
ENST00000568681	1739	3.126035702	0.003711481	0.262452058	Ups	ENSG00000090238	YPEL3
ENST00000461599	1801	4.194089765	0.003745671	NA	Ups	ENSG00000241627	UBQLN4P1
ENST00000395629	3180	4.266922126	0.003763419	0.262544319	Ups	ENSG00000121653	MAPK8IP1
ENST00000496770	837	-3.490843893	0.003786883	NA	Down	ENSG00000160097	FNDC5
ENST00000442855	241	3.483229586	0.003792861	NA	Ups	ENSG00000234784	RP11-182I10.1
ENST00000292852	2383	2.961528229	0.003832672	0.262544319	Ups	ENSG00000269190	FBXO17
ENST00000535833	6073	-1.768576154	0.003832998	0.262544319	Down	ENSG00000176020	AMIGO3
ENST00000474014	1933	1.891837837	0.003840529	0.262544319	Ups	ENSG00000138162	TACC2
ENST00000301698	1209	-3.249903196	0.003848455	0.262544319	Down	ENSG00000167945	PRR25
ENST00000368917	3228	4.25990102	0.003868415	0.262544319	Ups	ENSG00000143458	GABPB2
ENST00000607192	456	4.175937679	0.003871162	NA	Ups		
ENST00000368358	3718	-3.787598472	0.003871644	0.262544319	Down	ENSG00000143630	HCN3
ENST00000575670	3718	-3.787598472	0.003871644	0.262544319	Down		
ENST00000219700	1621	-3.649346583	0.0038862	0.262544319	Down	ENSG00000103415	HMOX2
ENST00000493474	590	-3.603740127	0.003909624	NA	Down	ENSG00000168297	PXK
ENST00000428003	681	4.092817375	0.003911337	NA	Ups	ENSG00000185842	DNAH14
ENST00000542678	6051	-1.266960299	0.003932984	0.262544319	Down	ENSG00000150967	ABCB9
ENST00000391788	4209	-4.243987647	0.003944797	0.262544319	Down	ENSG00000167555	ZNF528
ENST00000371868	2266	-2.560084804	0.003954364	0.262544319	Down	ENSG00000123453	SARDH
ENST00000596694	2719	3.477554442	0.003954507	NA	Ups	ENSG00000204869	IGFL4
ENST00000467228	2454	3.123041652	0.003955329	0.262544319	Ups	ENSG00000182902	SLC25A18

ENST00000582530	2117	-4.238309483	0.00399878	NA	Down	ENSG00000224383	C17orf72
ENST00000325795	2692	3.751567452	0.004011369	0.263853835	Ups	ENSG00000128699	ORMDL1
ENST00000554208	2458	2.107316371	0.004012558	0.263853835	Ups	ENSG00000072415	MPP5
ENST00000273588	2276	-2.919438281	0.004055738	0.264842732	Down	ENSG00000145020	AMT
ENST00000223129	2020	-1.700715574	0.00407088	0.264842732	Down	ENSG00000106399	RPA3
ENST00000499077	2208	3.22716974	0.004084059	0.264842732	Ups	ENSG00000246082	NUDT16P1
ENST00000545067	3998	-1.36495942	0.004119838	0.265937405	Down	ENSG00000216937	CCDC7
ENST00000425226	1451	3.096309867	0.004157335	0.266953478	Ups	ENSG00000233579	KRT8P15
ENST00000605168	568	1.576480905	0.004173519	0.266953478	Ups	ENSG00000270832	RP1-168P16.3
ENST00000469999	1143	1.670090658	0.004214826	0.268375699	Ups	ENSG00000241007	SEPT7P6
ENST00000366919	5397	-3.471667275	0.004285141	NA	Down	ENSG00000085511	MAP3K4
ENST00000409239	776	-4.089175122	0.004349806	NA	Down	ENSG00000255552	LY6G6E
ENST00000517958	3149	3.27613618	0.004367714	0.276857992	Ups	ENSG00000164574	GALNT10
ENST00000373580	6425	-4.128253157	0.004380939	NA	Down	ENSG00000185585	OLFML2A
ENST00000467942	3722	-1.79661093	0.004425751	0.278032008	Down	ENSG00000114529	C3orf52
ENST00000469387	818	-2.359733753	0.004466073	0.279318137	Down	ENSG00000079335	CDC14A
ENST00000561857	2077	2.652765567	0.004486587	0.279359543	Ups	ENSG00000197471	SPN
ENST00000502974	630	-4.118091235	0.004520895	NA	Down	ENSG00000163734	CXCL3
ENST00000460018	575	-2.123390379	0.004525324	0.280530208	Down	ENSG00000204344	STK19
ENST00000228862	3568	1.077520254	0.004573589	0.282278683	Ups	ENSG00000111266	DUSP16
ENST00000602907	997	2.120384538	0.00461944	0.282349575	Ups	ENSG00000248863	RP11-83A24.1
ENST00000216264	4450	2.567508875	0.004634137	0.282349575	Ups	ENSG00000100422	CERK
ENST00000463472	753	-2.476717268	0.004654888	0.282349575	Down	ENSG00000177989	ODF3B
ENST00000421365	1617	-2.772355029	0.004654996	0.282349575	Down	ENSG00000115267	IFIH1
ENST00000535425	1002	-3.388028123	0.004692287	0.283389973	Down	ENSG00000130489	SCO2
ENST00000427250	1606	-1.671210367	0.004754207	0.284685971	Down	ENSG00000105699	LSR
ENST00000605618	1606	-1.671210367	0.004754207	0.284685971	Down	ENSG00000105699	LSR
ENST00000518216	12783	-4.097909866	0.004761895	NA	Down	ENSG00000253729	PRKDC
ENST00000467218	2739	-1.425474378	0.004776713	0.284821619	Down	ENSG00000100003	SEC14L2
ENST00000568546	6803	-2.695960701	0.004833206	0.286854969	Down	ENSG00000183751	TBL3
ENST00000359599	9411	4.097664165	0.004835758	NA	Ups	ENSG00000154358	OBSCN
ENST00000289805	2335	-2.091267217	0.004851583	0.286854969	Down	ENSG00000158792	SPATA2L
ENST00000355086	8943	-4.158421669	0.004871962	NA	Down	ENSG00000196935	SRGAP1
ENST00000326505	4123	2.80102811	0.004880977	0.28738539	Ups	ENSG00000176597	B3GNT5
ENST00000233025	1274	-2.615389506	0.004936043	0.289416665	Down	ENSG00000114902	SPCS1
ENST00000553159	243	3.548460888	0.00497283	0.289871742	Ups		
ENST00000568588	1864	-4.07713097	0.004972965	NA	Down	ENSG00000174444	RPL4

ENST00000526027	1064	3.200390285	0.004985003	0.289871742	Ups	ENSG00000179532	DNHD1
ENST00000519167	793	-4.152822007	0.005033491	NA	Down	ENSG00000246366	RP11-382J12.1
ENST00000513610	8038	4.014924706	0.005045136	NA	Ups	ENSG00000145555	MYO10
ENST00000584164	873	3.582240769	0.005086865	0.29370663	Ups	ENSG00000161970	RPL26
ENST00000469270	747	2.65020362	0.005092696	0.29370663	Ups	ENSG00000073711	PPP2R3A
ENST00000366753	1368	-4.080522089	0.005094046	NA	Down	ENSG00000154342	WNT3A
ENST00000463873	448	1.822330091	0.005121615	0.294168813	Ups	ENSG00000241438	TDGF1P6
ENST00000393695	7343	-4.14531101	0.005124078	NA	Down	ENSG00000137497	NUMA1
ENST00000369905	2829	3.993150444	0.005149658	NA	Ups	ENSG00000138107	ACTR1A
ENST00000492582	2069	-4.071050376	0.005176937	NA	Down	ENSG00000100288	CHKB
ENST00000376436	3613	-4.141980734	0.005199914	NA	Down	ENSG00000130684	ZNF337
ENST00000396594	5298	1.139905516	0.005276438	0.30067037	Ups	ENSG00000159921	GNE
ENST00000314250	5010	4.12640637	0.00528565	0.30067037	Ups	ENSG00000111077	TENC1
ENST00000408038	5982	1.995616367	0.005298909	0.30067037	Ups	ENSG00000166167	BTRC
ENST00000381697	3290	2.323953059	0.005328341	0.301126184	Ups	ENSG00000214562	NUTM2D
ENST00000488195	868	3.996298415	0.005333772	NA	Ups	ENSG00000124571	XPO5
ENST00000564731	2800	2.267440603	0.005358951	0.301644608	Ups	ENSG00000140795	MYLK3
ENST00000542088	1040	4.046694447	0.005364236	NA	Ups	ENSG00000169718	DUS1L
ENST00000479834	681	-2.885968588	0.005389154	0.302084674	Down	ENSG00000135930	EIF4E2
ENST00000504656	1203	2.179357601	0.005409703	0.302084674	Ups	ENSG00000250848	CTD-2083E4.5
ENST00000467148	8064	-4.00394408	0.005433085	NA	Down	ENSG00000130589	HELZ2
ENST00000368648	4375	4.031164205	0.005468368	NA	Ups	ENSG00000108001	EBF3
ENST00000402367	1617	4.02874139	0.005514549	NA	Ups	ENSG00000170889	RPS9
ENST00000254231	3458	-3.655745864	0.005521477	0.30710761	Down	ENSG00000131914	LIN28A
ENST00000529150	575	2.739479241	0.005557435	0.307890634	Ups	ENSG00000175104	TRAF6
ENST00000407308	1978	3.968297971	0.005567484	NA	Ups	ENSG00000167065	DUSP18
ENST00000375918	922	-3.990141724	0.005611149	NA	Down	ENSG00000204444	APOM
ENST00000419996	922	-3.990141724	0.005611149	NA	Down		
ENST00000425856	922	-3.990141724	0.005611149	NA	Down		
ENST00000426401	922	-3.990141724	0.005611149	NA	Down		
ENST00000440956	922	-3.990141724	0.005611149	NA	Down		
ENST00000452949	922	-3.990141724	0.005611149	NA	Down		
ENST00000369496	3572	4.026504673	0.005612026	NA	Ups	ENSG00000163399	ATP1A1
ENST00000572400	4351	2.637713285	0.005639286	0.31120012	Ups	ENSG00000186260	MKL2
ENST00000295864	679	-4.105794411	0.005679866	NA	Down	ENSG00000163607	GTPBP8
ENST00000527937	4165	3.976555993	0.005735538	NA	Ups	ENSG00000166473	PKD1L2
ENST00000323061	2321	1.67832313	0.005753005	0.316235485	Ups	ENSG00000177432	NAP1L5

ENST00000423176	3073	3.987477295	0.005792999	NA	Ups	ENSG00000239900	ADSL
ENST00000413616	2232	2.732957555	0.005806361	0.317926522	Ups	ENSG00000229894	GK3P
ENST00000254579	14862	4.047666272	0.005820656	NA	Ups	ENSG00000179532	DNHD1
ENST00000473464	517	2.702162254	0.005863801	0.319827151	Ups	ENSG00000107262	BAG1
ENST00000513494	1577	3.972230497	0.005878571	NA	Ups	ENSG00000156219	ART3
ENST00000522308	1981	3.944110795	0.005902956	NA	Ups	ENSG00000172167	MTBP
ENST00000479918	1296	-4.09704174	0.005922203	0.321765434	Down	ENSG00000117500	TMED5
ENST00000578284	2632	3.992220951	0.005942431	NA	Ups		
ENST00000398667	2866	-4.012338479	0.005943065	NA	Down	ENSG00000205457	TP53TG3C
ENST00000247271	1947	-4.012385053	0.00597087	NA	Down	ENSG00000126861	OMG
ENST00000581692	4158	-3.554007196	0.005972614	0.323188946	Down	ENSG00000005379	BZRAP1
ENST00000264274	2540	-4.085158198	0.005974617	NA	Down	ENSG00000064012	CASP8
ENST00000376233	4053	-4.084388961	0.005987723	NA	Down	ENSG00000121406	ZNF549
ENST00000521889	603	2.59338243	0.005994337	0.323188946	Ups	ENSG00000213865	C8orf44
ENST00000458179	1537	-4.014107996	0.00600742	NA	Down		
ENST00000552279	830	1.77962665	0.006033488	0.324058182	Ups	ENSG00000139266	43533
ENST00000406041	1913	-1.965396	0.006064848	0.324503946	Down	ENSG00000170634	ACYP2
ENST00000489987	1433	4.014041301	0.006087117	NA	Ups	ENSG00000179348	GATA2
ENST00000359755	6190	-3.99857128	0.006105487	NA	Down	ENSG00000131016	AKAP12
ENST00000592148	3510	-2.304642907	0.006156956	0.327202687	Down	ENSG00000182534	MXRA7
ENST00000540651	1972	-4.083694317	0.00616179	0.327202687	Down	ENSG00000143761	ARF1
ENST00000381980	1943	1.629665474	0.006188455	0.327383255	Ups	ENSG00000154274	C4orf19
ENST00000527438	714	-4.060874971	0.00619636	NA	Down	ENSG00000185000	DGAT1
ENST00000396856	1266	3.976816773	0.006210242	NA	Ups	ENSG00000111640	GAPDH
ENST00000359883	1536	2.670563275	0.006253402	0.328052322	Ups	ENSG00000147853	AK3
ENST00000331437	4954	4.0680621	0.006255391	0.328052322	Ups	ENSG00000183307	CECR6
ENST00000441159	5568	1.48508665	0.00627104	0.328052322	Ups	ENSG00000170379	FAM115C
ENST00000443903	957	-3.368307395	0.006273614	NA	Down	ENSG00000204301	NOTCH4
ENST00000425778	2857	3.422390189	0.006275022	NA	Ups	ENSG00000115875	SRSF7
ENST00000579937	2798	-4.010204454	0.006285326	NA	Down	ENSG00000108262	GIT1
ENST00000393630	10382	3.98673858	0.006315466	NA	Ups	ENSG00000061987	MON2
ENST00000525600	512	-1.646832209	0.006360499	0.331499811	Down	ENSG00000172977	KAT5
ENST00000591340	4331	3.991106314	0.006384735	NA	Ups	ENSG00000186020	ZNF529
ENST00000509786	737	3.933701152	0.006418344	NA	Ups	ENSG00000248288	CTD-2194F4.2
ENST00000549333	395	-3.344175182	0.006476076	NA	Down	ENSG00000238168	RP11-686G8.1
ENST00000565570	1844	-1.920637395	0.006492129	0.335724229	Down	ENSG00000162066	AMDHD2
ENST00000265348	5254	-2.93835622	0.006532065	0.335724229	Down	ENSG00000044090	CUL7

ENST00000261479	1029	3.967501493	0.006542847	NA	Ups	ENSG00000100902	PSMA6
ENST00000505994	3505	2.315384889	0.006551347	0.335724229	Ups	ENSG00000249244	RP11-548H18.2
ENST00000571429	661	1.195509773	0.006558382	0.335724229	Ups	ENSG00000262480	SAMD11P1
ENST00000554961	814	4.033551055	0.00663893	0.335724229	Ups	ENSG00000100902	PSMA6
ENST00000588771	593	3.364831734	0.006650411	NA	Ups	ENSG00000134769	DTNA
ENST00000585971	550	2.409087998	0.006659234	0.335724229	Ups	ENSG00000153391	INO80C
ENST00000571647	2401	-2.306315246	0.006675453	0.335724229	Down	ENSG00000185359	HGS
ENST00000371828	3534	4.037404211	0.006697876	0.335724229	Ups	ENSG00000124214	STAU1
ENST00000520121	3785	-1.619978583	0.006702947	0.335724229	Down	ENSG00000164040	PGRMC2
ENST00000546587	597	3.968265809	0.006716016	NA	Ups	ENSG00000173588	CCDC41
ENST00000524463	6125	1.684640516	0.006733544	0.335724229	Ups	ENSG00000109861	CTSC
ENST00000262077	5487	2.032793012	0.006747394	0.335724229	Ups	ENSG00000124789	NUP153
ENST00000475000	2507	3.985018237	0.006750308	NA	Ups		
ENST00000378138	1815	3.225174916	0.00677495	0.335724229	Ups	ENSG00000171659	GPR34
ENST00000556645	2508	-2.115469302	0.006818973	0.335724229	Down	ENSG00000140105	WARS
ENST00000505479	631	3.497254024	0.006827311	0.335724229	Ups	ENSG00000138674	SEC31A
ENST00000594325	1020	-1.297345522	0.006832332	0.335724229	Down	ENSG00000006016	CRLF1
ENST00000475993	2860	-3.933846266	0.006836626	NA	Down	ENSG00000204574	ABCF1
ENST00000486105	2860	-3.933846266	0.006836626	NA	Down		
ENST00000494413	2860	-3.933846266	0.006836626	NA	Down		
ENST00000517412	865	-4.006965848	0.006840731	NA	Down	ENSG00000145675	PIK3R1
ENST00000450682	337	1.830916921	0.0068559	0.335724229	Ups	ENSG00000224370	RP11-814E24.3
ENST00000540827	6778	4.029420974	0.006869717	0.335724229	Ups	ENSG00000135679	MDM2
ENST00000491289	4380	-1.334811216	0.00687099	0.335724229	Down	ENSG00000148297	MED22
ENST00000305836	3399	3.940184869	0.006886183	NA	Ups	ENSG00000132256	TRIM5
ENST00000262613	1969	-4.024318879	0.006902066	NA	Down	ENSG00000109062	SLC9A3R1
ENST00000276833	2297	-3.934837838	0.006914443	NA	Down	ENSG00000147804	SLC39A4
ENST00000540689	3595	2.102454616	0.00697619	0.336542017	Ups	ENSG00000160961	ZNF333
ENST00000367485	4765	-4.014363662	0.006987104	NA	Down	ENSG00000116690	PRG4
ENST00000579942	3279	-4.014363662	0.006987104	NA	Down		
ENST00000375173	3076	-1.632018732	0.006995155	0.336542017	Down	ENSG00000136925	TSTD2
ENST00000372321	3970	1.706360917	0.007000187	0.336542017	Ups	ENSG00000188199	NUTM2B
ENST00000424878	3634	-2.938121125	0.007004404	0.336542017	Down	ENSG00000128284	APOL3
ENST00000427577	921	2.840404761	0.007007306	0.336542017	Ups	ENSG00000219201	RP11-181C21.4
ENST00000528780	730	-3.936332972	0.007043879	NA	Down	ENSG00000185885	IFITM1
ENST00000281141	1542	3.938387906	0.007054963	NA	Ups	ENSG00000151465	CDC123
ENST00000584437	5575	2.261497631	0.007088733	0.339294753	Ups	ENSG00000108375	RNF43

ENST00000537256	2191	-3.949889371	0.007091806	NA	Down	ENSG00000154118	JPH3
ENST00000357321	2102	-4.016760563	0.007158501	NA	Down	ENSG00000166321	NUDT13
ENST00000552743	606	3.914008263	0.007165067	NA	Ups	ENSG00000087470	DNM1L
ENST00000488019	565	2.213106698	0.007194413	0.343185686	Ups	ENSG00000244490	RWDD4P1
ENST00000466636	917	-3.943804537	0.007203691	NA	Down	ENSG00000144867	SRPRB
ENST00000598641	2652	-4.016708387	0.007252992	NA	Down		
ENST00000568232	958	-1.628733766	0.007304598	0.346567135	Down	ENSG00000179335	CLK3
ENST00000486028	719	2.32990962	0.007314557	0.346567135	Ups	ENSG00000064961	HMG20B
ENST00000529634	679	3.877319284	0.00736411	NA	Ups	ENSG00000010361	FUZ
ENST00000441788	5212	-1.79222148	0.007380966	0.347646722	Down	ENSG00000105325	FZR1
ENST00000409871	11540	-2.193487343	0.007386752	0.347646722	Down	ENSG00000188177	ZC3H6
ENST00000358752	6047	-2.594351171	0.007458916	0.349872907	Down	ENSG00000196371	FUT4
ENST00000482546	472	3.855321805	0.007478794	NA	Ups	ENSG00000066923	STAG3
ENST00000602389	3639	1.935871766	0.00751142	0.351165125	Ups	ENSG00000148484	RSU1
ENST00000514254	5040	-3.345674671	0.007552254	0.351905011	Down	ENSG00000250091	DNAH10OS
ENST00000343597	3488	-3.977192648	0.007568309	NA	Down	ENSG00000189057	FAM111B
ENST00000230990	2386	3.920161768	0.007573689	NA	Ups	ENSG00000113070	HBEGF
ENST00000502506	1166	-3.971438219	0.007644448	NA	Down	ENSG00000186010	NDUFA13
ENST00000370137	1396	3.847751227	0.007655203	NA	Ups	ENSG00000122477	LRRC39
ENST00000447022	2660	-3.974852796	0.007656936	NA	Down	ENSG00000176227	CTAGE15
ENST00000343546	2268	-3.96698793	0.0076885	NA	Down	ENSG00000178467	P4HTM
ENST00000508956	2422	3.912019056	0.007711835	NA	Ups	ENSG00000174611	KY
ENST00000599198	2956	3.180817656	0.007818016	NA	Ups	ENSG00000256612	CYP2B7P
ENST00000303908	930	-3.907959861	0.007830562	NA	Down	ENSG00000115762	PLEKHB2
ENST00000466296	3672	-3.650290482	0.007839907	0.362205858	Down		
ENST00000554577	5276	-3.162824557	0.007850539	0.362205858	Down	ENSG00000129467	ADCY4
ENST00000580545	3853	-3.970467753	0.007875058	NA	Down		
ENST00000366835	4098	-3.859742675	0.007878745	NA	Down	ENSG00000196187	TMEM63A
ENST00000529098	2532	-3.906291276	0.007933266	NA	Down	ENSG00000172367	PDZD3
ENST00000496037	542	2.112130959	0.007941163	0.363918755	Ups	ENSG00000138375	SMARCAL1
ENST00000525199	3442	3.899683127	0.007975535	NA	Ups	ENSG00000214534	ZNF705E
ENST00000452449	4535	2.827183273	0.00797631	NA	Ups	ENSG00000065060	UHRF1BP1
ENST00000535468	5504	3.950918773	0.007978265	0.363918755	Ups	ENSG00000044090	CUL7
ENST00000527881	3554	2.477552565	0.007979127	0.363918755	Ups	ENSG00000182704	TSKU
ENST00000339626	1680	1.596446264	0.008002358	0.363918755	Ups	ENSG00000229456	RLIMP1
ENST00000551132	618	2.294468341	0.008016971	0.363918755	Ups	ENSG00000111596	CNOT2
ENST00000567992	603	-3.911060264	0.008039816	NA	Down	ENSG00000179335	CLK3

ENST00000549536	240	2.621798159	0.008087705	0.365949147	Ups		
ENST00000525144	3808	3.312461203	0.008114591	0.365988866	Ups	ENSG00000149571	KIRREL3
ENST00000506451	425	-3.901502823	0.008133429	NA	Down	ENSG00000245958	RP11-33B1.1
ENST00000392779	3221	3.861385946	0.008179698	NA	Ups	ENSG00000169255	B3GALNT1
ENST00000609883	4105	-1.937440702	0.008246721	0.370759947	Down	ENSG00000242732	RGAG4
ENST00000373477	3238	-1.29393696	0.008289696	0.371505107	Down	ENSG00000134684	YARS
ENST00000544040	6514	-3.946747328	0.00832312	NA	Down	ENSG00000111642	CHD4
ENST00000504203	2243	-3.868802741	0.008354429	NA	Down	ENSG00000015479	MATR3
ENST00000428128	3479	3.886343427	0.008402101	NA	Ups	ENSG00000187627	RGPD1
ENST00000457633	942	-3.024074825	0.008425767	NA	Down	ENSG00000180228	PRKRA
ENST00000505341	1881	2.394583455	0.008438763	0.376651616	Ups	ENSG00000169609	C15orf40
ENST00000453471	620	3.852441957	0.008441802	NA	Ups	ENSG00000110717	NDUFS8
ENST00000498628	926	3.309808935	0.008467126	NA	Ups	ENSG00000147889	CDKN2A
ENST00000437866	864	-3.849523381	0.008480018	NA	Down		
ENST00000449535	942	2.761526429	0.008510596	0.377795295	Ups	ENSG00000235076	GAPDHP52
ENST00000589553	2389	3.919445305	0.00853622	NA	Ups	ENSG00000141524	TMC6
ENST00000321854	1806	-3.93762219	0.008555031	NA	Down	ENSG00000181513	ACBD4
ENST00000409969	1605	2.951715198	0.008579199	0.378816486	Ups	ENSG00000114978	MOB1A
ENST00000603878	563	1.781344497	0.00858744	0.378816486	Ups	ENSG00000271550	RP11-561N12.7
ENST00000485438	4030	-2.94141673	0.008604949	NA	Down	ENSG00000143641	GALNT2
ENST00000469326	1992	3.855929579	0.008632223	NA	Ups	ENSG00000123843	C4BPB
ENST00000267082	2867	3.910555524	0.008708348	NA	Ups	ENSG00000139626	ITGB7
ENST00000588173	2934	-1.572473018	0.00871337	0.383021327	Down	ENSG00000160888	IER2
ENST00000566471	607	-3.153157097	0.008737198	0.383021327	Down	ENSG00000149929	HIRIP3
ENST00000474967	2319	3.860812226	0.008763821	NA	Ups	ENSG00000122490	PQLC1
ENST00000314852	2028	-1.397244029	0.008788591	0.384077809	Down	ENSG00000177971	IMP3
ENST00000577197	650	-3.356660552	0.008791539	NA	Down	ENSG00000182173	TSEN54
ENST00000560984	4893	-3.919980554	0.008828185	NA	Down	ENSG00000205271	CSPG4P10
ENST00000507120	703	-3.917511875	0.008854499	NA	Down	ENSG00000087269	NOP14
ENST00000415440	2759	1.583095283	0.008864016	0.385126743	Ups	ENSG00000181896	ZNF101
ENST00000357246	1182	1.93992554	0.00886733	0.385126743	Ups	ENSG00000197769	MAP1LC3C
ENST00000484717	2072	-2.343138458	0.00894632	0.387361879	Down	ENSG00000136059	VILL
ENST00000444078	2527	-3.865819759	0.008957903	NA	Down	ENSG00000135898	GPR55
ENST00000421349	1589	-3.844632551	0.00896866	NA	Down		
ENST00000489188	784	-2.895345027	0.009033617	NA	Down	ENSG00000116754	SRSF11
ENST00000356554	8576	-2.750456192	0.009132641	0.393782144	Down	ENSG00000198162	MAN1A2
ENST00000523577	3003	2.493390816	0.00916576	0.393782144	Ups	ENSG00000188343	FAM92A1

ENST00000441174	4004	2.696601139	0.00918123	0.393782144	Ups	ENSG00000154144	TBRG1
ENST00000220659	2603	-2.499560427	0.009206532	0.393782144	Down	ENSG00000104221	BRF2
ENST00000513168	575	3.151858509	0.009208409	NA	Ups	ENSG00000249848	RP11-212F11.1
ENST00000367723	5914	-3.903190904	0.009212989	NA	Down	ENSG00000094975	SUCO
ENST00000356798	5213	-3.826077787	0.009235635	NA	Down	ENSG00000005844	ITGAL
ENST00000319084	1050	-3.907233327	0.009256236	NA	Down	ENSG00000204859	ZBTB48
ENST00000359203	4042	-3.796128345	0.009272482	NA	Down	ENSG00000025039	RRAGD
ENST00000394897	3337	-3.30973025	0.009279884	NA	Down	ENSG00000172955	ADH6
ENST00000578501	1124	1.947961967	0.009284443	0.395911149	Ups	ENSG00000141551	CSNK1D
ENST00000246222	1178	3.152048057	0.009332333	NA	Ups	ENSG00000125997	BPIFB9P
ENST00000482297	4616	-3.199015724	0.009372831	NA	Down		
ENST00000495807	4616	-3.199015724	0.009372831	NA	Down	ENSG00000204386	NEU1
ENST00000518586	2623	-3.888650283	0.009375376	NA	Down	ENSG00000147475	ERLIN2
ENST00000227520	1893	-3.861588858	0.009392462	NA	Down	ENSG00000110104	CCDC86
ENST00000486918	908	-3.883050619	0.009406953	NA	Down	ENSG00000162377	COA7
ENST00000368144	1172	-3.864159282	0.009437759	NA	Down	ENSG00000180433	OR6K6
ENST00000528957	1015	-3.813545737	0.009496226	NA	Down	ENSG00000161016	RPL8
ENST00000531525	1513	3.793074465	0.009521116	NA	Ups	ENSG00000149311	ATM
ENST00000477814	1784	-1.801217436	0.009588946	0.40228397	Down	ENSG00000100429	HDAC10
ENST00000439785	2651	3.877438425	0.009602719	NA	Ups	ENSG00000168661	ZNF30
ENST00000498649	1044	-3.376754525	0.009603694	NA	Down	ENSG00000099949	LZTR1
ENST00000603187	3713	3.876879563	0.0096101	0.40228397	Ups	ENSG00000214655	ZSWIM8
ENST00000369889	4104	-3.873410952	0.009662089	NA	Down	ENSG00000166272	WBP1L
ENST00000358065	2443	2.225858547	0.009679041	0.40228397	Ups	ENSG00000170364	SETMAR
ENST00000562192	313	-2.61148489	0.009689023	0.40228397	Down	ENSG00000138621	PPCDC
ENST00000311148	1713	-2.934747213	0.009697588	NA	Down	ENSG00000184227	ACOT1
ENST00000296411	2791	3.874346976	0.009707271	0.40228397	Ups	ENSG00000164024	METAP1
ENST00000354371	4007	-1.79828509	0.009713494	0.40228397	Down	ENSG00000198612	COPS8
ENST00000421582	1324	-3.446170216	0.009729607	NA	Down	ENSG00000159884	CCDC107
ENST00000522552	1683	-3.890553128	0.009734997	NA	Down	ENSG00000113558	SKP1
ENST00000502308	2455	3.816400549	0.009782413	NA	Ups	ENSG00000230219	FAM92A1P2
ENST00000442286	1496	-1.96981178	0.00979535	0.40228397	Down	ENSG00000125454	SLC25A19
ENST00000471850	1499	-3.81698455	0.009802816	NA	Down	ENSG00000144843	ADPRH
ENST00000453246	4275	3.29739201	0.009805522	0.40228397	Ups	ENSG00000179869	ABCA13
ENST00000482006	857	-3.505426964	0.009813901	NA	Down		
ENST00000334351	2416	3.176696837	0.009820928	0.40228397	Ups	ENSG00000189266	PNRC2
ENST00000369996	1124	-2.421289051	0.00984308	0.40228397	Down	ENSG00000243509	TNFRSF6B

ENST00000296051	4665	-2.813485384	0.009848033	0.40228397	Down	ENSG00000163755	HPS3
ENST00000375258	8203	1.501951947	0.009876408	0.40228397	Ups	ENSG00000123600	METTL8
ENST00000586215	756	-1.585459562	0.009913128	0.40228397	Down	ENSG00000099817	POLR2E
ENST00000260569	5343	1.379038335	0.009920873	0.40228397	Ups	ENSG00000011523	CEP68
ENST00000594827	1542	3.432587432	0.009923451	0.40228397	Ups	ENSG00000167757	KLK11
ENST00000395655	2225	-2.25966259	0.009942029	0.40228397	Down	ENSG00000136270	TBRG4
ENST00000578332	1058	-1.136078118	0.009947872	0.40228397	Down		
ENST00000562045	1743	-1.822866626	0.009948467	0.40228397	Down	ENSG00000127561	SYNGR3
ENST00000558373	4255	-3.860573014	0.009963355	NA	Down	ENSG00000137770	CTDSPL2
ENST00000471073	1809	-3.857762217	0.009995936	NA	Down	ENSG00000184436	THAP7
ENST00000450197	835	2.749082556	0.010053297	0.404192023	Ups	ENSG00000235060	VDAC1P4
ENST00000583277	1578	3.767916754	0.010055617	NA	Ups	ENSG00000161956	SENP3
ENST00000572562	1513	-3.809452968	0.010061011	NA	Down	ENSG00000141579	ZNF750
ENST00000422177	1785	2.963474939	0.010062123	0.404192023	Ups	ENSG00000128581	RABL5
ENST00000431365	580	-2.374787353	0.010081822	0.404192023	Down	ENSG00000171136	RLN3
ENST00000378536	5613	-1.788171177	0.010114732	0.404359412	Down	ENSG00000157933	SKI
ENST00000373064	3474	-3.854314082	0.010120582	NA	Down	ENSG00000148339	SLC25A25
ENST00000547140	604	-3.858662131	0.010125733	NA	Down	ENSG00000139318	DUSP6
ENST00000447225	1779	-3.85906126	0.010126495	NA	Down	ENSG00000174326	SLC16A11
ENST00000473942	2458	3.782085866	0.010132028	NA	Ups	ENSG00000170502	NUDT9
ENST00000295887	4461	3.423632536	0.010149105	0.404584151	Ups	ENSG00000163624	CDS1
ENST00000361710	3563	3.786057027	0.010194122	NA	Ups	ENSG00000128284	APOL3
ENST00000609841	6590	3.255334694	0.010194702	NA	Ups	ENSG00000215146	RP11-313J2.1
ENST00000490137	411	-3.806502291	0.010234741	NA	Down	ENSG00000072135	PTPN18
ENST00000397841	2303	-1.080143475	0.010244551	0.406768822	Down	ENSG00000183250	C21orf67
ENST00000533089	5900	3.750835619	0.0102669	NA	Ups	ENSG00000069702	TGFBR3
ENST00000541777	3743	1.647366302	0.01030491	0.406768822	Ups	ENSG00000167766	ZNF83
ENST00000577070	3927	1.370640148	0.010322936	0.406768822	Ups		
ENST00000415496	6407	-2.019940844	0.010332787	0.406768822	Down	ENSG00000102858	MGRN1
ENST00000430711	2323	1.616298031	0.01037567	0.406768822	Ups	ENSG00000215018	COL28A1
ENST00000498401	958	1.799114612	0.010377346	0.406768822	Ups	ENSG00000242488	RP11-270M14.1
ENST00000590834	2373	-3.796918007	0.010434498	NA	Down	ENSG00000108309	RUNDC3A
ENST00000479929	3140	1.127574504	0.010434668	0.407637636	Ups	ENSG00000100336	APOL4
ENST00000491061	3863	1.273948641	0.010459478	0.407637636	Ups	ENSG00000178234	GALNT11
ENST00000370535	1381	2.392387571	0.010491965	0.407637636	Ups	ENSG00000203930	LINC00632
ENST00000528876	509	-1.759023957	0.010515382	0.407637636	Down	ENSG00000255062	RP11-712L6.5
ENST00000458323	852	2.187346569	0.010615481	0.410155658	Ups	ENSG00000237679	VDAC1P11

ENST00000607267	9267	2.351801719	0.010642707	0.410155658	Ups		
ENST00000399603	7377	-3.839337985	0.010648463	NA	Down	ENSG00000151067	CACNA1C
ENST00000468812	2238	2.00831559	0.010667778	0.410155658	Ups	ENSG00000196262	PPIA
ENST00000276692	1018	3.839022266	0.010712695	0.410282576	Ups	ENSG00000147687	TATDN1
ENST00000254940	2402	3.75225223	0.010766704	NA	Ups	ENSG00000132603	NIP7
ENST00000412866	1474	-3.826177562	0.010767224	NA	Down	ENSG00000155659	VSIG4
ENST00000447279	1502	1.763041704	0.010769283	0.410282576	Ups	ENSG00000196345	ZKSCAN7
ENST00000380742	1388	3.758337677	0.010786653	NA	Ups	ENSG00000205571	SMN2
ENST00000507920	786	-3.82649845	0.010787252	NA	Down	ENSG00000250722	SEPP1
ENST00000304567	3271	2.482959765	0.010787703	0.410282576	Ups	ENSG00000171848	RRM2
ENST00000424254	1334	-3.81755803	0.010814397	NA	Down	ENSG00000143756	FBXO28
ENST00000309117	3256	3.824723014	0.010830551	NA	Ups	ENSG00000157538	DSCR3
ENST00000389722	10063	-1.25321414	0.010886908	0.412939527	Down	ENSG00000070182	SPTB
ENST00000457408	2322	3.82027669	0.010888847	NA	Ups	ENSG00000158604	TMED4
ENST00000514734	907	3.712679003	0.010931972	NA	Ups	ENSG00000164347	GFM2
ENST00000359594	1008	-3.162005841	0.01095458	NA	Down	ENSG00000196539	OR2T3
ENST00000409785	7568	2.14124581	0.010966591	0.414241436	Ups	ENSG00000176407	KCMF1
ENST00000519783	1953	3.405395162	0.010980106	0.414241436	Ups	ENSG00000145736	GTF2H2
ENST00000574857	3885	2.08546551	0.011068969	0.41566772	Ups	ENSG00000141258	SGSM2
ENST00000469689	1372	-1.298395316	0.011076989	0.41566772	Down	ENSG00000127838	PNKD
ENST00000578000	2458	3.733156468	0.011154109	NA	Ups	ENSG00000204650	CRHR1-IT1
ENST00000300584	6067	-3.748927391	0.011209536	NA	Down	ENSG00000167202	TBC1D2B
ENST00000496816	2222	-3.753719943	0.011238804	NA	Down	ENSG00000258366	RTEL1
ENST00000355690	1428	-3.806423797	0.011245798	NA	Down	ENSG00000172322	CLEC12A
ENST00000506997	1643	-3.806423797	0.011245798	NA	Down	ENSG00000272869	CTC-487M23.8
ENST00000540001	4713	-3.815380485	0.011291875	NA	Down	ENSG00000058804	NDC1
ENST00000608535	3915	3.693933937	0.011312407	NA	Ups	ENSG00000226524	RP1-102H19.8
ENST00000528089	2496	-1.712009576	0.011344636	0.421366716	Down	ENSG00000160172	FAM86C2P
ENST00000604008	393	-3.130539987	0.011351921	0.421366716	Down		
ENST00000534198	431	2.090754298	0.011356654	0.421366716	Ups	ENSG00000173914	RBM4B
ENST00000374007	3715	-2.534690664	0.011414268	0.421366716	Down	ENSG00000204147	ASAH2B
ENST00000332900	3317	3.736786339	0.011432705	NA	Ups	ENSG00000185332	TMEM105
ENST00000558065	3751	-1.614246437	0.011434393	0.421366716	Down	ENSG00000185033	SEMA4B
ENST00000586449	898	2.697451329	0.011451787	0.421366716	Ups	ENSG00000153391	INO80C
ENST00000458714	2285	-1.58000992	0.011455206	0.421366716	Down	ENSG00000176472	ZNF575
ENST00000400721	1568	-2.679200386	0.011468714	0.421366716	Down	ENSG00000141522	ARHGDIA
ENST00000563818	1931	1.829419788	0.011498353	0.421366716	Ups	ENSG00000154099	DNAAF1

ENST00000526764	516	3.685515737	0.011522943	NA	Ups	ENSG00000149571	KIRREL3
ENST00000337273	11183	-3.745322636	0.011535815	NA	Down	ENSG00000083312	TNPO1
ENST00000296387	2859	3.735458515	0.011560277	NA	Ups	ENSG00000164007	CLDN19
ENST00000548706	651	-1.417773982	0.011587419	0.421729349	Down	ENSG00000139626	ITGB7
ENST00000255320	642	2.453807888	0.011601852	0.421729349	Ups	ENSG00000132967	HMGB1P5
ENST00000558931	2754	-3.788127511	0.011602096	NA	Down	ENSG00000100911	PSME2
ENST00000466059	5750	3.677966824	0.011603	NA	Ups	ENSG00000144857	BOC
ENST00000511436	709	2.214470052	0.01162208	0.421729349	Ups	ENSG00000164211	STARD4
ENST00000216297	4684	-2.849212605	0.011629835	NA	Down	ENSG00000092201	SUPT16H
ENST00000372336	4933	1.717281226	0.011633677	0.421729349	Ups	ENSG00000165424	ZCCHC24
ENST00000495735	1632	3.780584427	0.011651012	NA	Ups	ENSG00000171307	ZDHHC16
ENST00000508192	2924	-3.732926749	0.011654286	NA	Down	ENSG00000072518	MARK2
ENST00000521046	856	-3.248580712	0.011654508	NA	Down	ENSG00000168300	PCMTD1
ENST00000525547	1697	2.389832492	0.011658095	0.421729349	Ups	ENSG00000150782	IL18
ENST00000527211	3679	-3.715674294	0.011658351	NA	Down	ENSG00000149091	DGKZ
ENST00000206595	5804	-3.10350787	0.011673783	NA	Down	ENSG00000092140	G2E3
ENST00000511311	668	1.935176661	0.011710167	0.422526859	Ups	ENSG00000186352	ANKRD37
ENST00000525899	2281	3.787196741	0.01172433	NA	Ups	ENSG00000164411	GJB7
ENST00000417263	507	1.331942472	0.011775592	0.423639951	Ups	ENSG00000237329	RP11-201O14.1
ENST00000596579	586	2.066980213	0.011801227	0.423639951	Ups	ENSG00000268784	MGC2752
ENST00000472442	2954	3.716719988	0.011829899	NA	Ups	ENSG00000227500	SCAMP4
ENST00000478092	718	-3.121257868	0.011838012	NA	Down	ENSG00000173175	ADCY5
ENST00000223210	6950	-1.166489559	0.011884072	0.424215696	Down	ENSG00000106479	ZNF862
ENST00000286298	8080	1.49662436	0.011884389	0.424215696	Ups	ENSG00000155850	SLC26A2
ENST00000416281	4493	-2.207354822	0.011907703	0.424215696	Down	ENSG00000173020	ADRBK1
ENST00000581139	1264	3.713848008	0.011927803	NA	Ups	ENSG00000263606	RP11-737O24.3
ENST00000373043	2229	-1.456224405	0.011951948	0.424267557	Down	ENSG00000197982	C1orf122
ENST00000520547	3832	1.722862023	0.012007131	0.424267557	Ups	ENSG00000253626	EIF5AL1
ENST00000567467	477	2.212077745	0.01202154	0.424267557	Ups	ENSG00000169752	NRG4
ENST00000233057	10042	1.340273507	0.012052974	0.424267557	Ups	ENSG00000055332	EIF2AK2
ENST00000323669	5503	-3.776253524	0.0120597	NA	Down	ENSG00000100523	DDHD1
ENST00000495707	4728	2.472971326	0.012095886	0.424267557	Ups	ENSG00000196584	XRCC2
ENST00000590794	489	-2.193500426	0.012148605	0.424267557	Down	ENSG00000117877	CD3EAP
ENST00000453873	4294	2.053999228	0.012162451	0.424267557	Ups	ENSG00000162929	KIAA1841
ENST00000539724	615	-1.781625482	0.012172464	0.424267557	Down	ENSG00000172725	CORO1B
ENST00000371790	4617	2.250551003	0.012208754	0.424267557	Ups	ENSG00000152779	SLC16A12
ENST00000521466	592	-2.457044718	0.012212025	0.424267557	Down	ENSG00000164587	RPS14

ENST00000369443	8828	3.717829255	0.012214037	NA	Ups	ENSG00000196505	GDAP2
ENST00000391437	2446	3.702112322	0.012222254	NA	Ups	ENSG00000212743	DKFZP667F0711
ENST00000578579	458	-2.342837835	0.012261443	0.424267557	Down	ENSG00000072778	ACADVL
ENST00000343625	3293	-1.845318674	0.012270956	0.424267557	Down	ENSG00000105122	RASAL3
ENST00000323830	3028	3.03056465	0.012361527	NA	Ups	ENSG00000178772	CPN2
ENST00000368805	10461	3.761977472	0.01239938	NA	Ups	ENSG00000009413	REV3L
ENST00000601297	1846	1.90022687	0.012400565	0.427697935	Ups	ENSG00000268211	AL359091.2
ENST00000572048	573	-2.531694263	0.012401986	NA	Down	ENSG00000132386	SERPINF1
ENST00000537760	2034	3.758268126	0.012405916	NA	Ups	ENSG00000133818	RRAS2
ENST00000565291	566	-3.718832237	0.012417786	NA	Down	ENSG00000137842	TMEM62
ENST00000298786	3614	3.760475798	0.012436662	NA	Ups	ENSG00000122376	FAM35A
ENST00000470151	701	-2.876992346	0.012522419	0.430553494	Down	ENSG00000082996	RNF13
ENST00000251527	5960	1.7445352	0.012544552	0.430553494	Ups	ENSG00000117868	ESYT2
ENST00000559404	1502	-3.698288869	0.012598245	NA	Down	ENSG00000156381	ANKRD9
ENST00000475829	1044	-3.717647076	0.012627159	NA	Down	ENSG00000138286	FAM149B1
ENST00000480319	3872	3.696623859	0.012631345	NA	Ups	ENSG00000183506	PI4KAP2
ENST00000562406	519	-3.750856668	0.012685532	NA	Down	ENSG00000172775	FAM192A
ENST00000483183	2441	3.751222599	0.012697072	NA	Ups		
ENST00000388950	3763	-3.696623724	0.0127558	NA	Down	ENSG00000204930	FAM221B
ENST00000496038	1871	-3.696623724	0.0127558	NA	Down	ENSG00000132692	BCAN
ENST00000389856	1641	-3.019613775	0.012768336	0.43697437	Down	ENSG00000015133	CCDC88C
ENST00000474162	1204	1.349485823	0.012793735	0.43697437	Ups	ENSG00000213891	RPL3P6
ENST00000414273	1543	-3.691971386	0.012881873	NA	Down	ENSG00000237973	hsa-mir-6723
ENST00000536186	1405	1.604476248	0.012910062	0.439357992	Ups	ENSG00000006837	CDKL3
ENST00000518152	784	2.135236359	0.012925967	0.439357992	Ups	ENSG00000246366	RP11-382J12.1
ENST00000320211	2590	-3.732959712	0.0129566	NA	Down	ENSG00000164053	ATRIP
ENST00000409040	962	3.673271092	0.012975735	NA	Ups	ENSG00000143924	EML4
ENST00000512215	4007	-3.697310892	0.013049163	NA	Down	ENSG00000138814	PPP3CA
ENST00000534834	4853	3.665056248	0.013061507	NA	Ups	ENSG00000198393	ZNF26
ENST00000239125	1469	-1.129440814	0.013064395	0.441946857	Down	ENSG00000120055	C10orf95
ENST00000505412	1903	1.697901814	0.013117202	0.441946857	Ups	ENSG00000168228	ZCCHC4
ENST00000350803	2146	-2.387421696	0.013123166	0.441946857	Down	ENSG00000115241	PPM1G
ENST00000598768	497	2.152879704	0.013134364	0.441946857	Ups	ENSG00000183207	RUVBL2
ENST00000469269	3467	2.840666094	0.013159162	0.441946857	Ups	ENSG00000198959	TGM2
ENST00000569450	513	2.772805273	0.013173975	NA	Ups	ENSG00000175318	GRAMD2
ENST00000509634	3340	-3.305158287	0.013190024	NA	Down	ENSG00000172262	ZNF131
ENST00000424649	3683	-3.005003039	0.013192986	0.442027859	Down	ENSG00000089159	PXN

ENST00000375544	2470	3.73870489	0.013205628	NA	Ups	ENSG00000106819	ASPN
ENST00000507719	1143	3.065969338	0.013207534	NA	Ups	ENSG00000109684	CLNK
ENST00000600988	2697	-3.684001801	0.013247486	NA	Down	ENSG00000167595	C19orf55
ENST00000382722	5475	-2.753277523	0.013297167	0.443200095	Down	ENSG00000151062	CACNA2D4
ENST00000296215	4563	1.273671399	0.013317724	0.443200095	Ups	ENSG00000163877	SNIP1
ENST00000309703	2337	2.921953764	0.013322459	0.443200095	Ups	ENSG00000138660	AP1AR
ENST00000562823	514	-2.390265099	0.013356281	0.443277331	Down	ENSG00000159322	ADPGK
ENST00000467354	2699	3.714759385	0.013402052	NA	Ups	ENSG00000159496	RGL4
ENST00000576104	565	-3.720112369	0.013444103	NA	Down	ENSG00000140993	TIGD7
ENST00000606895	2068	-1.812960825	0.013595021	0.449288725	Down	ENSG00000178607	ERN1
ENST00000361284	942	-3.726014431	0.013603924	NA	Down	ENSG00000198967	OR10Z1
ENST00000398246	3594	-1.586510916	0.013660223	0.449288725	Down	ENSG00000154359	LONRF1
ENST00000475941	2669	-1.056374297	0.013662506	0.449288725	Down	ENSG00000128191	DGCR8
ENST00000531486	2290	1.434347887	0.01366512	0.449288725	Ups	ENSG00000141279	NPEPPS
ENST00000577368	753	-3.107858699	0.013769545	NA	Down	ENSG00000008283	CYB561
ENST00000421974	2605	-1.754881299	0.013775231	0.451853251	Down	ENSG00000171130	ATP6V0E2
ENST00000453057	1525	-3.713441332	0.013851264	NA	Down		
ENST00000581396	5592	1.817662853	0.013989786	0.455611095	Ups	ENSG00000175662	TOM1L2
ENST00000515035	5240	1.219947708	0.014034204	0.455611095	Ups	ENSG00000070814	TCOF1
ENST00000370661	4363	1.67033974	0.014051311	0.455611095	Ups	ENSG00000129680	MAP7D3
ENST00000285176	8682	2.133691157	0.01406359	0.455611095	Ups	ENSG00000154874	CCDC144B
ENST00000268070	6207	3.60071603	0.014065097	NA	Ups	ENSG00000140470	ADAMTS17
ENST00000381433	1219	-3.648517551	0.014088594	NA	Down	ENSG00000128285	MCHR1
ENST00000434232	732	1.793582893	0.014090101	0.455611095	Ups	ENSG00000225739	NPM1P18
ENST00000431473	8909	1.718829703	0.014093444	0.455611095	Ups	ENSG00000177181	RIMKLA
ENST00000456542	580	3.018046274	0.014096901	NA	Ups	ENSG00000079263	SP140
ENST00000455730	1119	1.819746211	0.014119742	0.455611095	Ups	ENSG00000232662	LDHBP1
ENST00000291825	5502	3.702352293	0.014166721	NA	Ups	ENSG00000105227	PRX
ENST00000552527	5815	2.017138445	0.014174807	0.455611095	Ups	ENSG00000111300	NAA25
ENST00000281453	1998	-2.157562939	0.014181187	0.455611095	Down	ENSG00000151725	CENPU
ENST00000393179	3096	-2.16385306	0.014258118	0.456565735	Down	ENSG00000139323	POC1B
ENST00000595077	2465	3.687350325	0.014258259	NA	Ups	ENSG00000006015	C19orf60
ENST00000341785	2909	-3.655426191	0.014303271	NA	Down	ENSG00000143340	FAM163A
ENST00000479806	1798	1.898302561	0.014340775	0.456565735	Ups	ENSG00000077380	DYNC1I2
ENST00000367565	2500	-1.465099832	0.014368153	0.456565735	Down	ENSG00000203730	TEDDM1
ENST00000600820	3602	1.599463173	0.014414757	0.456565735	Ups	ENSG00000269657	AC079210.1
ENST00000373245	2339	-3.695613048	0.014415174	NA	Down	ENSG00000136877	FPGS

ENST00000421213	323	-2.380765156	0.014423095	0.456565735	Down		ENSG00000100218	RTDR1
ENST00000462250	514	-1.428631533	0.01443087	0.456565735	Down		ENSG00000125459	MSTO1
ENST00000436937	465	1.91587733	0.014438015	0.456565735	Ups		ENSG00000227073	SDHDP2
ENST00000502972	1916	3.590386192	0.014484652	NA	Ups		ENSG00000109180	OCIAD1
ENST00000543586	1638	2.978285806	0.014581345	NA	Ups		ENSG00000116525	TRIM62
ENST00000556765	664	-3.645571384	0.014584101	NA	Down		ENSG00000182400	TRAPPC6B
ENST00000397176	3042	3.245933418	0.014646448	0.461861267	Ups		ENSG00000065675	PRKCQ
ENST00000366356	249	2.779150638	0.014671119	0.461861267	Ups			
ENST00000361789	1141	-1.049865687	0.014749683	0.463015498	Down		ENSG00000198727	MT-CYB
ENST00000467544	658	3.639866408	0.014789838	NA	Ups		ENSG00000120438	TCP1
ENST00000431030	672	-3.621251187	0.014798137	NA	Down		ENSG00000235433	AC009960.4
ENST00000563845	528	-3.621251187	0.014798137	NA	Down		ENSG00000104731	KLHDC4
ENST00000600428	2249	-3.621251187	0.014798137	NA	Down		ENSG00000125733	TRIP10
ENST00000355727	1875	3.390023689	0.014812907	0.463015498	Ups		ENSG00000186063	AIDA
ENST00000592554	2955	2.455625928	0.014828345	0.463015498	Ups		ENSG00000108950	FAM20A
ENST00000333412	5177	-3.677401729	0.014849218	NA	Down		ENSG00000238083	LRRC37A2
ENST00000497675	4478	-1.540816772	0.014852859	0.463015498	Down		ENSG00000162585	C1orf86
ENST00000375441	2502	-1.40839749	0.0148723	0.463015498	Down		ENSG00000139842	CUL4A
ENST00000582010	1771	3.17691021	0.014887543	NA	Ups			
ENST00000372824	2223	2.608905166	0.014955821	0.464567143	Ups		ENSG00000101104	PABPC1L
ENST00000434712	514	2.340879647	0.014991914	0.464567143	Ups		ENSG00000229339	RP11-193I22.2
ENST00000549478	1446	3.614750313	0.015018088	NA	Ups		ENSG00000204954	C12orf73
ENST00000260270	3206	1.12489449	0.01502118	0.464567143	Ups		ENSG00000137714	FDX1
ENST00000589168	2125	-3.679822238	0.015104092	NA	Down		ENSG00000108639	SYNGR2
ENST00000547011	195	2.424877746	0.015106662	0.465782045	Ups			
ENST00000584516	5709	-3.013236594	0.015126662	0.465782045	Down		ENSG00000266714	MYO15B
ENST00000475072	658	3.58718909	0.015155851	NA	Ups		ENSG00000159140	SON
ENST00000541068	594	-3.670880337	0.015203185	NA	Down		ENSG00000123636	BAZ2B
ENST00000573347	810	-3.670880337	0.015203185	NA	Down		ENSG00000108798	ABI3
ENST00000533285	878	2.362510251	0.015213728	0.467440145	Ups		ENSG00000149269	PAK1
ENST00000382349	8318	1.895692822	0.015288195	0.468421151	Ups		ENSG00000205922	ONECUT3
ENST00000497427	570	3.618515516	0.015293267	NA	Ups		ENSG00000071794	HLTF
ENST00000409085	11345	3.596505727	0.015308034	NA	Ups		ENSG00000115977	AAK1
ENST00000552245	1812	2.625499832	0.015312232	0.468421151	Ups		ENSG00000185046	ANKS1B
ENST00000355716	1707	-1.723895359	0.015350741	0.468580546	Down		ENSG00000157873	TNFRSF14
ENST00000558504	522	2.542870858	0.015396829	0.468970074	Ups		ENSG00000128918	ALDH1A2
ENST00000503699	720	3.05560473	0.015450303	NA	Ups		ENSG00000131127	ZNF141

ENST00000608849	720	3.068813181	0.015472059	NA	Ups		
ENST00000233575	2044	-3.655066816	0.015484295	NA	Down	ENSG00000115234	SNX17
ENST00000375377	9265	-2.659557683	0.015500898	0.470422799	Down	ENSG00000165757	KIAA1462
ENST00000515800	4979	1.118926443	0.01551634	0.470422799	Ups	ENSG00000145715	RASA1
ENST00000398587	3936	3.590802484	0.01553023	NA	Ups	ENSG00000253873	PCDHGA11
ENST00000494484	2149	2.171836611	0.015544813	0.470422799	Ups	ENSG00000196262	PPIA
ENST00000506763	3166	-3.6129081	0.015592561	NA	Down	ENSG00000214367	HAUS3
ENST00000436527	1197	-3.64861962	0.015606391	NA	Down	ENSG00000197471	SPN
ENST00000340681	1110	3.580280398	0.01564142	NA	Ups	ENSG00000203262	RP5-961K14.2
ENST00000519718	483	-2.581895826	0.01567644	NA	Down	ENSG00000272772	CTD-2410N18.5
ENST00000443381	2267	-1.532508764	0.015753633	0.472286473	Down	ENSG00000158805	ZNF276
ENST00000403487	861	3.584308653	0.015757968	NA	Ups	ENSG00000217896	ZNF839P1
ENST00000510476	773	3.584308653	0.015757968	NA	Ups	ENSG00000121067	SPOP
ENST00000370766	5592	2.366099902	0.015784155	0.472286473	Ups	ENSG00000186376	ZNF75D
ENST00000593516	5592	2.366099902	0.015784155	0.472286473	Ups		
ENST00000287594	5820	1.065266288	0.015807549	0.472286473	Ups	ENSG00000156968	MPV17L
ENST00000368613	2113	-3.594660359	0.015966489	NA	Down	ENSG00000148814	LRRC27
ENST00000473155	753	-2.276987203	0.015975542	0.472286473	Down	ENSG00000168209	DDIT4
ENST00000381724	976	3.64017184	0.015990339	NA	Ups	ENSG00000088832	FKBP1A
ENST00000324198	9103	1.527010302	0.016005234	0.472286473	Ups	ENSG00000100077	ADRBK2
ENST00000380814	2121	-3.572263567	0.016008926	NA	Down	ENSG00000139508	SLC46A3
ENST00000469754	1034	3.411531136	0.016010285	0.472286473	Ups	ENSG00000176124	DLEU1
ENST00000206423	4664	2.374961932	0.016073685	0.472286473	Ups	ENSG00000091986	CCDC80
ENST00000378910	4276	2.13408995	0.01608595	0.472286473	Ups	ENSG00000161270	NPHS1
ENST00000439942	657	3.627224968	0.016090548	NA	Ups	ENSG00000235816	PRELID1P3
ENST00000558762	11726	3.571851469	0.016102651	NA	Ups	ENSG00000140443	IGF1R
ENST00000560255	2082	-1.12122288	0.016113067	0.472286473	Down	ENSG00000259642	C15orf37
ENST00000530187	621	-1.740204112	0.016133626	0.472286473	Down	ENSG00000173599	PC
ENST00000566051	791	2.597263272	0.016136144	0.472286473	Ups	ENSG00000132604	TERF2
ENST00000330387	7412	-3.24122156	0.01616415	NA	Down	ENSG00000182158	CREB3L2
ENST00000492902	1327	-1.559608434	0.016164483	0.472286473	Down	ENSG00000059915	PSD
ENST00000429216	963	1.585988312	0.016185681	0.472286473	Ups	ENSG00000225695	HNRNPA1P35
ENST00000541549	4486	-3.639097822	0.016219462	NA	Down	ENSG00000107581	EIF3A
ENST00000534880	579	2.549713255	0.016226594	0.472286473	Ups	ENSG00000174038	C9orf131
ENST00000284061	7768	2.072312193	0.016231504	0.472286473	Ups	ENSG00000153933	DGKE
ENST00000393038	901	-3.598814097	0.016232352	NA	Down	ENSG00000141378	PTRH2
ENST00000371222	3321	-1.616152871	0.016241174	0.472286473	Down	ENSG00000177606	JUN

ENST00000511697	558	3.543214276	0.016258528	NA	Ups	ENSG00000136436	CALCOCO2
ENST00000428321	991	-3.033884196	0.016264166	0.472286473	Down		
ENST00000491297	4206	3.536808808	0.016286113	NA	Ups	ENSG00000152763	WDR78
ENST00000468814	4181	-1.975314521	0.016296857	0.472286473	Down		
ENST00000547586	556	2.986051917	0.016338725	0.472286473	Ups	ENSG00000139531	SUOX
ENST00000358181	7286	-3.586127341	0.016379332	NA	Down	ENSG00000170004	CHD3
ENST00000357825	8406	2.18859799	0.01638891	0.472286473	Ups	ENSG00000196935	SRGAP1
ENST00000466801	784	-2.589533041	0.016401606	0.472286473	Down	ENSG00000187514	PTMA
ENST00000474077	605	-2.0277079	0.016405236	0.472286473	Down	ENSG00000144635	DYNC1LI1
ENST00000504091	4522	1.51556564	0.016411888	0.472286473	Ups	ENSG00000169247	SH3TC2
ENST00000328913	4980	3.61932296	0.016431175	NA	Ups	ENSG00000053524	MCF2L2
ENST00000492875	3894	3.576563412	0.016473492	NA	Ups	ENSG00000106948	AKNA
ENST00000375123	3451	2.113738564	0.016483533	0.473380162	Ups	ENSG00000178919	FOXE1
ENST00000595384	785	3.536379349	0.016486032	NA	Ups	ENSG00000105738	SIPA1L3
ENST00000367097	11123	2.709506378	0.016530116	NA	Ups	ENSG00000130338	TULP4
ENST00000537986	496	-3.013062217	0.016577567	NA	Down	ENSG00000196118	C16orf93
ENST00000567951	2918	-3.581596721	0.016613839	NA	Down	ENSG00000090863	GLG1
ENST00000425155	2329	-3.586229417	0.016649004	NA	Down	ENSG00000169220	RGS14
ENST00000536234	1748	-1.633925875	0.016700214	0.477970935	Down	ENSG00000149743	TRPT1
ENST00000521972	862	1.610765868	0.01671132	0.477970935	Ups	ENSG00000035681	NSMAF
ENST00000366211	1339	3.237610426	0.016754514	NA	Ups	ENSG00000157895	C12orf43
ENST00000534108	1540	-2.964487365	0.016760428	NA	Down	ENSG00000197165	SULT1A2
ENST00000315614	5182	-3.615117014	0.016762286	NA	Down	ENSG00000215041	NEURL4
ENST00000423778	2345	-3.615117014	0.016762286	NA	Down	ENSG00000174611	KY
ENST00000295984	3368	-2.233859205	0.0167819	0.478283393	Down	ENSG00000163704	PRRT3
ENST00000555188	6855	2.047914207	0.016790221	0.478283393	Ups	ENSG00000169291	SHE
ENST00000587100	768	-1.47755837	0.016842596	0.47850743	Down	ENSG00000183401	CCDC159
ENST00000585830	1117	-3.399230159	0.016866095	0.47850743	Down	ENSG00000006075	CCL3
ENST00000556885	1086	-3.614706664	0.016883533	NA	Down	ENSG00000205707	LYRM5
ENST00000533902	2043	-3.577473183	0.016908046	NA	Down	ENSG00000150687	PRSS23
ENST00000274068	3203	2.250292632	0.016948774	0.478921971	Ups	ENSG00000145428	RNF175
ENST00000503694	3203	2.250292632	0.016948774	0.478921971	Ups	ENSG00000145428	RNF175
ENST00000264335	2211	-3.613560307	0.017002521	NA	Down	ENSG00000108953	YWHAE
ENST00000343735	3431	-3.613560307	0.017002521	NA	Down	ENSG00000066405	CLDN18
ENST00000453146	2821	-3.607615458	0.017101912	NA	Down	ENSG00000130695	CEP85
ENST00000331615	3267	1.677040476	0.017145299	0.481675549	Ups	ENSG00000184988	TMEM106A
ENST00000476129	565	-3.538670203	0.017147539	NA	Down	ENSG00000163959	SLC51A

ENST00000282077	14193	2.493018616	0.017148395	0.481675549	Ups	ENSG00000152256	PDK1
ENST00000407029	4091	-1.632932479	0.017148909	0.481675549	Down	ENSG00000196588	MKL1
ENST00000472240	852	3.149264494	0.017156296	NA	Ups	ENSG00000198125	MB
ENST00000339526	3942	-3.568531355	0.017167512	NA	Down	ENSG00000135821	GLUL
ENST00000592360	2247	3.509453967	0.017207982	NA	Ups	ENSG00000256294	ZNF225
ENST00000444782	2154	-2.874776389	0.017266929	0.483615186	Down	ENSG00000164620	RELL2
ENST00000382764	878	3.552225506	0.0172711	NA	Ups	ENSG00000183146	CYorf17
ENST00000489654	875	3.552225506	0.0172711	NA	Ups	ENSG00000197448	GSTK1
ENST00000427638	1841	1.42118506	0.0172867	0.483615186	Ups	ENSG00000223760	MED15P9
ENST00000476499	1612	2.656319794	0.017296966	NA	Ups	ENSG00000107863	ARHGAP21
ENST00000437723	750	-3.542974	0.017318932	NA	Down	ENSG00000249240	AC069368.3
ENST00000542975	577	-3.564519498	0.017350643	NA	Down	ENSG00000109920	FNBP4
ENST00000299565	3623	2.387582213	0.017380508	0.485274827	Ups	ENSG00000169684	CHRNA5
ENST00000547459	3320	-3.600014685	0.017417337	NA	Down	ENSG00000151239	TWF1
ENST00000361487	4514	-3.384105385	0.017476447	NA	Down	ENSG00000198807	PAX9
ENST00000460536	4286	-1.399232545	0.017476838	0.486998156	Down	ENSG00000198198	SZT2
ENST00000505503	583	-3.596315558	0.017509043	NA	Down	ENSG00000171161	ZNF672
ENST00000395053	1733	-1.919289281	0.01754651	0.487612111	Down	ENSG00000171703	TCEA2
ENST00000443589	383	2.190451606	0.017568174	0.487612111	Ups	ENSG00000230683	DDTP1
ENST00000368563	3198	-3.54504735	0.017662996	NA	Down	ENSG00000130640	TUBGCP2
ENST00000522223	1538	-2.727988891	0.017667952	0.489107312	Down	ENSG00000105808	RASA4
ENST00000361582	5350	2.509928268	0.017691559	0.489107312	Ups	ENSG00000198919	DZIP3
ENST00000513601	1429	3.558379555	0.017708235	NA	Ups	ENSG00000169609	C15orf40
ENST00000600633	1329	-3.587281835	0.01771081	NA	Down	ENSG00000140264	SERF2
ENST00000412509	1558	3.118059017	0.017746331	NA	Ups	ENSG00000236988	RP11-402K9.1
ENST00000233202	3852	-1.632593143	0.017800594	0.491156795	Down	ENSG00000018280	SLC11A1
ENST00000466325	828	-3.518959493	0.017858025	NA	Down	ENSG00000196924	FLNA
ENST00000598290	828	-3.518959493	0.017858025	NA	Down		
ENST00000585386	1500	3.580572058	0.017914955	NA	Ups	ENSG00000125746	EML2
ENST00000398155	3998	-3.579076736	0.017921583	NA	Down	ENSG00000086712	TXLNG
ENST00000484707	545	3.492464552	0.017936109	NA	Ups	ENSG00000198466	ZNF587
ENST00000584783	668	3.492464552	0.017936109	NA	Ups	ENSG00000266605	LONRF2P1
ENST00000264741	7889	-3.551699294	0.017952922	NA	Down	ENSG00000144668	ITGA9
ENST00000468183	2432	-3.582687461	0.017965688	NA	Down	ENSG00000117410	ATP6V0B
ENST00000310585	3677	3.550009065	0.018003476	NA	Ups	ENSG00000175182	FAM131A
ENST00000339235	2111	-2.01278656	0.018103271	0.498530793	Down	ENSG00000171823	FBXL14
ENST00000422915	578	2.379616732	0.01812641	NA	Ups	ENSG00000231369	RP1-40G4P.1

ENST00000397439	1996	-3.538092443	0.018128314	NA	Down	ENSG00000187912	CLEC17A
ENST00000579446	452	-3.009501021	0.018144986	NA	Down	ENSG00000130255	RPL36
ENST00000354506	2415	-3.576053684	0.018209966	NA	Down	ENSG00000197713	RPE
ENST00000257604	3178	3.154507679	0.018212978	NA	Ups	ENSG00000135148	TRAFD1
ENST00000297579	2642	-1.019589295	0.018225353	0.499693052	Down	ENSG00000164934	DCAF13
ENST00000368863	6054	-1.714801257	0.018263996	0.499693052	Down	ENSG00000143442	POGZ
ENST00000529272	1311	-2.93349939	0.018274402	0.499693052	Down	ENSG00000104529	EEF1D
ENST00000399781	999	-1.497909559	0.018304313	0.499693052	Down	ENSG00000099968	BCL2L13
ENST00000550534	1669	1.59689706	0.018323026	0.499693052	Ups	ENSG00000257608	GRAMD4P3
ENST00000553000	214	3.517610204	0.018342546	NA	Ups	ENSG00000257979	SNRPGP18
ENST00000597537	583	3.521742766	0.018365394	NA	Ups	ENSG00000196081	ZNF724P
ENST00000469481	989	1.602775664	0.018443227	0.501475874	Ups	ENSG00000101972	STAG2
ENST00000468281	468	3.479269566	0.018485181	NA	Ups	ENSG00000224438	RP4-816N1.1
ENST00000572841	2450	1.051363068	0.018504031	0.501475874	Ups	ENSG00000141258	SGSM2
ENST00000378024	18787	-1.292337941	0.018516572	0.501475874	Down	ENSG00000124942	AHNAK
ENST00000449538	715	-3.536039683	0.018524172	NA	Down	ENSG00000233822	HIST1H2BN
ENST00000366292	1604	1.622543004	0.018556921	0.501475874	Ups	ENSG00000139370	SLC15A4
ENST00000439308	6053	-3.324291158	0.018566581	0.501475874	Down	ENSG00000128159	TUBGCP6
ENST00000355439	2078	-1.285677601	0.018612638	0.501725884	Down	ENSG00000215790	SLC35E2
ENST00000545225	3082	3.521060731	0.018642206	NA	Ups	ENSG00000131467	PSME3
ENST00000567523	1839	1.778200981	0.018647146	0.501725884	Ups	ENSG00000104164	BLOC1S6
ENST00000530371	585	-3.560407079	0.018786588	NA	Down	ENSG00000166250	CLMP
ENST00000398307	1390	-3.564928381	0.018788363	NA	Down	ENSG00000102539	MLNR
ENST00000420536	711	2.486732617	0.018847851	0.506158319	Ups	ENSG00000100316	RPL3
ENST00000504529	210	3.464974705	0.018868415	NA	Ups	ENSG00000248532	RP11-241F15.5
ENST00000447966	3778	-2.87498479	0.01890026	0.506598976	Down	ENSG00000110881	ASIC1
ENST00000338368	13413	2.347927819	0.018957393	0.507164311	Ups	ENSG00000253729	PRKDC
ENST00000369305	4241	-2.792991344	0.019032606	NA	Down	ENSG00000198924	DCLRE1A
ENST00000523009	453	2.027108094	0.019067231	0.508170603	Ups	ENSG00000254274	TDGF1P5
ENST00000456435	4704	-3.548729237	0.019117055	NA	Down	ENSG00000143624	INTS3
ENST00000475850	1965	1.63972529	0.019122912	0.50869114	Ups	ENSG00000186654	PRR5
ENST00000494978	2517	3.553539651	0.019131432	NA	Ups	ENSG00000253882	RP11-61L23.2
ENST00000527985	2817	-3.55411638	0.019143213	NA	Down	ENSG00000156599	ZDHHC5
ENST00000326873	3328	-2.146220383	0.019181894	0.508703619	Down	ENSG00000118046	STK11
ENST00000366131	553	2.297939067	0.019195681	0.508703619	Ups	ENSG00000111319	SCNN1A
ENST00000412097	562	-3.559705174	0.019207449	NA	Down	ENSG00000174748	RPL15
ENST00000423108	756	3.465072629	0.019262308	NA	Ups	ENSG00000156990	RPUSD3

ENST00000561095	2367	3.465072629	0.019262308	NA	Ups	ENSG00000140534	TICRR
ENST00000397088	2639	3.094002224	0.019282337	NA	Ups	ENSG00000164850	GPER1
ENST00000356180	1400	2.945580808	0.019319153	NA	Ups	ENSG00000129480	DTD2
ENST00000304748	1930	3.482745804	0.019342522	NA	Ups	ENSG00000171051	FPR1
ENST00000591741	1979	3.498939604	0.01937704	NA	Ups	ENSG00000130377	ACSBG2
ENST00000332585	4340	-3.498588959	0.019377239	NA	Down	ENSG00000184792	OSBP2
ENST00000369622	3023	-3.508912229	0.019385134	NA	Down	ENSG00000135316	SYNCRIP
ENST00000579645	1906	-2.312323014	0.019439609	0.514199589	Down	ENSG00000124422	USP22
ENST00000531504	591	-3.496574342	0.019463652	NA	Down	ENSG00000110422	HIPK3
ENST00000459645	477	3.460490006	0.019564797	NA	Ups	ENSG00000183054	RGPD6
ENST00000369681	9252	1.355207622	0.019570741	0.516696938	Ups	ENSG00000065615	CYB5R4
ENST00000392407	3645	3.106588246	0.019694294	NA	Ups	ENSG00000145113	MUC4
ENST00000266517	7159	-1.709898746	0.019698647	0.519099922	Down	ENSG00000139163	ETNK1
ENST00000462860	2376	-3.49822891	0.019800324	NA	Down	ENSG00000234769	WASH4P
ENST00000519697	1972	3.484416601	0.019829638	NA	Ups	ENSG00000164764	SBSPON
ENST00000461804	1998	-1.441286571	0.019865685	0.522115443	Down	ENSG00000163660	CCNL1
ENST00000558910	782	1.8871903	0.019887285	0.522115443	Ups	ENSG00000140416	TPM1
ENST00000310046	1840	1.155775092	0.020026242	0.524058147	Ups	ENSG00000173914	RBM4B
ENST00000443193	2301	3.526418089	0.020028602	NA	Ups	ENSG00000203747	FCGR3A
ENST00000475776	2178	3.526418089	0.020028602	NA	Ups	ENSG00000160298	C21orf58
ENST00000533277	2719	-1.198771271	0.020053787	0.524058147	Down	ENSG00000173653	RCE1
ENST00000536255	1057	-3.485830649	0.020058731	NA	Down	ENSG00000129197	RPAIN
ENST00000252137	5392	2.562455928	0.020073006	0.524058147	Ups	ENSG00000100056	DGCR14
ENST00000360009	3245	3.523088134	0.020107725	NA	Ups	ENSG00000117682	DHDDS
ENST00000469865	504	2.880836868	0.020123608	NA	Ups	ENSG00000133026	MYH10
ENST00000476737	592	3.476163357	0.0202006	NA	Ups	ENSG00000133026	MYH10
ENST00000506177	557	2.060585182	0.020264513	0.524786847	Ups	ENSG00000162571	TTLL10
ENST00000570688	579	-3.000765179	0.02028849	NA	Down	ENSG00000159842	ABR
ENST00000361673	7303	-2.419815533	0.020300573	0.524786847	Down	ENSG00000198796	ALPK2
ENST00000459880	599	3.443337415	0.020302213	NA	Ups	ENSG00000128609	NDUFA5
ENST00000442003	6797	-1.477335653	0.020320501	0.524786847	Down	ENSG00000137449	CPEB2
ENST00000415080	6026	-2.65302084	0.020332071	NA	Down	ENSG00000115970	THADA
ENST00000573102	1420	-3.53761443	0.020335023	NA	Down		
ENST00000491856	2578	3.467654644	0.020336144	NA	Ups	ENSG00000106069	CHN2
ENST00000292530	4672	2.795144581	0.020337541	0.524786847	Ups	ENSG00000160961	ZNF333
ENST00000592262	1978	2.218915224	0.020357992	0.524786847	Ups	ENSG00000076650	GPATCH1
ENST00000367690	1808	1.09402042	0.020368507	0.524786847	Ups	ENSG00000152061	RABGAP1L

ENST00000430983	3425	-1.996258599	0.020397179	0.524786847	Down	ENSG00000214174	AMZ2P1
ENST00000361681	525	1.429202385	0.020399261	0.524786847	Ups	ENSG00000198695	MT-ND6
ENST00000482481	8480	3.469529111	0.020463472	NA	Ups	ENSG00000137962	ARHGAP29
ENST00000395810	3235	1.806218548	0.020487354	0.525740751	Ups	ENSG00000198915	RASGEF1A
ENST00000399908	5595	1.557513328	0.020511063	0.525740751	Ups	ENSG00000008735	MAPK8IP2
ENST00000537671	395	-3.469759271	0.020512633	NA	Down	ENSG00000174038	C9orf131
ENST00000254616	2840	2.869011537	0.020534545	NA	Ups	ENSG00000132286	TIMM10B
ENST00000556921	893	-3.15409281	0.02054956	NA	Down	ENSG00000196405	EVL
ENST00000359576	7254	-3.47879718	0.020569561	NA	Down	ENSG00000163539	CLASP2
ENST00000397043	3197	-3.47879718	0.020569561	NA	Down	ENSG00000074370	ATP2A3
ENST00000240851	1978	-3.518575723	0.02058263	NA	Down	ENSG00000114354	TFG
ENST00000307388	951	-3.518575723	0.02058263	NA	Down	ENSG00000171944	OR52A5
ENST00000374278	3097	1.902449199	0.020616555	0.525826895	Ups	ENSG00000158014	SLC30A2
ENST00000400933	1670	2.603055445	0.020628669	0.525826895	Ups	ENSG00000215795	RP11-488L18.3
ENST00000361952	1657	2.255744071	0.020666257	0.525826895	Ups	ENSG00000198783	ZNF830
ENST00000333896	9075	-1.471646142	0.020689049	0.525826895	Down	ENSG00000115306	SPTBN1
ENST00000557555	817	3.16448613	0.020701258	0.525826895	Ups	ENSG00000135424	ITGA7
ENST00000335895	897	-1.796963768	0.02074962	0.526105679	Down	ENSG00000145741	BTF3
ENST00000429338	1596	-3.475015818	0.02076259	NA	Down	ENSG00000179846	NKPD1
ENST00000598038	2917	-3.508460913	0.020763136	NA	Down	ENSG00000130299	GTPBP3
ENST00000595510	1301	3.419312938	0.020784006	NA	Ups	ENSG00000090554	FLT3LG
ENST00000334654	1474	-3.511549946	0.020784531	NA	Down	ENSG00000100604	CHGA
ENST00000449115	551	1.482606215	0.020820047	0.526917004	Ups	ENSG00000181741	FDX1P1
ENST00000295033	1730	-3.252763745	0.020868332	0.526917004	Down	ENSG00000162931	TRIM17
ENST00000361927	5177	-3.467202825	0.02087445	NA	Down	ENSG00000198756	COLGALT2
ENST00000461131	530	2.224631722	0.020893952	0.526917004	Ups	ENSG00000205085	FAM71F2
ENST00000469610	795	-3.509838057	0.02093043	NA	Down	ENSG00000213726	RPS2P52
ENST00000285420	3298	-3.459196332	0.020944669	NA	Down	ENSG00000155100	OTUD6B
ENST00000446507	759	-3.459196332	0.020944669	NA	Down	ENSG00000171611	PTCRA
ENST00000507082	1578	3.465792507	0.020975604	NA	Ups	ENSG00000197530	MIB2
ENST00000433559	3950	-3.50864526	0.021003472	NA	Down	ENSG00000177303	CASKIN2
ENST00000338356	6636	1.55181052	0.021020747	0.52897477	Ups	ENSG00000135678	CPM
ENST00000495451	2701	3.079650374	0.021193602	0.52897477	Ups	ENSG00000049239	H6PD
ENST00000525711	572	-3.464312704	0.021330009	NA	Down	ENSG00000154127	UBASH3B
ENST00000446718	6372	-2.876258877	0.021345276	0.52897477	Down	ENSG00000105778	AVL9
ENST00000488803	879	2.219333636	0.021351669	0.52897477	Ups	ENSG00000240342	RPS2P5
ENST00000460758	1297	-3.206023707	0.021352355	0.52897477	Down	ENSG00000164054	SHISA5

ENST00000553894	1626	1.459068062	0.021353084	0.52897477	Ups	ENSG00000198208	RPS6KL1
ENST00000547115	749	3.437556449	0.021361299	NA	Ups	ENSG00000120832	MTERFD3
ENST00000567542	3542	-2.777883683	0.02141061	NA	Down	ENSG00000132600	PRMT7
ENST00000216968	1348	2.241156221	0.021427635	0.52897477	Ups	ENSG00000101000	PROCR
ENST00000503674	2426	1.692813298	0.021455978	0.52897477	Ups	ENSG00000073578	SDHA
ENST00000400762	1736	2.14248604	0.021461554	0.52897477	Ups	ENSG00000159674	SPON2
ENST00000568983	726	-1.09269056	0.021480731	0.52897477	Down	ENSG00000187741	FANCA
ENST00000429888	7325	1.739819655	0.021498021	0.52897477	Ups	ENSG00000114857	NKTR
ENST00000343150	2177	-3.268395034	0.021501817	0.52897477	Down	ENSG00000135047	CTSL
ENST00000505346	707	3.452612243	0.021562843	NA	Ups	ENSG00000205571	SMN2
ENST00000568199	613	1.842998561	0.021628811	0.531056258	Ups	ENSG00000140939	NOL3
ENST00000508243	1070	-3.503907617	0.02168252	NA	Down	ENSG00000164124	TMEM144
ENST00000488329	538	1.522706035	0.021684068	0.531056258	Ups	ENSG00000133026	MYH10
ENST00000582782	562	-3.449949518	0.021709784	NA	Down	ENSG00000265681	RPL17
ENST00000522833	752	2.663264925	0.021715515	NA	Ups	ENSG00000197265	GTF2E2
ENST00000377673	1582	-3.450742583	0.021717544	NA	Down	ENSG00000204856	FAM216A
ENST00000586636	938	-3.450742583	0.021717544	NA	Down	ENSG00000099622	CIRBP
ENST00000409740	1418	1.195936796	0.021726212	0.531056258	Ups	ENSG00000138079	SLC3A1
ENST00000572192	856	-2.193896752	0.021779021	0.531056258	Down	ENSG00000108509	CAMTA2
ENST00000366277	1897	1.93535668	0.021817503	0.531056258	Ups	ENSG00000138111	TMEM180
ENST00000481718	3115	-1.623238507	0.021833946	0.531056258	Down	ENSG00000044446	PHKA2
ENST00000525966	950	1.399276743	0.021850595	0.531056258	Ups	ENSG00000255040	RP11-677N16.1
ENST00000416832	224	3.427631593	0.021881212	NA	Ups	ENSG00000238008	COX6CP10
ENST00000258774	2935	3.114580901	0.021922806	NA	Ups	ENSG00000136273	HUS1
ENST00000273261	5273	-3.4805491	0.021925284	NA	Down	ENSG00000144749	LRIG1
ENST00000270221	876	-3.444348618	0.021947878	NA	Down	ENSG00000142227	EMP3
ENST00000476149	5674	1.270322002	0.021960245	0.531746286	Ups	ENSG00000088205	DDX18
ENST00000512377	751	-1.798501312	0.021973228	0.531746286	Down	ENSG00000248375	RP11-177B4.1
ENST00000311412	4615	-2.276802109	0.022056542	0.531746286	Down	ENSG00000173083	HPSE
ENST00000554111	2225	1.615267243	0.022066479	0.531746286	Ups	ENSG00000066629	EML1
ENST00000556167	601	1.912337187	0.022067924	0.531746286	Ups	ENSG00000100902	PSMA6
ENST00000558253	879	3.431386328	0.022151995	NA	Ups	ENSG00000104133	SPG11
ENST00000532283	590	-1.411066738	0.022232092	0.533928595	Down	ENSG00000174483	BBS1
ENST00000263849	3352	-3.474788696	0.022234461	NA	Down	ENSG00000104427	ZC2HC1A
ENST00000525130	1538	-2.012033859	0.022238455	0.533928595	Down	ENSG00000010361	FUZ
ENST00000536439	1426	-1.704268753	0.02227232	0.533928595	Down	ENSG00000111325	OGFOD2
ENST00000371045	3979	-1.708291086	0.022348176	0.534598766	Down	ENSG00000184588	PDE4B

ENST00000463847	969	3.418106083	0.022360512	NA	Ups	ENSG00000156639	ZFAND3
ENST00000317078	963	3.395562794	0.022374027	NA	Ups	ENSG00000181001	OR52N1
ENST00000361134	2124	-1.325974523	0.022376256	0.534598766	Down	ENSG00000198569	SLC34A3
ENST00000334271	2201	1.708578337	0.022469432	0.535914987	Ups	ENSG00000152056	AP1S3
ENST00000261973	4426	3.465522244	0.022471024	NA	Ups	ENSG00000119707	RBM25
ENST00000360798	1963	3.463501345	0.022627244	NA	Ups	ENSG00000105699	LSR
ENST00000465458	799	1.916674217	0.022657384	0.539483427	Ups	ENSG00000133026	MYH10
ENST00000379056	1101	-3.464735044	0.022728974	NA	Down	ENSG00000133112	TPT1
ENST00000263636	6886	1.076100659	0.022753933	0.540867135	Ups	ENSG00000054219	LY75
ENST00000288548	3043	1.387210121	0.022808051	0.541239292	Ups	ENSG00000119771	KLHL29
ENST00000481719	754	3.418464718	0.022844477	NA	Ups	ENSG00000135945	REV1
ENST00000420432	3202	-2.038649965	0.022868934	0.54177043	Down		
ENST00000342531	2239	-3.458758896	0.022901421	NA	Down	ENSG00000188199	NUTM2B
ENST00000377477	3511	-3.460437375	0.022901856	NA	Down	ENSG00000196774	ANKRD20A1
ENST00000368465	3144	-3.466812144	0.022926327	NA	Down	ENSG00000163346	PBXIP1
ENST00000346144	1316	-3.430382959	0.022928132	NA	Down	ENSG00000099866	MADCAM1
ENST00000220751	2584	-3.420958786	0.022945404	NA	Down	ENSG00000104312	RIPK2
ENST00000371225	2068	-2.815181592	0.02294672	0.541789006	Down	ENSG00000184292	TACSTD2
ENST00000533522	1623	3.44623155	0.023058949	NA	Ups	ENSG00000148057	IDNK
ENST00000492671	664	2.963664715	0.023059513	NA	Ups	ENSG00000056972	TRAF3IP2
ENST00000353214	2498	-1.414214453	0.023129922	0.543379411	Down	ENSG00000136950	ARPC5L
ENST00000532860	968	1.695362397	0.023181906	0.543692959	Ups	ENSG00000172273	HINFP
ENST00000334478	586	3.404134477	0.023210003	NA	Ups	ENSG00000123349	PFDN5
ENST00000415001	2555	3.404134477	0.023210003	NA	Ups	ENSG00000241258	CRCP
ENST00000455687	5916	-2.473849929	0.023212536	NA	Down		
ENST00000494165	532	-3.416464907	0.023265683	NA	Down	ENSG00000168878	SFTPB
ENST00000484446	727	2.569707292	0.023265776	NA	Ups	ENSG00000243022	RP11-221E20.4
ENST00000481071	1905	-3.452165232	0.023273176	NA	Down	ENSG00000146205	ANO7
ENST00000351018	1319	3.441288126	0.023320515	NA	Ups	ENSG00000177105	RHOG
ENST00000295461	5298	1.720229176	0.023323387	0.546101007	Ups	ENSG00000163293	NIPAL1
ENST00000506706	2098	-3.412086716	0.023394943	NA	Down	ENSG00000063978	RNF4
ENST00000571813	10076	-3.418978847	0.023436523	NA	Down	ENSG00000171282	RP11-1055B8.7
ENST00000372165	2586	-3.245754748	0.023485675	0.547994647	Down	ENSG00000172426	RSPH9
ENST00000399702	2748	1.159154114	0.023506437	0.547994647	Ups	ENSG00000215154	AC141586.5
ENST00000436861	681	1.144175302	0.023552735	0.547994647	Ups	ENSG00000228663	PSMD10P1
ENST00000426053	2808	1.387363346	0.023560031	0.547994647	Ups	ENSG00000100342	APOL1
ENST00000317951	4687	-1.070827463	0.023633586	0.548068234	Down	ENSG00000179846	NKPD1

ENST00000525504	1232	1.107778894	0.023676633	0.548068234	Ups	ENSG00000137500	CCDC90B
ENST00000369763	3475	1.367936389	0.023722808	0.548068234	Ups	ENSG00000121931	LRIF1
ENST00000346213	1671	3.367232007	0.023742139	NA	Ups	ENSG00000108774	RAB5C
ENST00000565751	1671	3.367232007	0.023742139	NA	Ups		
ENST00000479327	332	1.63086104	0.023761753	0.548068234	Ups	ENSG00000240925	RPS20P31
ENST00000537369	414	1.183836354	0.02380281	0.548068234	Ups	ENSG00000256379	TRAV8-5
ENST00000350026	7971	-1.881614782	0.02381223	0.548068234	Down	ENSG00000049618	ARID1B
ENST00000295598	3654	-3.437712421	0.023849771	NA	Down	ENSG00000163399	ATP1A1
ENST00000378383	2037	3.356398816	0.023850286	NA	Ups	ENSG00000102531	FNDC3A
ENST00000398022	3978	3.38631841	0.023865732	NA	Ups	ENSG00000101337	TM9SF4
ENST00000542200	2569	-3.451095922	0.023879183	NA	Down	ENSG00000103723	AP3B2
ENST00000463490	1925	-3.445827769	0.023898136	NA	Down	ENSG00000101222	SPEF1
ENST00000509041	2056	2.579295804	0.023936519	NA	Ups	ENSG00000173542	MOB1B
ENST00000330539	2086	-2.8180119	0.023963343	0.548068234	Down	ENSG00000183154	RP11-863K10.7
ENST00000503050	6559	-1.007948288	0.024039127	0.548068234	Down	ENSG00000161021	MAML1
ENST00000338051	4270	1.551087502	0.024104312	0.548068234	Ups	ENSG00000181904	C5orf24
ENST00000513458	6876	2.275205454	0.024142253	0.548068234	Ups	ENSG00000153006	SREK1IP1
ENST00000376974	785	-1.352973836	0.024205367	0.548068234	Down	ENSG00000204659	CBY3
ENST00000348075	7233	3.422567193	0.024207105	NA	Ups	ENSG00000105426	PTPRS
ENST00000304521	5009	-2.945357138	0.024254145	NA	Down	ENSG00000170322	NFRKB
ENST00000267889	5031	-1.067171072	0.024273416	0.548068234	Down	ENSG00000140323	DISP2
ENST00000463448	1589	3.384431097	0.024274871	NA	Ups	ENSG00000137210	TMEM14B
ENST00000394848	1796	2.419641227	0.024285584	0.548068234	Ups	ENSG00000141298	SSH2
ENST00000530508	1419	-1.192801364	0.02428562	0.548068234	Down	ENSG00000186523	FAM86B1
ENST00000597447	681	-2.932791474	0.024286754	NA	Down	ENSG00000083844	ZNF264
ENST00000487311	563	-2.579497114	0.024397069	NA	Down	ENSG00000087087	SRRT
ENST00000395634	1468	-2.973557721	0.024398555	NA	Down	ENSG00000134986	NREP
ENST00000427635	6391	1.536388335	0.02440072	0.548068234	Ups	ENSG00000148634	HERC4
ENST00000432606	568	-2.220636737	0.024429929	0.548068234	Down	ENSG00000015568	RGPD5
ENST00000455695	568	-2.220636737	0.024429929	0.548068234	Down	ENSG00000183054	RGPD6
ENST00000462438	3251	2.864068553	0.024453461	0.548068234	Ups	ENSG00000101146	RAE1
ENST00000496200	2283	1.922589599	0.024464272	0.548068234	Ups	ENSG00000106348	IMPDH1
ENST00000513146	763	1.734150517	0.024476172	0.548068234	Ups	ENSG00000183718	TRIM52
ENST00000316517	867	-2.737823251	0.024538663	NA	Down	ENSG00000180913	OR56B3P
ENST00000464655	1109	2.182590842	0.024546051	0.548068234	Ups	ENSG00000226356	RPS6P20
ENST00000309863	7537	-1.93288869	0.024557084	0.548068234	Down	ENSG00000135968	GCC2
ENST00000604424	567	-1.677378463	0.024619672	0.548068234	Down	ENSG00000255730	CTC-435M10.3

ENST00000367419	2024	1.65457524	0.024646259	0.548068234	Ups	ENSG00000055211	GINM1
ENST00000592215	417	2.185116099	0.024667928	0.548068234	Ups	ENSG00000197256	KANK2
ENST00000493241	762	-2.801502737	0.024714461	NA	Down	ENSG00000214021	TTLL3
ENST00000424807	2822	3.416137101	0.024726562	NA	Ups	ENSG00000204577	LILRB3
ENST00000494921	2384	3.340820129	0.024746734	NA	Ups	ENSG00000125821	DTD1
ENST00000316436	1494	-1.30244524	0.024753788	0.548068234	Down	ENSG00000179564	LSMEM2
ENST00000575357	1494	-1.30244524	0.024753788	0.548068234	Down		
ENST00000482828	477	3.344839468	0.02482266	NA	Ups	ENSG00000130775	THEMIS2
ENST00000540098	4760	2.994361543	0.024838492	NA	Ups	ENSG00000150054	MPP7
ENST00000456004	607	-3.387921437	0.024862943	NA	Down	ENSG00000165752	STK32C
ENST00000488683	2723	-3.387921437	0.024862943	NA	Down	ENSG00000169504	CLIC4
ENST00000509557	999	3.371714958	0.024869043	NA	Ups	ENSG00000249188	ENPP7P1
ENST00000479068	466	2.693437632	0.024870595	NA	Ups	ENSG00000133114	GPALPP1
ENST00000346437	3067	-3.35877181	0.024891364	NA	Down	ENSG00000013725	CD6
ENST00000480419	2328	-3.35877181	0.024891364	NA	Down	ENSG00000163492	CCDC141
ENST00000479494	1332	-3.057860319	0.024904797	NA	Down		
ENST00000463519	2704	2.063831682	0.024907593	0.548068234	Ups	ENSG00000171163	ZNF692
ENST00000590308	3720	-1.936689254	0.024981506	0.548068234	Down	ENSG00000168675	LDLRAD4
ENST00000542987	951	-3.412355175	0.025017201	NA	Down	ENSG00000125812	GZF1
ENST00000532027	4881	1.413886887	0.025030197	0.548068234	Ups	ENSG00000179532	DNHD1
ENST00000569504	2009	-2.519757855	0.025033173	NA	Down	ENSG00000102904	TSNAXIP1
ENST00000590714	4686	-1.059836906	0.025034541	0.548068234	Down	ENSG00000179943	FIZ1
ENST00000412164	270	2.708666559	0.02503678	NA	Ups	ENSG00000232875	HMGN2P35
ENST00000518375	3350	2.828143214	0.02503727	0.548068234	Ups	ENSG00000147650	LRP12
ENST00000354685	3205	2.547322877	0.025068354	0.548068234	Ups	ENSG00000179532	DNHD1
ENST00000442185	627	-3.425956627	0.025108819	NA	Down	ENSG00000171984	C20orf196
ENST00000522255	931	3.186184854	0.025127395	0.548068234	Ups	ENSG00000197217	ENTPD4
ENST00000369466	9462	1.406162849	0.025148473	0.548068234	Ups	ENSG00000116830	TTF2
ENST00000494948	766	-3.353309131	0.025153771	NA	Down	ENSG00000142687	KIAA0319L
ENST00000403136	1778	2.649747691	0.025178078	0.548068234	Ups	ENSG00000079385	CEACAM1
ENST00000590603	388	-3.413529351	0.025197877	NA	Down	ENSG00000125753	VASP
ENST00000575079	583	1.868761457	0.02522494	0.548068234	Ups	ENSG00000142507	PSMB6
ENST00000442987	3812	1.199977653	0.025225466	0.548068234	Ups	ENSG00000233750	CICP27
ENST00000355069	1554	-3.377650471	0.025231176	NA	Down	ENSG00000197358	BNIP3P1
ENST00000525704	2474	2.460736295	0.025233397	0.548068234	Ups	ENSG00000149571	KIRREL3
ENST00000381846	1542	2.085019303	0.025246336	0.548068234	Ups	ENSG00000121897	LIAS
ENST00000504720	853	3.334431238	0.025280942	NA	Ups	ENSG00000113296	THBS4

ENST00000380265	3781	1.817091128	0.025295819	0.548068234	Ups	ENSG00000138642	HERC6
ENST00000589911	769	-1.608969085	0.025328042	0.548068234	Down	ENSG00000184922	FMNL1
ENST00000268695	2363	3.354713202	0.025334877	NA	Ups	ENSG00000141012	GALNS
ENST00000591545	5193	2.294768959	0.025353484	0.548068234	Ups	ENSG00000127616	SMARCA4
ENST00000331442	790	-2.246606044	0.025354777	0.548068234	Down	ENSG00000184357	HIST1H1B
ENST00000409321	735	-3.168716934	0.025383667	NA	Down	ENSG00000187514	PTMA
ENST00000510979	2952	2.136415528	0.02543827	0.548744938	Ups	ENSG00000183474	GTF2H2C
ENST00000299667	2799	-3.411975966	0.025445272	NA	Down	ENSG00000166526	ZNF3
ENST00000423119	10098	-3.401354185	0.025461829	NA	Down	ENSG00000150995	ITPR1
ENST00000523465	649	1.679383482	0.025464074	0.548744938	Ups	ENSG00000070501	POLB
ENST00000272348	625	3.35647104	0.025487641	NA	Ups	ENSG00000143977	SNRPG
ENST00000340625	2272	1.652530911	0.025513947	0.548813598	Ups	ENSG00000173681	CXorf23
ENST00000495194	2861	2.818881675	0.025587874	0.548813598	Ups		
ENST00000479046	711	-3.379931166	0.02565393	NA	Down	ENSG00000107614	TRDMT1
ENST00000331552	1293	-2.752904606	0.025663991	0.548813598	Down	ENSG00000184162	NR2C2AP
ENST00000522792	2044	1.725083966	0.025665878	0.548813598	Ups	ENSG00000044115	CTNNA1
ENST00000572382	989	-1.113445423	0.025670497	0.548813598	Down	ENSG00000108523	RNF167
ENST00000339307	1403	-2.91247292	0.025705327	NA	Down	ENSG00000115232	ITGA4
ENST00000584377	2891	2.286586268	0.025745496	0.548925171	Ups	ENSG00000141551	CSNK1D
ENST00000575298	665	3.352688066	0.025783652	NA	Ups	ENSG00000185722	ANKFY1
ENST00000340611	2915	-2.725296475	0.025793709	NA	Down	ENSG00000106009	BRAT1
ENST00000444242	1191	3.343613853	0.025818695	NA	Ups	ENSG00000227289	HSFY3P
ENST00000481915	4791	2.295085209	0.02592276	NA	Ups	ENSG00000144635	DYNC1LI1
ENST00000481392	564	2.35837424	0.025924878	0.550584688	Ups	ENSG00000165055	METTL2B
ENST00000236698	5951	2.040788575	0.025934873	0.550584688	Ups	ENSG00000118007	STAG1
ENST00000534816	655	-1.492887905	0.025940708	0.550584688	Down	ENSG00000254618	TMED10P1
ENST00000397745	2148	2.879529693	0.025954901	NA	Ups	ENSG00000197646	PDCD1LG2
ENST00000301067	19419	-1.731303365	0.025982257	0.550636025	Down	ENSG00000167548	KMT2D
ENST00000428311	4505	3.350637672	0.026016574	NA	Ups	ENSG00000196826	ZNF709
ENST00000544396	2822	3.357054265	0.026053943	NA	Ups	ENSG00000155324	GRAMD3
ENST00000469420	2213	-3.353918205	0.026061221	NA	Down	ENSG00000183741	CBX6
ENST00000372214	645	1.423456666	0.026076598	0.551421868	Ups	ENSG00000233668	RP11-571F15.3
ENST00000395703	718	3.323991603	0.026116119	NA	Ups	ENSG00000177427	MIEF2
ENST00000379757	2964	3.337047791	0.02614101	NA	Ups	ENSG00000239264	TXNDC5
ENST00000515303	1730	1.479828662	0.026142648	0.55154325	Ups	ENSG00000163322	FAM175A
ENST00000338883	4635	-3.396612868	0.026183583	NA	Down	ENSG00000180815	MAP3K15
ENST00000505164	1514	-3.396612868	0.026183583	NA	Down	ENSG00000128050	PAICS

ENST00000466422	718	-2.600948466	0.026203741	NA	Down	ENSG00000213920	MDP1
ENST00000252818	1870	-1.215634916	0.026256287	0.552631787	Down	ENSG00000130522	JUND
ENST00000458603	455	1.184521332	0.026272787	0.552631787	Ups	ENSG00000116044	NFE2L2
ENST00000552903	607	-3.357774291	0.026305329	NA	Down	ENSG00000065357	DGKA
ENST00000368073	4106	-3.387676683	0.026316964	NA	Down	ENSG00000132716	DCAF8
ENST00000420274	5638	-3.387676683	0.026316964	NA	Down	ENSG00000169499	PLEKHA2
ENST00000514951	1308	2.075411409	0.026327039	0.552938494	Ups	ENSG00000172062	SMN1
ENST00000344528	5044	-3.328284247	0.026375701	NA	Down	ENSG00000130147	SH3BP4
ENST00000460625	1937	1.159203286	0.026444222	0.552938494	Ups	ENSG00000114638	UPK1B
ENST00000542664	3564	-3.388535026	0.026464242	NA	Down	ENSG00000102468	HTR2A
ENST00000570182	3549	-3.388535026	0.026464242	NA	Down	ENSG00000140995	DEF8
ENST00000578826	962	1.594838614	0.026476553	0.552938494	Ups	ENSG00000188895	MSL1
ENST00000568812	878	-1.243796272	0.026487281	0.552938494	Down		
ENST00000469830	1691	3.377847619	0.026504143	NA	Ups	ENSG00000187260	WDR86
ENST00000356083	7790	3.315979537	0.026513316	NA	Ups	ENSG00000196739	COL27A1
ENST00000440471	797	2.264375824	0.02652313	0.552938494	Ups	ENSG00000225505	RP11-330C7.3
ENST00000379389	711	-2.887792861	0.026606129	NA	Down	ENSG00000187608	ISG15
ENST00000457064	535	-2.818208945	0.026637182	0.553570895	Down	ENSG00000114738	MAPKAPK3
ENST00000405954	3461	-1.028484224	0.02664276	0.553570895	Down	ENSG00000188542	DUSP28
ENST00000515398	584	-2.181757517	0.026675183	0.553570895	Down	ENSG00000109814	UGDH
ENST00000250489	1961	3.334513615	0.026676355	NA	Ups	ENSG00000165782	TMEM55B
ENST00000468552	5120	2.7990908	0.026711204	0.553570895	Ups	ENSG00000186976	EFCAB6
ENST00000479080	519	-3.349316866	0.026721083	NA	Down	ENSG00000170004	CHD3
ENST00000397981	3430	-3.375209563	0.026732826	NA	Down	ENSG00000076984	MAP2K7
ENST00000566501	3128	-3.375209563	0.026732826	NA	Down	ENSG00000184110	EIF3C
ENST00000452596	1313	-2.559246972	0.02679315	0.553570895	Down		
ENST00000397865	2127	2.848807813	0.026827383	0.553570895	Ups	ENSG00000197971	MBP
ENST00000450744	2515	2.894952778	0.026828834	0.553570895	Ups		
ENST00000303004	1837	-1.270968127	0.026897662	0.554008913	Down	ENSG00000172216	CEBPB
ENST00000398841	1588	-3.377793165	0.026913639	NA	Down	ENSG00000090238	YPEL3
ENST00000576704	2822	2.870682242	0.026928802	0.554008913	Ups		
ENST00000345063	5479	3.333389478	0.02693605	NA	Ups	ENSG00000188001	TPRG1
ENST00000585605	586	3.297934425	0.0269606	NA	Ups	ENSG00000174917	C19orf70
ENST00000309180	1786	-2.46626693	0.027014287	NA	Down	ENSG00000174946	GPR171
ENST00000348165	5229	-3.342642896	0.02706455	NA	Down	ENSG00000009335	UBE3C
ENST00000315183	8303	3.328657336	0.027076359	NA	Ups	ENSG00000090905	TNRC6A
ENST00000460168	2215	1.33923198	0.027094611	0.554516209	Ups	ENSG00000148634	HERC4

ENST00000572991	2197	1.682131233	0.027124443	0.554516209	Ups	ENSG00000228696	ARL17B
ENST00000418851	412	3.294649493	0.027127652	NA	Ups	ENSG00000235937	AC008280.1
ENST00000264258	1865	1.451136222	0.027136808	0.554516209	Ups	ENSG00000071082	RPL31
ENST00000537927	5719	2.813233665	0.027211252	NA	Ups	ENSG00000110841	PPFIBP1
ENST00000250111	3153	2.520527121	0.027226226	NA	Ups	ENSG00000129244	ATP1B2
ENST00000469660	636	3.362165068	0.027230372	NA	Ups	ENSG00000177989	ODF3B
ENST00000326397	2641	2.062340804	0.027255594	0.554516209	Ups	ENSG00000182552	RWDD4
ENST00000443790	730	-3.367029601	0.027269096	NA	Down	ENSG00000134109	EDEM1
ENST00000400776	2370	-2.629626531	0.027273406	0.554516209	Down		
ENST00000216373	5489	-2.539118799	0.027296439	0.554516209	Down	ENSG00000100485	SOS2
ENST00000524750	757	1.677547378	0.027306734	0.554516209	Ups	ENSG00000085117	CD82
ENST00000341105	3367	3.324585547	0.027307134	NA	Ups	ENSG00000179348	GATA2
ENST00000411406	450	2.330320952	0.0273275	0.554516209	Ups	ENSG00000180098	TRNAU1AP
ENST00000564844	3085	-3.367518886	0.027336709	NA	Down	ENSG00000140941	MAP1LC3B
ENST00000533948	981	-2.089383442	0.027343457	0.554516209	Down	ENSG00000179526	SHARPIN
ENST00000300850	6892	-1.194250824	0.027347516	0.554516209	Down	ENSG00000167395	ZNF646
ENST00000236051	1322	-3.332260131	0.027453097	NA	Down	ENSG00000117395	EBNA1BP2
ENST00000591728	532	-2.977192647	0.027483542	NA	Down	ENSG00000081923	ATP8B1
ENST00000459741	5826	-1.922244429	0.02751747	0.557159488	Down	ENSG00000160218	TRAPPC10
ENST00000578036	4318	-3.331149556	0.027521996	NA	Down	ENSG00000214176	PLEKHM1P
ENST00000561434	582	3.363685289	0.027558028	NA	Ups	ENSG00000177082	WDR73
ENST00000314088	1668	-2.188143704	0.027613465	0.558239243	Down	ENSG00000180573	HIST1H2AC
ENST00000503265	1501	2.9249762	0.027614367	NA	Ups	ENSG00000177084	POLE
ENST00000505015	2189	3.353401828	0.02764824	NA	Ups	ENSG00000138231	DBR1
ENST00000343407	5856	2.207910113	0.027650139	0.558239243	Ups	ENSG00000128536	CDHR3
ENST00000537089	1668	-3.326912479	0.027692717	NA	Down	ENSG00000165269	AQP7
ENST00000468323	894	-3.359296286	0.027705831	NA	Down	ENSG00000070087	PFN2
ENST00000515545	2635	3.355605011	0.027724277	NA	Ups	ENSG00000070476	ZXDC
ENST00000445197	310	2.199515999	0.027809606	0.558335953	Ups	ENSG00000229046	HMGN1P2
ENST00000597145	4257	3.353593922	0.027836819	NA	Ups	ENSG00000142002	DPP9
ENST00000358446	1754	1.109400743	0.027845729	0.558335953	Ups	ENSG00000196737	AC087392.1
ENST00000425046	1359	1.446729707	0.027865275	0.558335953	Ups	ENSG00000170364	SETMAR
ENST00000595101	1776	1.456319066	0.027867848	0.558335953	Ups	ENSG00000053501	USE1
ENST00000418596	9333	1.40618276	0.027906338	0.558335953	Ups	ENSG00000155744	FAM126B
ENST00000553521	2815	-1.17295932	0.027960687	0.558335953	Down	ENSG00000100650	SRSF5
ENST00000431118	2636	2.803359662	0.027963958	NA	Ups	ENSG00000119004	CYP20A1
ENST00000594962	4343	1.091238284	0.027977012	0.558335953	Ups	ENSG00000130304	SLC27A1

ENST00000308406	3149	3.330377402	0.027994855	NA	Ups	ENSG00000166484	MAPK7
ENST00000600213	1020	-1.119982146	0.028012023	0.558335953	Down	ENSG00000269028	MTRNR2L12
ENST00000573373	649	2.782375548	0.028017707	NA	Ups	ENSG00000170310	STX8
ENST00000367577	2340	-1.425065145	0.028061408	0.558529185	Down	ENSG00000162783	IER5
ENST00000392081	2062	-3.356923521	0.028108717	NA	Down	ENSG00000068903	SIRT2
ENST00000434377	5197	2.628457959	0.02813131	0.559129646	Ups	ENSG00000186017	ZNF566
ENST00000580295	594	2.236853952	0.028172547	NA	Ups	ENSG00000108592	FTSJ3
ENST00000462520	1011	-3.318005925	0.028184591	NA	Down	ENSG00000143458	GABPB2
ENST00000414473	2221	3.282392442	0.0282152	NA	Ups		
ENST00000340581	3594	3.342043795	0.0282243	NA	Ups	ENSG00000173698	GPR64
ENST00000581897	4125	3.342043795	0.0282243	NA	Ups		
ENST00000452476	1468	-2.809936848	0.028229685	NA	Down	ENSG00000145220	LYAR
ENST00000293677	3595	-2.436857071	0.028234645	0.560204882	Down	ENSG00000161847	RAVER1
ENST00000335251	13233	1.302399119	0.028332605	0.560204882	Ups	ENSG00000164066	INTU
ENST00000476049	2502	-1.384041193	0.028414986	0.560204882	Down	ENSG00000264058	KRT222
ENST00000281092	12119	1.624371193	0.028473502	0.560204882	Ups	ENSG00000151422	FER
ENST00000475317	696	-2.791844722	0.028474116	NA	Down	ENSG00000205664	RP11-706O15.1
ENST00000219542	915	-2.802299148	0.028511568	0.560204882	Down	ENSG00000103260	METRN
ENST00000568000	2528	-1.706717682	0.028575097	0.560204882	Down	ENSG00000174943	KCTD13
ENST00000244565	2986	-3.319793179	0.028585995	NA	Down	ENSG00000124602	UNC5CL
ENST00000468904	2249	-3.319793179	0.028585995	NA	Down	ENSG00000182489	XKRX
ENST00000513438	546	-3.319793179	0.028585995	NA	Down	ENSG00000168255	POLR2J3
ENST00000300107	4695	1.445194093	0.028591244	0.560204882	Ups	ENSG00000166855	CLPX
ENST00000540634	763	-3.349544033	0.028592302	NA	Down	ENSG00000105676	ARMC6
ENST00000393766	1789	2.094324419	0.028597431	0.560204882	Ups	ENSG00000052802	MSMO1
ENST00000552666	1316	1.308766405	0.028619678	0.560204882	Ups	ENSG00000257957	QRSL1P3
ENST00000391352	1554	-1.781795682	0.028623317	0.560204882	Down	ENSG00000212657	KRTAP16-1
ENST00000572734	1554	-1.781795682	0.028623317	0.560204882	Down		
ENST00000557140	1400	-3.346583804	0.028645068	NA	Down	ENSG00000100906	NFKBIA
ENST00000338825	1020	-2.418228661	0.028713367	NA	Down	ENSG00000187840	EIF4EBP1
ENST00000359596	15117	-3.341784282	0.028752032	NA	Down	ENSG00000196218	RYR1
ENST00000456757	596	2.390254922	0.028774201	0.560827789	Ups	ENSG00000111490	TBC1D30
ENST00000610032	5918	-2.976859327	0.028785093	NA	Down	ENSG00000239665	RP11-295P9.3
ENST00000519751	596	-2.784824808	0.028789917	NA	Down	ENSG00000235531	RP11-383H13.1
ENST00000601356	2238	-1.962172204	0.028875266	0.560827789	Down	ENSG00000196961	AP2A1
ENST00000479690	824	-3.314040056	0.02889153	NA	Down	ENSG00000157954	WIPI2
ENST00000426094	2737	-3.283928162	0.028950216	NA	Down	ENSG00000007392	LUC7L

ENST00000562808	1549	-3.283928162	0.028950216	NA	Down	ENSG00000102984	ZNF821
ENST00000530790	561	3.304577924	0.028966947	NA	Ups	ENSG00000160948	VPS28
ENST00000371558	1796	3.286874329	0.028990014	NA	Ups	ENSG00000077721	UBE2A
ENST00000471259	942	1.238057007	0.029014139	0.560827789	Ups	ENSG00000185608	MRPL40
ENST00000419125	637	2.618115747	0.029056293	NA	Ups	ENSG00000236264	RPL26P30
ENST00000552999	446	3.259833268	0.029092189	NA	Ups	ENSG00000257896	RP11-210N13.1
ENST00000490751	762	-2.349889869	0.029094733	NA	Down	ENSG00000198728	LDB1
ENST00000594854	893	1.951226646	0.029095044	0.560827789	Ups	ENSG00000161652	IZUMO2
ENST00000443808	5273	-1.412164755	0.029112254	0.560827789	Down	ENSG00000069020	MAST4
ENST00000473409	1550	-3.331982392	0.029130126	NA	Down	ENSG00000181652	ATG9B
ENST00000273968	1361	1.64743371	0.029139789	0.560827789	Ups	ENSG00000145337	PYURF
ENST00000338163	2792	3.331670858	0.029200146	NA	Ups	ENSG00000131051	RBM39
ENST00000600704	467	-1.830069483	0.029216102	0.560827789	Down	ENSG00000171606	ZNF274
ENST00000561251	1951	-1.058754854	0.029242237	0.560827789	Down	ENSG00000022976	ZNF839
ENST00000594696	1407	1.754275371	0.029254028	0.560827789	Ups	ENSG00000133246	PRAM1
ENST00000477367	502	-3.308968357	0.029268134	NA	Down	ENSG00000106526	ACTR3C
ENST00000300113	2382	1.083351043	0.029281696	0.560827789	Ups	ENSG00000166869	CHP2
ENST00000427880	1047	1.955159683	0.029281703	0.560827789	Ups	ENSG00000203814	HIST2H2BF
ENST00000584281	1047	1.955159683	0.029281703	0.560827789	Ups		
ENST00000393728	2243	1.271444008	0.02928892	0.560827789	Ups	ENSG00000122203	KIAA1191
ENST00000413698	1808	-2.09949466	0.029293609	0.560827789	Down	ENSG00000185811	IKZF1
ENST00000559228	9238	1.245017271	0.029323312	0.560827789	Ups	ENSG00000128923	FAM63B
ENST00000429644	3461	-1.600025858	0.029339422	0.560827789	Down	ENSG00000114480	GBE1
ENST00000520482	2883	2.770434092	0.029340054	NA	Ups	ENSG00000105339	DENND3
ENST00000459629	7937	1.874418866	0.029348888	0.560827789	Ups	ENSG00000105778	AVL9
ENST00000530096	1926	1.811819885	0.029386476	0.560827789	Ups	ENSG00000149557	FEZ1
ENST00000378251	2648	-1.754418849	0.029412373	0.560827789	Down	ENSG00000130764	LRRC47
ENST00000322329	1092	2.456420995	0.029436752	NA	Ups	ENSG00000179673	RPRML
ENST00000596710	2692	1.371871092	0.029561664	0.562334842	Ups	ENSG00000269845	RP11-420K14.6
ENST00000344099	2949	2.429658837	0.029615519	NA	Ups	ENSG00000105708	ZNF14
ENST00000551964	5444	-3.325495644	0.02961889	NA	Down	ENSG00000120868	APAF1
ENST00000562641	1941	-3.325495644	0.02961889	NA	Down	ENSG00000090238	YPEL3
ENST00000221930	2769	-1.121557262	0.029621527	0.562334842	Down	ENSG00000105329	TGFB1
ENST00000417788	4213	2.049245233	0.029649868	0.562334842	Ups	ENSG00000100023	PPIL2
ENST00000302787	5462	2.058220222	0.029651254	0.562334842	Ups	ENSG00000168924	LETM1
ENST00000274242	7686	1.299957945	0.029722393	0.562567351	Ups	ENSG00000145592	RPL37
ENST00000415525	1735	3.318242897	0.029726188	NA	Ups		

ENST00000427477	1735	3.318242897	0.029726188	NA	Ups		
ENST00000426013	1880	1.633099338	0.029743651	0.562567351	Ups	ENSG00000185787	MORF4L1
ENST00000472653	912	3.285131948	0.029768206	NA	Ups	ENSG00000163959	SLC51A
ENST00000377996	2473	-1.670822872	0.029797382	0.562567351	Down	ENSG00000137103	TMEM8B
ENST00000443208	943	3.269044342	0.029853563	NA	Ups	ENSG00000234102	KRT19P4
ENST00000539449	2266	1.607962108	0.029886444	0.562567351	Ups	ENSG00000130347	RTN4IP1
ENST00000244360	2206	-1.832526279	0.029903381	0.562567351	Down	ENSG00000204618	RNF39
ENST00000432647	2206	-1.832526279	0.029903381	0.562567351	Down		
ENST00000510759	359	2.770346858	0.029941758	NA	Ups	ENSG00000250330	CTC-422A18.2
ENST00000295925	1118	-1.179232638	0.029996199	0.563060198	Down	ENSG00000163660	CCNL1
ENST00000408926	3174	1.260591166	0.030009604	0.563060198	Ups	ENSG00000221947	XKR9
ENST00000446874	3193	2.408469162	0.030069029	NA	Ups	ENSG00000228903	RASA4CP
ENST00000497545	4375	-1.772852157	0.030175202	0.563241416	Down	ENSG00000162650	ATXN7L2
ENST00000219919	3034	3.276979132	0.030194076	NA	Ups	ENSG00000103569	AQP9
ENST00000491593	704	3.276979132	0.030194076	NA	Ups	ENSG00000232361	RP11-632K21.3
ENST00000402883	4091	-3.322805897	0.030252138	NA	Down	ENSG00000171877	FRMD5
ENST00000292254	2163	2.756194664	0.030253098	0.563241416	Ups	ENSG00000160753	RUSC1
ENST00000431534	1464	3.264114178	0.030260101	NA	Ups	ENSG00000100413	POLR3H
ENST00000587342	802	-2.845762386	0.030265484	NA	Down	ENSG00000154832	CXXC1
ENST00000487943	3893	2.109946546	0.030286724	0.563241416	Ups	ENSG00000068885	IFT80
ENST00000290524	3576	-3.280878998	0.030306521	NA	Down	ENSG00000143390	RFX5
ENST00000469172	3356	-1.205939648	0.030319444	0.563241416	Down	ENSG00000096746	HNRNPH3
ENST00000216181	7501	-1.211512242	0.03032094	0.563241416	Down	ENSG00000100345	MYH9
ENST00000462592	3554	1.319305877	0.030326223	0.563241416	Ups	ENSG00000099219	ERMP1
ENST00000475407	4107	-1.493295851	0.0303317	0.563241416	Down	ENSG00000136935	GOLGA1
ENST00000528056	7218	3.309826819	0.030332064	NA	Ups	ENSG00000099725	PRKY
ENST00000453556	656	1.592121636	0.030364231	0.563241416	Ups	ENSG00000237719	RP1-179N16.3
ENST00000460900	1443	-2.314287696	0.030419519	0.563241416	Down	ENSG00000213145	CRIP1
ENST00000605557	1443	-2.314287696	0.030419519	0.563241416	Down		
ENST00000467157	2232	2.519390847	0.030423112	NA	Ups	ENSG00000065485	PDIA5
ENST00000490528	1405	3.300414182	0.030460511	NA	Ups	ENSG00000160993	ALKBH4
ENST00000395472	6779	-3.317806216	0.030525007	NA	Down	ENSG00000180008	SOCS4
ENST00000396945	8006	-3.275730076	0.030536755	NA	Down	ENSG00000029534	ANK1
ENST00000428824	1078	1.626066984	0.030603968	0.56399307	Ups	ENSG00000235602	POU5F1P3
ENST00000446339	566	-2.11082568	0.030719265	0.56399307	Down	ENSG00000143393	PI4KB
ENST00000349655	1120	-3.306075025	0.030748049	NA	Down	ENSG00000152795	HNRNPDL
ENST00000291576	3297	-1.824103758	0.030748821	0.56399307	Down	ENSG00000241945	PWP2

ENST00000560821	1024	3.258958664	0.030755409	NA		Ups	ENSG00000197299	BLM
ENST00000597790	718	-2.270394164	0.030776589		0.56399307	Down	ENSG00000131408	NR1H2
ENST00000564373	637	-3.303066959	0.030780082	NA		Down	ENSG00000186118	TEX38
ENST00000377974	4536	-2.529767592	0.030784539		0.56399307	Down	ENSG00000181652	ATG9B
ENST00000585170	3544	-2.389906763	0.030798927	NA		Down	ENSG00000108387	43712
ENST00000452210	237	3.299708057	0.030818792	NA		Ups	ENSG00000227239	AL121985.1
ENST00000487111	469	3.267865148	0.030834951	NA		Ups	ENSG00000241170	RP11-147I3.1
ENST00000581241	6094	1.711812151	0.030873493		0.56399307	Ups	ENSG00000141551	CSNK1D
ENST00000489906	694	2.563854139	0.030876343	NA		Ups	ENSG00000213585	VDAC1
ENST00000361723	3592	3.256642894	0.030883662	NA		Ups	ENSG00000166801	FAM111A
ENST00000409817	1895	-2.198178629	0.030902812		0.56399307	Down	ENSG00000121966	CXCR4
ENST00000528157	1153	-3.274230143	0.030927597	NA		Down	ENSG00000145050	MANF
ENST00000566546	1153	-3.274230143	0.030927597	NA		Down		
ENST00000358227	2755	-2.103545693	0.030938312		0.56399307	Down	ENSG00000137038	TMEM261
ENST00000592994	2770	2.057600912	0.030945581		0.56399307	Ups	ENSG00000267639	CICP19
ENST00000431592	555	2.792249259	0.031039795	NA		Ups	ENSG00000223700	AC104395.1
ENST00000532896	1676	3.253488257	0.031060326	NA		Ups	ENSG00000254858	MPV17L2
ENST00000565912	5796	1.67678807	0.031067834		0.56399307	Ups	ENSG00000006210	CX3CL1
ENST00000591765	1942	-2.165616608	0.031088237		0.56399307	Down	ENSG00000184451	CCR10
ENST00000548181	2440	1.03805839	0.031149015		0.56399307	Ups	ENSG00000111300	NAA25
ENST00000422370	5943	3.251681974	0.031153749	NA		Ups	ENSG00000104866	PPP1R37
ENST00000373416	2613	-3.29717403	0.031166074	NA		Down	ENSG00000134686	PHC2
ENST00000488418	750	-3.29717403	0.031166074	NA		Down	ENSG00000160087	UBE2J2
ENST00000491614	3433	1.314658941	0.031203798		0.56399307	Ups	ENSG00000165609	NUDT5
ENST00000444397	2002	1.660852548	0.031213242		0.56399307	Ups	ENSG00000112033	PPARD
ENST00000471152	471	-2.346619035	0.031234309	NA		Down	ENSG00000234851	RP11-3P17.3
ENST00000598671	1056	2.246676761	0.03123738		0.56399307	Ups	ENSG00000268442	HAVCR1P1
ENST00000376991	819	-3.295754753	0.031241588	NA		Down	ENSG00000175550	DRAP1
ENST00000400928	2503	-3.269959253	0.031291793	NA		Down	ENSG00000162572	SCNN1D
ENST00000439903	710	2.12179044	0.031330426		0.56399307	Ups	ENSG00000197446	CYP2F1
ENST00000480768	1278	2.816716141	0.031336524	NA		Ups	ENSG00000140307	GTF2A2
ENST00000479747	732	-1.800861944	0.031345622		0.56399307	Down	ENSG00000136840	ST6GALNAC4
ENST00000427641	1946	-3.304602493	0.031354679	NA		Down	ENSG00000114503	NCBP2
ENST00000491604	3106	-2.898269015	0.031414245		0.56399307	Down	ENSG00000100033	PRODH
ENST00000394455	4134	3.251210524	0.031426991	NA		Ups	ENSG00000072121	ZFYVE26
ENST00000588188	1209	3.251210524	0.031426991	NA		Ups	ENSG00000108946	PRKAR1A
ENST00000581236	408	3.250572467	0.031457466	NA		Ups	ENSG00000160606	TLCD1

ENST00000300469	1464	3.259715965	0.031536819	NA	Ups	ENSG00000125971	DYNLRB1
ENST00000351578	4750	3.259715965	0.031536819	NA	Ups	ENSG00000082074	FYB
ENST00000394081	2285	-3.294788776	0.031551701	NA	Down	ENSG00000131759	RARA
ENST00000443945	2070	-3.052078441	0.031552657	NA	Down	ENSG00000136141	LRCH1
ENST00000601613	2690	1.26470944	0.031595293	0.56399307	Ups	ENSG00000179134	SAMD4B
ENST00000415804	584	1.855285023	0.031605442	0.56399307	Ups	ENSG00000237170	RPS7P15
ENST00000449243	255	-2.567599678	0.031633287	0.56399307	Down	ENSG00000229436	AC073850.6
ENST00000568372	3388	1.795237775	0.031635152	0.56399307	Ups	ENSG00000260916	CCPG1
ENST00000395751	4653	1.375791624	0.031636613	0.56399307	Ups	ENSG00000072310	SREBF1
ENST00000469023	3916	2.334099651	0.031681579	NA	Ups	ENSG00000132681	ATP1A4
ENST00000507535	2775	1.299635213	0.031686521	0.56399307	Ups	ENSG00000250733	C8orf17
ENST00000368449	2879	3.282509083	0.031692432	NA	Ups	ENSG00000160691	SHC1
ENST00000370793	4502	-2.157007172	0.031704676	0.56399307	Down	ENSG00000077254	USP33
ENST00000599305	588	-2.81161472	0.031720239	0.56399307	Down	ENSG00000170956	CEACAM3
ENST00000608989	588	-2.81161472	0.031720239	0.56399307	Down		
ENST00000447404	6290	1.466144864	0.031772664	0.56399307	Ups	ENSG00000165209	STRBP
ENST00000572357	437	3.240871532	0.031781922	NA	Ups	ENSG00000262943	ALOX12P2
ENST00000571365	535	3.236640169	0.03181402	NA	Ups	ENSG00000108523	RNF167
ENST00000378799	2654	-3.258063995	0.031838101	NA	Down	ENSG00000182149	IST1
ENST00000392933	610	-3.258063995	0.031838101	NA	Down	ENSG00000182217	HIST2H4B
ENST00000392938	610	-3.258063995	0.031838101	NA	Down	ENSG00000183941	HIST2H4A
ENST00000566757	1844	3.241683719	0.031845361	NA	Ups	ENSG00000159461	AMFR
ENST00000370010	4644	-2.376193231	0.031869866	0.56399307	Down	ENSG00000112701	SENP6
ENST00000444743	746	-3.287498158	0.031874086	NA	Down	ENSG00000138297	TIMM23
ENST00000536150	1956	-3.287498158	0.031874086	NA	Down	ENSG00000111325	OGFOD2
ENST00000560773	468	2.869480846	0.03188309	NA	Ups	ENSG00000140319	SRP14
ENST00000583444	2882	-1.63765043	0.031909795	0.56399307	Down	ENSG00000141526	SLC16A3
ENST00000530091	307	3.252902361	0.031921095	NA	Ups	ENSG00000254915	RP11-263C24.3
ENST00000532145	2790	2.876643879	0.03192658	NA	Ups	ENSG00000154237	LRRK1
ENST00000561504	700	-1.335793949	0.031944247	0.56399307	Down	ENSG00000148671	ADIRF
ENST00000334571	2118	-1.50079956	0.032005566	0.56399307	Down	ENSG00000119723	COQ6
ENST00000510199	1100	1.204838402	0.032033955	0.56399307	Ups	ENSG00000204628	GNB2L1
ENST00000472487	7785	-2.951707358	0.032045565	NA	Down	ENSG00000162601	MYSM1
ENST00000556319	665	-1.087818653	0.032046047	0.56399307	Down	ENSG00000140553	UNC45A
ENST00000537896	1528	-2.183727625	0.032052684	NA	Down	ENSG00000235531	RP11-383H13.1
ENST00000496645	3563	-1.734193692	0.032130088	0.56399307	Down	ENSG00000085552	IGSF9
ENST00000356455	850	3.232730648	0.032143124	NA	Ups	ENSG00000175063	UBE2C

ENST00000429434	1945	3.232730648	0.032143124	NA	Ups	ENSG00000196387	ZNF140
ENST00000505354	1662	1.733723821	0.032145142	0.56399307	Ups	ENSG00000229894	GK3P
ENST00000582461	3641	3.276979406	0.032152515	NA	Ups	ENSG00000150656	CNDP1
ENST00000380590	1538	-1.293576968	0.032160573	0.56399307	Down	ENSG00000122696	SLC25A51
ENST00000404767	6959	-3.251914106	0.032193733	NA	Down	ENSG00000164880	INTS1
ENST00000295190	4390	2.398055898	0.03223611	0.56399307	Ups	ENSG00000163053	SLC16A14
ENST00000471389	11766	3.227430322	0.032285113	NA	Ups	ENSG00000140718	FTO
ENST00000326831	3192	1.681159544	0.032297324	0.56399307	Ups	ENSG00000118322	ATP10B
ENST00000450185	2975	1.961966888	0.032307501	0.56399307	Ups	ENSG00000144040	SFXN5
ENST00000533705	6055	2.861895929	0.032308522	NA	Ups	ENSG00000135018	UBQLN1
ENST00000341657	7418	1.99850511	0.032320867	0.56399307	Ups	ENSG00000185722	ANKFY1
ENST00000369219	3671	2.821658091	0.032356431	0.56399307	Ups	ENSG00000122497	NBPF14
ENST00000609116	12014	2.046761276	0.032359145	0.56399307	Ups	ENSG00000117569	PTBP2
ENST00000344649	3552	2.080612149	0.032360773	0.56399307	Ups	ENSG00000188897	CTD-3088G3.8
ENST00000378581	920	3.272268706	0.032368186	NA	Ups	ENSG00000165169	DYNLT3
ENST00000536169	3863	-2.641496505	0.032389214	0.56399307	Down	ENSG00000196338	NLGN3
ENST00000594570	552	-1.414508693	0.03239135	0.56399307	Down	ENSG00000269093	AC007773.3
ENST00000495308	808	1.957743341	0.03242399	0.56399307	Ups	ENSG00000160752	FDPS
ENST00000575282	5562	2.21902937	0.032490866	0.564458603	Ups	ENSG00000181523	SGSH
ENST00000596502	594	-3.276416856	0.032509776	NA	Down	ENSG00000105671	DDX49
ENST00000508652	1195	2.343742238	0.032540064	NA	Ups	ENSG00000198498	TMA16
ENST00000529896	494	-3.272966585	0.032545055	NA	Down	ENSG00000134802	SLC43A3
ENST00000574575	821	-3.272966585	0.032545055	NA	Down	ENSG00000085644	ZNF213
ENST00000575811	3519	-2.776606303	0.032553401	NA	Down		
ENST00000425096	629	-2.602524058	0.032587642	NA	Down	ENSG00000126001	CEP250
ENST00000288344	1303	-3.274863753	0.032590554	NA	Down	ENSG00000205581	HMGN1
ENST00000585933	3845	3.270298453	0.032618564	NA	Ups	ENSG00000153879	CEBPG
ENST00000597012	1073	-3.274714614	0.032644609	NA	Down	ENSG00000269845	RP11-420K14.6
ENST00000265140	5905	2.38253428	0.032664268	0.5654796	Ups	ENSG00000133302	ANKRD32
ENST00000557296	470	-3.244906739	0.032731442	NA	Down	ENSG00000258869	RP11-204N11.1
ENST00000382743	2543	-1.298546778	0.032793061	0.5654796	Down	ENSG00000142082	SIRT3
ENST00000473672	817	1.706010794	0.032798679	0.5654796	Ups	ENSG00000206199	ANKUB1
ENST00000534093	862	3.258650339	0.032889812	NA	Ups	ENSG00000149489	ROM1
ENST00000391666	2663	2.646760501	0.032890731	NA	Ups	ENSG00000272268	DKFZP761K2322
ENST00000463753	2964	1.087180235	0.032902803	0.5654796	Ups	ENSG00000144810	COL8A1
ENST00000517889	1381	-2.490043385	0.032957868	0.5654796	Down	ENSG00000222019	URAHP
ENST00000600634	461	3.224435671	0.032992715	NA	Ups	ENSG00000204514	ZNF814

ENST00000376854	2327	-1.799753045	0.033004017	0.5654796	Down	ENSG00000135045	C9orf40
ENST00000477110	5802	2.083831971	0.0330072	0.5654796	Ups	ENSG00000167393	PPP2R3B
ENST00000555746	558	-3.265823978	0.033032174	NA	Down	ENSG00000100883	SRP54
ENST00000557901	407	-3.268696513	0.033044493	NA	Down	ENSG00000166710	B2M
ENST00000256062	8805	2.248320609	0.033075158	0.5654796	Ups	ENSG00000133687	TMTC1
ENST00000368209	2171	3.259479271	0.033077412	NA	Ups	ENSG00000143321	HDGF
ENST00000296417	979	1.155844284	0.033091192	0.5654796	Ups	ENSG00000164032	H2AFZ
ENST00000445125	599	-1.600555562	0.033157186	0.5654796	Down	ENSG00000225840	AC010970.2
ENST00000456057	3947	-1.258986972	0.033172181	0.5654796	Down	ENSG00000124593	PRICKLE4
ENST00000435296	785	1.741750921	0.033177342	0.5654796	Ups	ENSG00000135245	HILPDA
ENST00000463499	571	1.384017643	0.033189133	0.5654796	Ups	ENSG00000240167	RPS7P7
ENST00000300413	1841	2.520750871	0.033192592	0.5654796	Ups	ENSG00000167088	SNRPD1
ENST00000571880	1020	3.259668526	0.033206937	NA	Ups	ENSG00000141542	RAB40B
ENST00000360215	2147	3.223525657	0.033247121	NA	Ups	ENSG00000197753	LHFPL5
ENST00000270625	614	3.219936265	0.033257539	NA	Ups	ENSG00000142534	RPS11
ENST00000319004	3748	-3.269448491	0.033364318	NA	Down	ENSG00000179409	GEMIN4
ENST00000575369	2830	-2.607414245	0.03336626	0.567176117	Down	ENSG00000132530	XAF1
ENST00000429705	5128	1.899817823	0.033372785	0.567176117	Ups	ENSG00000163788	SNRK
ENST00000594212	1132	3.217886744	0.033463512	NA	Ups	ENSG00000130479	MAP1S
ENST00000372405	1962	1.4074704	0.033499309	0.567603318	Ups	ENSG00000142949	PTPRF
ENST00000543430	1436	1.73285494	0.033516054	0.567603318	Ups	ENSG00000135124	P2RX4
ENST00000449200	742	1.373828136	0.033518928	0.567603318	Ups	ENSG00000228599	RPL7P52
ENST00000334256	8981	1.100685443	0.033612196	0.568057488	Ups	ENSG00000186432	KPNA4
ENST00000541774	4341	-1.095960773	0.033626484	0.568057488	Down	ENSG00000141736	ERBB2
ENST00000469063	510	2.722917868	0.033657031	NA	Ups	ENSG00000093072	CECR1
ENST00000367454	1815	-2.048118865	0.033712389	0.568477962	Down	ENSG00000116750	UCHL5
ENST00000476033	945	-3.23276046	0.033732875	NA	Down	ENSG00000132716	DCAF8
ENST00000548462	3032	2.162919618	0.033811181	0.568477962	Ups	ENSG00000166598	HSP90B1
ENST00000273963	5621	2.793431341	0.03386645	0.568477962	Ups	ENSG00000145332	KLHL8
ENST00000433140	1431	-2.046860244	0.033870898	0.568477962	Down	ENSG00000138080	EMILIN1
ENST00000340450	2592	3.243176543	0.033905571	NA	Ups	ENSG00000131236	CAP1
ENST00000586534	1746	-3.250777312	0.033929075	NA	Down	ENSG00000179262	RAD23A
ENST00000609686	27217	-2.66282312	0.033946961	NA	Down	ENSG00000273079	GRIN2B
ENST00000507479	649	-1.382095201	0.033955729	0.568477962	Down	ENSG00000186352	ANKRD37
ENST00000469150	3389	2.454647141	0.033994491	0.568477962	Ups	ENSG00000132128	LRRC41
ENST00000395227	1962	-2.151759308	0.034003997	0.568477962	Down	ENSG00000162594	IL23R
ENST00000483559	2392	-1.950636216	0.034014955	0.568477962	Down	ENSG00000159069	FBXW5

ENST00000409600	3383	3.245674632	0.0340341	NA	Ups	ENSG00000082153	BZW1
ENST00000572515	1929	1.159828221	0.034104175	0.56929294	Ups	ENSG00000104825	NFKBIB
ENST00000554584	20508	-3.015049161	0.034105807	NA	Down	ENSG00000054654	SYNE2
ENST00000503213	648	3.211460335	0.03410792	NA	Ups	ENSG00000109586	GALNT7
ENST00000507160	549	3.211460335	0.03410792	NA	Ups	ENSG00000164265	SCGB3A2
ENST00000444696	583	3.186530702	0.034135216	NA	Ups	ENSG00000138078	PREPL
ENST00000381873	4995	2.874227014	0.034293506	NA	Ups	ENSG00000101298	SNPH
ENST00000565928	3884	3.211955573	0.034354927	NA	Ups	ENSG00000235217	TSPY26P
ENST00000566592	2541	-3.241993423	0.034370225	NA	Down		
ENST00000342580	8155	3.238922034	0.034382838	NA	Ups	ENSG00000126001	CEP250
ENST00000438779	1579	-2.744072007	0.034403546	0.572920014	Down	ENSG00000219294	PIP5K1P1
ENST00000583369	841	-3.214250189	0.034423362	NA	Down	ENSG00000109062	SLC9A3R1
ENST00000560810	2944	1.415810064	0.034453129	0.572920014	Ups	ENSG00000187667	WHAMMP3
ENST00000455459	526	1.362367891	0.034456262	0.572920014	Ups	ENSG00000224448	GS1-259H13.7
ENST00000299767	2858	-3.21369778	0.034467795	NA	Down	ENSG00000166598	HSP90B1
ENST00000453002	341	1.725818093	0.034488271	0.572920014	Ups	ENSG00000243686	RPLP1P11
ENST00000436083	2639	1.990312816	0.034525026	0.572920014	Ups	ENSG00000196917	HCAR1
ENST00000308677	2677	3.207861746	0.034610523	NA	Ups	ENSG00000072062	PRKACA
ENST00000339852	1978	3.203208579	0.03461267	NA	Ups	ENSG00000188505	NCCRP1
ENST00000267918	1084	-1.631519163	0.034630032	0.573622573	Down	ENSG00000140350	ANP32A
ENST00000527614	5587	1.928699245	0.034689654	0.573622573	Ups	ENSG00000137713	PPP2R1B
ENST00000576276	5587	1.928699245	0.034689654	0.573622573	Ups		
ENST00000465584	3839	-3.244500537	0.034713969	NA	Down	ENSG00000114857	NKTR
ENST00000471355	602	-3.216200766	0.034734889	NA	Down	ENSG00000163466	ARPC2
ENST00000596251	1183	-2.807484007	0.034794863	NA	Down	ENSG00000160570	DEDD2
ENST00000493238	815	3.226815455	0.034820633	NA	Ups	ENSG00000082996	RNF13
ENST00000380516	4147	-3.207680269	0.034837624	NA	Down	ENSG00000137776	SLTM
ENST00000524409	492	-3.207680269	0.034837624	NA	Down	ENSG00000147535	PPAPDC1B
ENST00000440804	1960	2.584414209	0.034843964	0.575497963	Ups	ENSG00000129450	SIGLEC9
ENST00000540419	3468	-2.713500447	0.034893788	NA	Down	ENSG00000063015	SEZ6
ENST00000486499	5489	-1.0054523	0.034958321	0.575972563	Down	ENSG00000151006	PRSS53
ENST00000491463	567	1.774078755	0.034961552	0.575972563	Ups	ENSG00000164713	BRI3
ENST00000215812	2086	-3.242157937	0.034994206	NA	Down	ENSG00000100012	SEC14L3
ENST00000448451	2102	-3.242157937	0.034994206	NA	Down	ENSG00000131504	DIAPH1
ENST00000410097	1370	-1.28635696	0.03499549	0.575972563	Down	ENSG00000138382	METTL5
ENST00000401642	3176	-3.233141675	0.035003058	NA	Down	ENSG00000184178	SCFD2
ENST00000523812	2333	-3.233141675	0.035003058	NA	Down	ENSG00000250571	GLI4

ENST00000531121	672	-3.233141675	0.035003058	NA	Down	ENSG00000175550	DRAP1
ENST00000528573	585	-3.209364735	0.035008247	NA	Down	ENSG00000175467	SART1
ENST00000399568	1005	3.17196478	0.035027906	NA	Ups	ENSG00000242259	C22orf39
ENST00000599037	1005	3.17196478	0.035027906	NA	Ups		
ENST00000344419	8742	3.224794517	0.035034165	NA	Ups	ENSG00000009765	IYD
ENST00000488606	2127	3.224794517	0.035034165	NA	Ups	ENSG00000116898	MRPS15
ENST00000570439	4898	-1.746179925	0.035055857	0.576104843	Down	ENSG00000072818	ACAP1
ENST00000514871	2566	1.797684027	0.03509905	0.576104843	Ups	ENSG00000248540	RP11-247C2.2
ENST00000200652	2201	-1.782150904	0.035160463	0.576104843	Down	ENSG00000197208	SLC22A4
ENST00000585905	2176	2.271017716	0.0351965	0.576104843	Ups	ENSG00000104881	PPP1R13L
ENST00000434977	760	2.971018592	0.035253284	0.576104843	Ups	ENSG00000112137	PHACTR1
ENST00000435847	5457	2.559195845	0.035279909	NA	Ups	ENSG00000055163	CYFIP2
ENST00000432607	1285	1.749875223	0.035344237	0.576104843	Ups	ENSG00000233622	CYP2T2P
ENST00000409508	14167	-1.367082375	0.035347161	0.576104843	Down	ENSG00000105877	DNAH11
ENST00000321297	2243	-3.203537736	0.035370425	NA	Down	ENSG00000168010	ATG16L2
ENST00000392908	2242	-3.203537736	0.035370425	NA	Down	ENSG00000197119	SLC25A29
ENST00000506830	1978	-3.203537736	0.035370425	NA	Down	ENSG00000112659	CUL9
ENST00000539621	2242	-3.203537736	0.035370425	NA	Down	ENSG00000197119	SLC25A29
ENST00000562438	9789	1.939439313	0.035372677	0.576104843	Ups		
ENST00000329006	1930	-1.069415874	0.035477384	0.576104843	Down	ENSG00000185090	MANEAL
ENST00000311916	3131	-3.191349154	0.035479287	NA	Down	ENSG00000107815	C10orf2
ENST00000579211	411	-3.191349154	0.035479287	NA	Down	ENSG00000132470	ITGB4
ENST00000545187	2039	-1.630287789	0.035494805	0.576104843	Down	ENSG00000105655	ISYNA1
ENST00000582811	2039	-1.630287789	0.035494805	0.576104843	Down	ENSG00000105655	ISYNA1
ENST00000506882	3436	3.177945264	0.035532213	NA	Ups	ENSG00000145241	CENPC
ENST00000519164	463	1.478948948	0.035607745	0.576147864	Ups	ENSG00000254388	DUTP2
ENST00000445363	1205	1.033909287	0.035659274	0.576147864	Ups	ENSG00000235253	AC010240.2
ENST00000515125	1125	2.16869437	0.03566789	0.576147864	Ups	ENSG00000164347	GFM2
ENST00000324096	3419	3.212219322	0.035671519	NA	Ups	ENSG00000130479	MAP1S
ENST00000370205	9367	1.209623072	0.035693951	0.576147864	Ups	ENSG00000172339	ALG14
ENST00000497403	5400	1.350094912	0.035714384	0.576147864	Ups	ENSG00000134317	GRHL1
ENST00000592358	626	-3.229414107	0.035776022	NA	Down	ENSG00000167671	UBXN6
ENST00000359999	3659	2.669226707	0.035818373	NA	Ups	ENSG00000148204	CRB2
ENST00000299320	2722	-3.215600462	0.035911686	NA	Down	ENSG00000166246	C16orf71
ENST00000392029	1075	-3.215600462	0.035911686	NA	Down	ENSG00000116260	QSOX1
ENST00000563792	493	-3.215600462	0.035911686	NA	Down	ENSG00000103253	HAGHL
ENST00000604991	943	-3.215600462	0.035911686	NA	Down	ENSG00000271696	RP11-548K12.13

ENST00000538714	6487	1.379101618	0.035913823	0.576147864	Ups	ENSG00000052126	PLEKHA5
ENST00000303498	2075	3.212749014	0.035934117	NA	Ups	ENSG00000169814	BTD
ENST00000258080	2367	-1.428791658	0.035939981	0.576147864	Down	ENSG00000115317	HTRA2
ENST00000564426	1922	-1.440189616	0.035993237	0.576147864	Down	ENSG00000167964	RAB26
ENST00000417216	1156	1.91453351	0.036112603	0.576147864	Ups	ENSG00000225422	RBMS1P1
ENST00000478077	3036	2.077612673	0.036119575	0.576147864	Ups		
ENST00000395298	372	1.390373922	0.036126601	0.576147864	Ups	ENSG00000213622	AL163952.1
ENST00000592800	1036	2.479097681	0.036155347	0.576147864	Ups	ENSG00000108946	PRKAR1A
ENST00000393034	927	2.83242156	0.036159434	NA	Ups	ENSG00000158234	FAIM
ENST00000534125	527	-3.180444535	0.036161622	NA	Down	ENSG00000137726	FXYD6
ENST00000535618	1363	-3.180444535	0.036161622	NA	Down	ENSG00000215105	TTC3P1
ENST00000561110	529	-3.180444535	0.036161622	NA	Down	ENSG00000104129	DNAJC17
ENST00000507297	3200	-1.953765857	0.036188939	0.576147864	Down	ENSG00000184584	TMEM173
ENST00000524260	1873	-2.740086925	0.036221291	0.576147864	Down	ENSG00000205133	TRIQK
ENST00000252744	5501	-3.185377022	0.036226918	NA	Down	ENSG00000130449	ZSWIM6
ENST00000593400	585	-3.185377022	0.036226918	NA	Down	ENSG00000095059	DHPS
ENST00000530259	906	-3.214125035	0.036269682	NA	Down	ENSG00000173599	PC
ENST00000497289	2137	-1.687784558	0.036277311	0.576147864	Down	ENSG00000163902	RPN1
ENST00000388822	6669	1.297904038	0.036290084	0.576147864	Ups	ENSG00000145388	METTL14
ENST00000371691	2410	-1.066916531	0.03630898	0.576147864	Down	ENSG00000165716	FAM69B
ENST00000264228	4190	2.360269744	0.036313311	NA	Ups	ENSG00000128039	SRD5A3
ENST00000407266	307	2.315334092	0.036339283	0.576147864	Ups	ENSG00000218749	RP3-340B19.5
ENST00000369642	1355	3.205948474	0.036361342	NA	Ups	ENSG00000155366	RHOC
ENST00000585105	5632	-2.233795962	0.03643534	NA	Down	ENSG00000177728	KIAA0195
ENST00000395375	3757	2.260687804	0.036469519	0.576147864	Ups	ENSG00000198039	ZNF273
ENST00000454558	3079	-1.333750645	0.036552102	0.576147864	Down	ENSG00000196843	ARID5A
ENST00000576629	5669	1.103943222	0.036613906	0.576147864	Ups	ENSG00000238083	LRRC37A2
ENST00000564238	3339	1.280744045	0.036638446	0.576147864	Ups	ENSG00000167523	SPATA33
ENST00000527063	4041	1.276239747	0.036673354	0.576147864	Ups	ENSG00000178104	PDE4DIP
ENST00000531778	6160	-2.604760304	0.036726259	0.576147864	Down	ENSG00000109956	B3GAT1
ENST00000492545	634	-1.552242001	0.036731616	0.576147864	Down	ENSG00000144579	CTDSP1
ENST00000569333	4913	1.342981873	0.036778851	0.576147864	Ups	ENSG00000168676	KCTD19
ENST00000433552	3211	3.200070414	0.036781519	NA	Ups		
ENST00000461445	3271	-1.339807419	0.036799892	0.576147864	Down	ENSG00000187091	PLCD1
ENST00000530598	842	2.352732839	0.036801821	NA	Ups	ENSG00000129484	PARP2
ENST00000551328	650	1.966425257	0.036817438	0.576147864	Ups	ENSG00000100519	PSMC6
ENST00000389583	3009	1.32000258	0.036825018	0.576147864	Ups	ENSG00000187783	TMEM72

ENST00000435046	4668	-3.201892179	0.036869268	NA	Down	ENSG00000114867	EIF4G1
ENST00000424893	1279	-1.993456785	0.036908709	0.576147864	Down	ENSG00000172936	MYD88
ENST00000589882	1876	2.167241724	0.036911153	0.576147864	Ups	ENSG00000124731	TREM1
ENST00000493726	733	2.521048137	0.036933631	0.576147864	Ups	ENSG00000186001	LRCH3
ENST00000509389	1379	3.169062318	0.036944241	NA	Ups	ENSG00000117305	HMGCL
ENST00000383263	3043	1.819250257	0.036945255	0.576147864	Ups	ENSG00000184984	CHRM5
ENST00000509409	1248	3.170719027	0.036951589	NA	Ups	ENSG00000143549	TPM3
ENST00000441713	786	-3.1548808	0.037019498	NA	Down	ENSG00000242110	AMACR
ENST00000221264	1610	-2.956283642	0.037052047	NA	Down	ENSG00000011422	PLAUR
ENST00000264192	2260	-3.170772393	0.037154883	NA	Down	ENSG00000115165	CYTIP
ENST00000470541	1877	-3.170772393	0.037154883	NA	Down	ENSG00000116584	ARHGEF2
ENST00000471566	686	3.19192081	0.037155063	NA	Ups	ENSG00000163867	ZMYM6
ENST00000526268	787	-1.510133769	0.03716225	0.577885825	Down	ENSG00000175634	RPS6KB2
ENST00000371378	894	2.181105794	0.037167104	0.577885825	Ups	ENSG00000081870	HSPB11
ENST00000262765	5359	1.597995345	0.037206123	0.577885825	Ups	ENSG00000129646	QRICH2
ENST00000341567	4229	-3.169360213	0.037245559	NA	Down	ENSG00000106609	TMEM248
ENST00000492034	588	-3.169360213	0.037245559	NA	Down	ENSG00000068654	POLR1A
ENST00000552150	1687	-3.169360213	0.037245559	NA	Down	ENSG00000170421	KRT8
ENST00000556500	859	-3.169360213	0.037245559	NA	Down	ENSG00000179454	KLHL28
ENST00000591982	2187	-2.798341751	0.037247839	NA	Down	ENSG00000141854	hsa-mir-1199
ENST00000585962	3027	2.014957189	0.037262923	0.578129941	Ups	ENSG00000141349	G6PC3
ENST00000404701	1207	1.627294854	0.037391187	0.579481044	Ups	ENSG00000217227	RP1-319M7.2
ENST00000514824	1923	3.155173261	0.037500108	NA	Ups	ENSG00000111863	ADTRP
ENST00000431567	3767	2.254147591	0.037514287	NA	Ups	ENSG00000155066	PROM2
ENST00000285046	5720	3.186971381	0.037530655	NA	Ups	ENSG00000154783	FGD5
ENST00000426263	3318	-1.285938116	0.037717835	0.58113416	Down	ENSG00000117394	SLC2A1
ENST00000596215	508	2.401332954	0.037727512	NA	Ups	ENSG00000182986	ZNF320
ENST00000273062	2638	-2.112647827	0.037736107	0.58113416	Down	ENSG00000144579	CTDSP1
ENST00000375856	6999	-2.416509122	0.037747728	0.58113416	Down	ENSG00000185950	IRS2
ENST00000429171	1660	-1.405541879	0.037783071	0.58113416	Down	ENSG00000234814	SVILP1
ENST00000470221	2466	-1.62199574	0.037821734	0.58113416	Down	ENSG00000110619	CARS
ENST00000428282	1005	-3.18573357	0.03782832	NA	Down	ENSG00000168385	43710
ENST00000589477	782	-3.18573357	0.03782832	NA	Down	ENSG00000183401	CCDC159
ENST00000475867	912	3.185686468	0.037836701	NA	Ups	ENSG00000134452	FBXO18
ENST00000530818	486	2.092208719	0.037840564	0.58113416	Ups	ENSG00000179295	PTPN11
ENST00000473467	847	-2.096626056	0.037845421	0.58113416	Down	ENSG00000239779	WBP1
ENST00000491462	1890	-2.334624058	0.037851974	NA	Down	ENSG00000119457	SLC46A2

ENST00000569674	2357	-1.429054785	0.03786953	0.58113416	Down	ENSG00000085491	SLC25A24
ENST00000398733	2702	-3.159170109	0.037901545	NA	Down	ENSG00000131508	UBE2D2
ENST00000493448	1041	-3.159170109	0.037901545	NA	Down	ENSG00000131725	WDR44
ENST00000466999	521	-3.15925509	0.037905294	NA	Down	ENSG00000101365	IDH3B
ENST00000580926	436	1.926002732	0.03792955	0.581421159	Ups	ENSG00000264176	MAGOH2
ENST00000489418	1977	3.141443002	0.037979895	NA	Ups	ENSG00000163950	SLBP
ENST00000489762	1085	-3.137918947	0.038126983	NA	Down		
ENST00000554341	1486	-3.137918947	0.038126983	NA	Down	ENSG00000119723	COQ6
ENST00000348721	2043	-3.162971025	0.038134307	NA	Down	ENSG00000114737	CISH
ENST00000455208	998	-3.162971025	0.038134307	NA	Down	ENSG00000164031	DNAJB14
ENST00000556126	866	-3.185927798	0.038136368	NA	Down	ENSG00000259120	SMIM6
ENST00000604872	970	-3.185927798	0.038136368	NA	Down	ENSG00000109670	FBXW7
ENST00000604465	483	3.154314228	0.038138558	NA	Ups	ENSG00000271164	RP4-789D17.4
ENST00000378940	1126	2.043581834	0.038163171	0.582515373	Ups	ENSG00000165609	NUDT5
ENST00000493497	3240	1.703097089	0.038166679	0.582515373	Ups	ENSG00000131061	ZNF341
ENST00000336440	3026	-1.331790577	0.038177526	0.582515373	Down	ENSG00000185650	ZFP36L1
ENST00000373504	6797	-3.18659866	0.038196872	NA	Down	ENSG00000204116	CHIC1
ENST00000418249	604	-3.18659866	0.038196872	NA	Down	ENSG00000229052	RP11-386I23.1
ENST00000558214	2999	1.122409652	0.03820104	0.582515373	Ups	ENSG00000054690	PLEKHH1
ENST00000438864	766	3.153327653	0.038212163	NA	Ups	ENSG00000233129	RP5-837O21.2
ENST00000322810	15249	-3.184067619	0.03821284	NA	Down	ENSG00000178209	PLEC
ENST00000357641	6052	-3.184067619	0.03821284	NA	Down	ENSG00000096070	BRPF3
ENST00000482370	879	-3.184067619	0.03821284	NA	Down	ENSG00000142676	RPL11
ENST00000471023	592	-2.850127449	0.038255949	NA	Down	ENSG00000101361	NOP56
ENST00000455031	850	1.605231703	0.038268451	0.582515373	Ups	ENSG00000232491	RP4-756H11.1
ENST00000430024	2651	-3.184455093	0.03828542	NA	Down	ENSG00000213297	ZNF625-ZNF20
ENST00000459829	801	-3.184455093	0.03828542	NA	Down	ENSG00000146223	RPL7L1
ENST00000563665	1025	-3.184455093	0.03828542	NA	Down	ENSG00000229809	ZNF688
ENST00000355528	4094	1.899645245	0.038326963	0.582515373	Ups	ENSG00000141556	TBCD
ENST00000543744	2043	-1.317177018	0.038332095	0.582515373	Down	ENSG00000111679	PTPN6
ENST00000596084	2043	-1.317177018	0.038332095	0.582515373	Down		
ENST00000396751	2074	-1.502996734	0.038376027	0.582553884	Down	ENSG00000166333	ILK
ENST00000478158	4614	1.866006259	0.038445143	0.582880119	Ups	ENSG00000169221	TBC1D10B
ENST00000552071	189	3.140366705	0.038505487	NA	Ups		
ENST00000382084	1117	-2.317162734	0.038539847	NA	Down	ENSG00000205853	RFPL3S
ENST00000506558	1333	-1.276700892	0.038542846	0.582880119	Down	ENSG00000229018	RP11-313P13.3
ENST00000600248	1333	-1.276700892	0.038542846	0.582880119	Down		

ENST00000529801	579	2.678068331	0.038582483	NA	Ups	ENSG00000110060	PUS3
ENST00000524391	6979	1.762946159	0.038598168	0.582880119	Ups	ENSG00000164794	KCNV1
ENST00000528288	4094	1.900721433	0.038604624	0.582880119	Ups	ENSG00000182919	C11orf54
ENST00000435198	1056	-3.179389532	0.03868356	NA	Down	ENSG00000160087	UBE2J2
ENST00000461993	856	-3.179389532	0.03868356	NA	Down	ENSG00000131236	CAP1
ENST00000528737	6189	-1.679522992	0.038743843	0.583635685	Down	ENSG00000166801	FAM111A
ENST00000480812	927	-2.290912829	0.038748805	0.583635685	Down	ENSG00000102125	TAZ
ENST0000468518	595	1.218816976	0.038779091	0.583635685	Ups	ENSG00000091136	LAMB1
ENST00000216024	2371	1.437203133	0.038879025	0.584514579	Ups	ENSG00000100206	DMC1
ENST00000589953	664	1.827260541	0.038953986	0.584624113	Ups	ENSG00000075702	WDR62
ENST00000451515	401	1.043569473	0.038969401	0.584624113	Ups	ENSG00000232713	AC010733.5
ENST00000366535	2780	-1.46202828	0.039018064	0.584730778	Down	ENSG00000035687	ADSS
ENST00000600472	312	-2.160635734	0.039044498	NA	Down	ENSG00000269295	AL358113.1
ENST00000507764	838	-3.172339343	0.039093342	NA	Down	ENSG00000109390	NDUFC1
ENST00000584106	1167	-3.172339343	0.039093342	NA	Down	ENSG00000002834	LASP1
ENST00000405858	5051	-3.170532636	0.03909753	NA	Down	ENSG00000164631	ZNF12
ENST00000556272	2942	3.134327803	0.039137501	NA	Ups	ENSG00000100731	PCNX
ENST00000561381	979	3.139341349	0.039145267	NA	Ups	ENSG00000140455	USP3
ENST00000398664	2026	-3.138888984	0.039243704	NA	Down	ENSG00000205456	TP53TG3D
ENST00000468016	981	-3.166896659	0.039307209	NA	Down	ENSG00000160202	CRYAA
ENST00000477581	549	-3.166896659	0.039307209	NA	Down	ENSG00000213047	DENND1B
ENST00000506919	1687	-3.166896659	0.039307209	NA	Down	ENSG00000151611	MMAA
ENST00000483582	2467	-1.788571825	0.039341967	0.588331737	Down	ENSG00000163702	IL17RC
ENST00000463221	1635	2.615281082	0.039381379	NA	Ups	ENSG00000196562	SULF2
ENST00000378927	1258	1.813338394	0.039416695	0.588728534	Ups	ENSG00000165609	NUDT5
ENST00000578049	1874	-2.621326361	0.03943617	NA	Down		
ENST00000468435	3723	-3.164012483	0.039443585	NA	Down	ENSG00000147854	UHRF2
ENST00000577433	803	-3.164012483	0.039443585	NA	Down	ENSG00000072778	ACADVL
ENST00000282074	1354	1.725435287	0.039472474	0.588728534	Ups	ENSG00000152253	SPC25
ENST00000481729	634	1.29560681	0.039494012	0.588728534	Ups	ENSG00000135968	GCC2
ENST00000551834	562	3.129428386	0.039498619	NA	Ups	ENSG00000196465	MYL6B
ENST00000409869	2968	3.125610999	0.039506815	NA	Ups	ENSG00000075884	ARHGAP15
ENST00000558629	4374	3.163900776	0.039557531	NA	Ups	ENSG00000128829	EIF2AK4
ENST00000328902	3440	2.63088185	0.039584079	NA	Ups	ENSG00000133561	GIMAP6
ENST00000379868	4089	3.158118787	0.039600533	NA	Ups	ENSG00000107201	DDX58
ENST00000588215	1263	1.767044766	0.039638071	0.5895147	Ups	ENSG00000236252	RP11-15J10.8
ENST00000417905	427	-2.164435248	0.039647482	0.5895147	Down	ENSG00000003756	RBM5

ENST00000337554	881	-3.162282231	0.039700628	NA	Down	ENSG00000100300	TSPO
ENST00000532586	839	-1.568474717	0.039707262	0.5895147	Down	ENSG00000185475	TMEM179B
ENST00000409993	3274	-1.335333939	0.03974755	0.5895147	Down	ENSG00000172269	DPAGT1
ENST00000354484	4068	1.527425094	0.039756215	0.5895147	Ups	ENSG00000197859	ADAMTSL2
ENST00000492304	685	-2.367863756	0.039775324	NA	Down	ENSG00000023902	PLEKHO1
ENST00000523708	376	3.098620287	0.039832634	NA	Ups	ENSG00000253543	RP11-89K10.2
ENST00000491782	2078	2.49420421	0.039868228	0.589707559	Ups	ENSG00000011426	ANLN
ENST00000511125	3293	2.458385495	0.03988621	0.589707559	Ups	ENSG00000170365	SMAD1
ENST00000311371	2716	-2.305733936	0.03989494	0.589707559	Down	ENSG00000039600	SOX30
ENST00000580285	2681	3.153546299	0.039900457	NA	Ups	ENSG00000258890	CEP95
ENST00000419835	2720	-3.127965084	0.039981733	NA	Down	ENSG00000112118	MCM3
ENST00000325954	3825	-1.777703217	0.04002199	0.590497089	Down	ENSG00000022840	RNF10
ENST00000469402	321	3.113361804	0.04003997	NA	Ups	ENSG00000240436	RP11-95I19.1
ENST00000592192	412	2.067182753	0.040044013	0.590497089	Ups	ENSG00000187775	DNAH17
ENST00000497864	802	-1.472704626	0.040138362	0.590497089	Down	ENSG00000248487	ABHD14A
ENST00000330149	3242	-3.151318031	0.040247078	NA	Down	ENSG00000182400	TRAPPC6B
ENST00000420986	2322	-3.151318031	0.040247078	NA	Down	ENSG00000150776	C11orf57
ENST00000577170	2322	-3.151318031	0.040247078	NA	Down		
ENST00000265720	2217	-3.124056713	0.040248185	NA	Down	ENSG00000105865	DUS4L
ENST00000481435	627	-3.124056713	0.040248185	NA	Down	ENSG00000008952	SEC62
ENST00000369947	1543	-3.131087175	0.040281726	NA	Down	ENSG00000135312	HTR1B
ENST00000495031	2214	-3.131087175	0.040281726	NA	Down	ENSG00000175984	DENND2C
ENST00000571982	1840	3.110546239	0.040306704	NA	Ups		
ENST00000352456	3426	-1.388271223	0.040313698	0.590497089	Down	ENSG00000105323	HNRNPUL1
ENST00000358739	531	-3.158971362	0.040338295	NA	Down	ENSG00000196747	HIST1H2AI
ENST00000351217	2817	-3.153954273	0.040346188	NA	Down	ENSG00000156642	NPTN
ENST00000546922	892	-3.153954273	0.040346188	NA	Down	ENSG00000135452	TSPAN31
ENST00000494188	771	1.348362379	0.04038892	0.590497089	Ups	ENSG00000138069	RAB1A
ENST00000398421	2354	-1.82895346	0.040396934	0.590497089	Down	ENSG00000158517	NCF1
ENST00000595467	1783	-2.231442177	0.040397143	0.590497089	Down	ENSG00000105677	TMEM147
ENST00000464576	393	-2.647039364	0.040413325	NA	Down	ENSG00000072135	PTPN18
ENST00000412658	2091	3.148414714	0.040447259	NA	Ups	ENSG00000100031	GGT1
ENST00000260447	775	-3.150035928	0.040502941	NA	Down	ENSG00000137880	GCHFR
ENST00000495884	574	3.119075512	0.040531609	NA	Ups	ENSG00000240870	RPL19P14
ENST00000590227	3269	1.524776279	0.040588157	0.590497089	Ups	ENSG00000187775	DNAH17
ENST00000549100	501	-3.11888406	0.040601008	NA	Down	ENSG00000166153	DEPDC4
ENST00000529844	1781	3.110831348	0.040602772	NA	Ups	ENSG00000254469	RP11-849H4.2

ENST00000555253	3549	1.356664177	0.040606382	0.590497089	Ups	ENSG00000119630	PGF
ENST00000475454	366	-2.117926087	0.040627215	0.590497089	Down	ENSG00000164867	NOS3
ENST00000525470	922	2.027923739	0.04062811	0.590497089	Ups	ENSG00000166902	MRPL16
ENST00000524119	5513	1.941138895	0.040648141	0.590497089	Ups	ENSG00000136986	DERL1
ENST00000380133	903	-3.150248449	0.040651275	NA	Down	ENSG00000139597	N4BP2L1
ENST00000498154	535	-3.150248449	0.040651275	NA	Down	ENSG00000114861	FOXP1
ENST00000555644	1347	-3.150248449	0.040651275	NA	Down	ENSG00000092020	PPP2R3C
ENST00000258418	3826	1.202789055	0.040668734	0.590497089	Ups	ENSG00000135932	CAB39
ENST00000506842	706	1.879676892	0.040747866	0.590497089	Ups	ENSG00000138642	HERC6
ENST00000483404	3082	1.680760796	0.04076575	0.590497089	Ups	ENSG00000059588	TARBP1
ENST00000437446	462	-3.147773623	0.040773012	NA	Down	ENSG00000239665	RP11-295P9.3
ENST00000480951	814	-3.147773623	0.040773012	NA	Down	ENSG00000145919	BOD1
ENST00000508854	1134	-3.147773623	0.040773012	NA	Down	ENSG00000214193	SH3D21
ENST00000557201	1371	-3.147773623	0.040773012	NA	Down	ENSG00000092199	HNRNPC
ENST00000591584	667	-3.147773623	0.040773012	NA	Down	ENSG00000131475	VPS25
ENST00000361007	4734	2.377573278	0.040787605	0.590497089	Ups	ENSG00000197879	MYO1C
ENST00000576839	5963	3.103285401	0.040814489	NA	Ups		
ENST00000601440	5963	3.103285401	0.040814489	NA	Ups	ENSG00000188171	ZNF626
ENST00000380250	10735	-2.174540934	0.04084012	0.590649716	Down	ENSG00000073910	FRY
ENST00000580303	637	3.100115191	0.040959573	NA	Ups	ENSG00000185158	LRRC37B
ENST00000491571	893	3.087505097	0.040989849	NA	Ups		
ENST00000445082	1107	2.407032405	0.041011246	NA	Ups	ENSG00000186162	CIDECP
ENST00000255608	2635	3.137312781	0.041028237	NA	Ups	ENSG00000133243	BTBD2
ENST00000572944	5179	2.443571567	0.041099611	0.593253804	Ups	ENSG00000153933	DGKE
ENST00000486155	2989	1.295936431	0.041104495	0.593253804	Ups	ENSG00000112053	SLC26A8
ENST00000477456	422	3.110313394	0.0411465	NA	Ups	ENSG00000229314	ORM1
ENST00000528617	532	-3.139494509	0.041229223	NA	Down	ENSG00000174915	PTDSS2
ENST00000389398	4600	-3.109123188	0.041268607	NA	Down	ENSG00000175267	VWA3A
ENST00000333244	18254	-1.252222787	0.041354012	0.596243496	Down	ENSG00000185567	AHNAK2
ENST00000246553	923	3.137601702	0.041399814	NA	Ups	ENSG00000126266	FFAR1
ENST00000370253	3889	3.132490643	0.041432949	NA	Ups	ENSG00000137942	FNBP1L
ENST00000422800	570	-2.70082255	0.041463597	NA	Down	ENSG00000106052	TAX1BP1
ENST00000323777	4094	1.755445285	0.041472763	0.596943138	Ups	ENSG00000176160	HSF5
ENST00000397809	3773	1.663716934	0.041507034	0.596943138	Ups	ENSG00000173588	CCDC41
ENST00000574518	4707	2.760346146	0.041580934	NA	Ups	ENSG00000183914	DNAH2
ENST00000517372	573	-3.104224745	0.041606815	NA	Down	ENSG00000186918	ZNF395
ENST00000444739	2158	-1.682921906	0.041653152	0.596943138	Down	ENSG00000196954	CASP4

ENST00000395468	6480	1.064152558	0.041671924	0.596943138	Ups	ENSG00000168175	MAPK1IP1L
ENST00000417533	625	-1.886853362	0.041703945	0.596943138	Down	ENSG00000229422	RP11-262H14.5
ENST00000357967	3499	-3.131668619	0.041719334	NA	Down	ENSG00000196678	ERI2
ENST00000473675	783	-3.131668619	0.041719334	NA	Down	ENSG00000173890	GPR160
ENST00000423513	1382	1.250367342	0.0417669	0.596943138	Ups	ENSG00000116044	NFE2L2
ENST00000562865	5683	2.9140352	0.041784323	0.596943138	Ups		
ENST00000474872	769	-2.159288103	0.041795811	NA	Down	ENSG00000134321	RSAD2
ENST00000450808	950	-3.131222912	0.041821181	NA	Down	ENSG00000162368	CMPK1
ENST00000552212	1497	-3.131222912	0.041821181	NA	Down	ENSG00000181929	PRKAG1
ENST00000238570	12703	1.604632299	0.041844993	0.597203589	Ups	ENSG00000100731	PCNX
ENST00000532805	2325	1.262513728	0.041953108	0.59734052	Ups	ENSG00000186104	CYP2R1
ENST00000570468	2325	1.262513728	0.041953108	0.59734052	Ups		
ENST00000372564	2982	-1.674280458	0.042029966	0.59734052	Down	ENSG00000148341	SH3GLB2
ENST00000473202	5989	1.165412514	0.042123997	0.59734052	Ups	ENSG00000135919	SERPINE2
ENST00000299980	6959	2.635230702	0.042124985	0.59734052	Ups	ENSG00000166747	AP1G1
ENST00000574662	1755	-1.717725636	0.042164461	0.59734052	Down	ENSG00000103056	SMPD3
ENST00000268489	16064	3.120978956	0.04217951	NA	Ups	ENSG00000140836	ZFHX3
ENST00000374283	1973	2.043984823	0.042191656	0.59734052	Ups	ENSG00000165181	C9orf84
ENST00000411546	2362	-1.59083866	0.042194178	0.59734052	Down	ENSG00000183604	RP11-347C12.2
ENST00000585090	614	2.714843703	0.04220708	NA	Ups	ENSG00000101608	MYL12A
ENST00000302909	6162	3.088786233	0.04223343	NA	Ups	ENSG00000171303	KCNK3
ENST00000493837	3769	3.088786233	0.04223343	NA	Ups	ENSG00000117834	SLC5A9
ENST00000561656	1833	-3.132594383	0.042250612	NA	Down	ENSG00000067221	STOML1
ENST00000518259	6871	1.237029713	0.042309022	0.59829016	Ups	ENSG00000197140	ADAM32
ENST00000268459	4592	1.735619967	0.04234629	0.59829016	Ups	ENSG00000140807	NKD1
ENST00000553155	2092	1.15906382	0.042534081	0.599739066	Ups		
ENST00000268661	2182	3.059771384	0.042619896	NA	Ups	ENSG00000140986	RPL3L
ENST00000489793	528	3.059771384	0.042619896	NA	Ups	ENSG00000151690	MFSD6
ENST00000512202	600	3.059771384	0.042619896	NA	Ups	ENSG00000048342	CC2D2A
ENST00000591762	2502	-1.888296856	0.042643544	0.600680635	Down	ENSG00000176170	SPHK1
ENST00000262584	1041	1.903800919	0.042740059	0.600708047	Ups	ENSG00000161016	RPL8
ENST00000334211	4942	-2.916190542	0.042740275	NA	Down	ENSG00000186635	ARAP1
ENST00000505458	3707	-2.375304732	0.042776368	0.600708047	Down	ENSG00000109320	NFKB1
ENST00000482915	665	3.087444848	0.042787359	NA	Ups	ENSG00000154642	C21orf91
ENST00000497134	1873	3.087444848	0.042787359	NA	Ups	ENSG00000151694	ADAM17
ENST00000296328	10568	2.579390591	0.042794632	NA	Ups	ENSG00000163960	UBXN7
ENST00000518743	568	1.411792329	0.042816243	0.600708047	Ups	ENSG00000253770	HMGB1P23

ENST00000604796	643	-2.457227975	0.042888728	NA	Down	ENSG00000271642	RP11-66E20.2
ENST00000532104	1912	1.933647401	0.042916564	0.601248647	Ups	ENSG00000123201	GUCY1B2
ENST00000257248	1518	3.084542393	0.04296173	NA	Ups	ENSG00000134812	GIF
ENST00000418299	1009	3.084542393	0.04296173	NA	Ups	ENSG00000155542	SETD9
ENST00000219022	2897	-3.117495075	0.043043886	NA	Down	ENSG00000102837	OLFM4
ENST00000463646	2406	-3.117495075	0.043043886	NA	Down	ENSG00000165125	TRPV6
ENST00000540348	2866	1.737224937	0.043049464	0.601248647	Ups	ENSG00000165006	UBAP1
ENST00000411827	597	-3.090678732	0.043057089	NA	Down	ENSG00000070831	CDC42
ENST00000460040	448	-3.090678732	0.043057089	NA	Down	ENSG00000136059	VILL
ENST00000539528	839	-3.090678732	0.043057089	NA	Down	ENSG00000177981	ASB8
ENST00000592399	570	2.623287866	0.043136379	NA	Ups	ENSG00000167895	TMC8
ENST00000392580	3522	-2.50552405	0.043151205	0.601248647	Down	ENSG00000109956	B3GAT1
ENST00000414286	515	-3.110049075	0.043156069	NA	Down	ENSG00000215183	MSMP
ENST00000443375	4322	-3.110049075	0.043156069	NA	Down	ENSG00000146067	FAM193B
ENST00000552056	1981	-3.110049075	0.043156069	NA	Down	ENSG00000187109	NAP1L1
ENST00000404582	967	1.177953507	0.043169167	0.601248647	Ups	ENSG00000218283	MORF4L1P1
ENST00000541657	1626	-1.260525851	0.043175443	0.601248647	Down	ENSG00000139725	RHOF
ENST00000466317	2684	1.240003925	0.043198307	0.601248647	Ups	ENSG00000154803	FLCN
ENST00000507956	1300	2.192731673	0.043254962	0.601248647	Ups	ENSG00000138744	NAAA
ENST00000586054	2832	2.276046442	0.043320624	0.601248647	Ups	ENSG00000104904	OAZ1
ENST00000530455	2676	1.046501632	0.043321114	0.601248647	Ups	ENSG00000182307	C8orf33
ENST00000473911	732	-1.919971777	0.043335693	0.601248647	Down	ENSG00000169583	CLIC3
ENST00000550079	565	1.64048698	0.04340409	0.601248647	Ups	ENSG00000139624	CERS5
ENST00000468316	2045	-1.739935925	0.043410221	0.601248647	Down	ENSG00000128563	PRKRIP1
ENST00000567878	2045	-1.739935925	0.043410221	0.601248647	Down		
ENST00000498090	1673	3.072567713	0.043502922	NA	Ups	ENSG00000144218	AFF3
ENST00000555679	606	2.522205068	0.043517077	NA	Ups	ENSG00000100764	PSMC1
ENST00000438364	3947	-3.106265824	0.043549761	NA	Down	ENSG00000170629	DPY19L2P2
ENST00000369175	1243	2.323909481	0.043627271	0.602126713	Ups	ENSG00000203817	FAM72C
ENST00000452656	2763	2.530055412	0.043636693	0.602126713	Ups	ENSG00000184792	OSBP2
ENST00000547729	775	1.553591221	0.043659004	0.602126713	Ups	ENSG00000257376	RP11-328C8.2
ENST00000347970	1329	-2.638764776	0.043675257	NA	Down	ENSG00000184277	TM2D3
ENST00000525750	656	-1.08918961	0.043684913	0.602126713	Down	ENSG00000185507	IRF7
ENST00000549938	696	-1.976669463	0.043723028	0.602126713	Down	ENSG00000120802	TMPO
ENST00000270162	4712	-1.698314807	0.043730351	0.602126713	Down	ENSG00000142178	SIK1
ENST00000394235	4012	3.109502972	0.043746845	NA	Ups	ENSG00000109381	ELF2
ENST00000562496	583	2.71885619	0.043755415	NA	Ups	ENSG00000102900	NUP93

ENST00000407464	6798	3.048174239	0.043835753	NA	Ups	ENSG00000169635	HIC2
ENST00000360091	1289	-2.645063526	0.043838809	NA	Down	ENSG00000182944	EWSR1
ENST00000401695	1599	1.231794317	0.043844904	0.602524888	Ups	ENSG00000164587	RPS14
ENST00000369014	2085	1.53763371	0.043917664	0.602916749	Ups	ENSG00000143420	ENSA
ENST00000530854	730	-2.674876308	0.043965427	NA	Down	ENSG00000160959	LRRC14
ENST00000288135	5186	3.073393586	0.04397965	NA	Ups	ENSG00000157404	KIT
ENST00000394239	1680	3.073393586	0.04397965	NA	Ups	ENSG00000108423	TUBD1
ENST00000467471	2121	-2.582382227	0.044002033	NA	Down	ENSG00000164088	PPM1M
ENST00000520094	581	2.241919015	0.044017477	NA	Ups	ENSG00000170873	MTSS1
ENST00000310528	14131	1.34112662	0.044051963	0.602916749	Ups	ENSG00000165416	SUGT1
ENST00000367095	1779	-1.036295343	0.044052793	0.602916749	Down	ENSG00000162892	IL24
ENST00000501107	704	1.243181538	0.044061194	0.602916749	Ups	ENSG00000246575	AC093162.5
ENST00000542713	7384	1.549233677	0.044087645	0.602916749	Ups	ENSG00000096060	FKBP5
ENST00000512551	623	-2.696904203	0.044129811	NA	Down	ENSG00000213551	DNAJC9
ENST00000461918	553	2.73480879	0.044143815	NA	Ups	ENSG00000084234	APLP2
ENST00000476508	945	2.072632793	0.04416944	0.603448893	Ups	ENSG00000142856	ITGB3BP
ENST00000512214	2912	2.763500942	0.044223064	NA	Ups	ENSG00000145416	43525
ENST00000475010	3467	3.094275466	0.044255208	NA	Ups	ENSG00000133606	MKRN1
ENST00000522484	3646	3.094275466	0.044255208	NA	Ups	ENSG00000164930	FZD6
ENST00000501501	4158	-3.094423353	0.04430123	NA	Down	ENSG00000108819	PPP1R9B
ENST00000566540	1557	3.089357301	0.044308078	NA	Ups	ENSG00000140650	PMM2
ENST00000311507	1447	1.443918005	0.04430893	0.604767468	Ups	ENSG00000145287	PLAC8
ENST00000221899	2861	-3.073371101	0.044317149	NA	Down	ENSG00000105298	CACTIN
ENST00000517904	661	3.061225503	0.044345388	NA	Ups	ENSG00000044115	CTNNA1
ENST00000593461	640	3.040619386	0.044401263	NA	Ups	ENSG00000188051	TMEM221
ENST00000548065	2071	-2.329695216	0.044407908	0.605043348	Down	ENSG00000181929	PRKAG1
ENST00000369238	6926	1.768265145	0.04447667	0.605043348	Ups	ENSG00000162836	ACP6
ENST00000252711	4915	3.089858532	0.044691523	NA	Ups	ENSG00000130414	NDUFA10
ENST00000336278	9789	1.883180641	0.044744168	0.605043348	Ups	ENSG00000168807	SNTB2
ENST00000331459	939	2.554854198	0.044755706	NA	Ups	ENSG00000184166	OR1D2
ENST00000536224	1805	-1.615027664	0.044810866	0.605043348	Down	ENSG00000131979	GCH1
ENST00000601309	672	1.287077177	0.044906467	0.605043348	Ups	ENSG00000105323	HNRNPUL1
ENST00000508249	604	-2.886306539	0.045060841	NA	Down	ENSG00000109332	UBE2D3
ENST00000379638	3318	1.540785448	0.045095373	0.605043348	Ups	ENSG00000129625	REEP5
ENST00000326279	4008	1.910919564	0.045115357	0.605043348	Ups	ENSG00000131914	LIN28A
ENST00000380075	1967	-3.062560519	0.045125116	NA	Down	ENSG00000138100	TRIM54
ENST00000377307	4673	-1.813418786	0.045142568	0.605043348	Down	ENSG00000147799	ARHGAP39

ENST00000541204	2947	1.952004519	0.045169797	0.605043348	Ups	ENSG00000063978	RNF4
ENST00000472925	983	-2.141995856	0.045223666	0.605043348	Down	ENSG00000041988	THAP3
ENST00000457611	483	3.048201721	0.045284627	NA	Ups	ENSG00000227649	RP11-522L3.9
ENST00000547570	624	-2.222842784	0.045292908	0.605043348	Down	ENSG00000257727	CNPY2
ENST00000469042	3276	-3.064575603	0.045315056	NA	Down	ENSG00000240303	ACAD11
ENST00000341162	2412	1.350131135	0.045345034	0.605043348	Ups	ENSG00000119616	FCF1
ENST00000356421	8118	2.20542455	0.045358331	NA	Ups	ENSG00000144218	AFF3
ENST00000368801	9623	1.318885455	0.045366025	0.605043348	Ups	ENSG00000197915	HRNR
ENST00000517561	498	2.720707115	0.045380059	NA	Ups	ENSG00000132842	AP3B1
ENST00000392563	2851	3.081850415	0.045434244	NA	Ups	ENSG00000177885	GRB2
ENST00000312828	9501	2.590272938	0.045498814	NA	Ups	ENSG00000176641	RNF152
ENST00000423648	497	2.017723906	0.045508467	0.605043348	Ups	ENSG00000214243	AC004980.10
ENST00000448083	1427	-1.596756849	0.045557446	0.605043348	Down	ENSG00000175592	FOSL1
ENST00000582452	479	1.414391832	0.045566217	0.605043348	Ups	ENSG00000263812	LINC00908
ENST00000589857	575	-2.338173674	0.045571308	0.605043348	Down	ENSG00000171817	ZNF540
ENST00000380929	1695	-3.056550458	0.045577283	NA	Down	ENSG00000101850	GPR143
ENST00000461417	545	-3.056550458	0.045577283	NA	Down	ENSG00000115661	STK16
ENST00000290231	3220	-2.685216398	0.045578682	NA	Down	ENSG00000124160	NCOA5
ENST00000433570	3117	1.465798253	0.045619789	0.605043348	Ups	ENSG00000155380	SLC16A1
ENST00000560407	468	2.346641525	0.045695266	NA	Ups	ENSG00000067141	NEO1
ENST00000436137	2272	1.287560641	0.045729228	0.605043348	Ups	ENSG00000110013	SIAE
ENST00000298569	2991	3.07102234	0.045732605	NA	Ups	ENSG00000122203	KIAA1191
ENST00000305533	1718	-1.97550426	0.045744904	0.605043348	Down	ENSG00000247596	TWF2
ENST00000587757	2171	-2.390911749	0.0457463	0.605043348	Down	ENSG00000168675	LDLRAD4
ENST00000558989	1816	1.539201839	0.045767228	0.605043348	Ups	ENSG00000104133	SPG11
ENST00000262293	2332	1.907466227	0.045779611	0.605043348	Ups	ENSG00000068489	PRR11
ENST00000306090	1642	3.073850525	0.045784313	NA	Ups	ENSG00000087460	GNAS
ENST00000449001	3111	3.037960761	0.045784514	NA	Ups	ENSG00000008323	PLEKHG6
ENST00000606848	1787	3.037960761	0.045784514	NA	Ups	ENSG00000230596	AC112229.4
ENST00000264917	5956	-1.640195041	0.045825647	0.605043348	Down	ENSG00000113231	PDE8B
ENST00000488156	732	-1.049151654	0.045830854	0.605043348	Down	ENSG00000168374	ARF4
ENST00000456523	5234	1.59599084	0.045849774	0.605043348	Ups	ENSG00000138675	FGF5
ENST00000418871	4219	2.333524887	0.045882529	0.605043348	Ups	ENSG00000170949	ZNF160
ENST00000421129	336	1.490243064	0.045885321	0.605043348	Ups	ENSG00000237748	UQCRBP1
ENST00000508624	7406	3.08041823	0.04589756	NA	Ups	ENSG00000134982	APC
ENST00000478047	2694	1.546468444	0.045918807	0.605043348	Ups	ENSG00000095485	CWF19L1
ENST00000533904	2212	2.639010654	0.045964248	0.605043348	Ups	ENSG00000134940	ACRV1

ENST00000570063	1821	-2.197939552	0.045973454	NA	Down	ENSG00000117616	C1orf63
ENST00000327277	3348	3.070652588	0.045976894	NA	Ups	ENSG00000155229	MMS19
ENST00000453453	1638	3.070652588	0.045976894	NA	Ups	ENSG00000115234	SNX17
ENST00000533572	975	-2.022364327	0.045995838	0.605043348	Down	ENSG00000164733	CTSB
ENST00000569700	1156	2.389450308	0.046004381	NA	Ups	ENSG00000063854	HAGH
ENST00000490290	841	-2.495412193	0.046028402	NA	Down	ENSG00000163870	TPRA1
ENST00000565981	1959	3.038101334	0.046054272	NA	Ups	ENSG00000103034	NDRG4
ENST00000343702	3615	-3.073307062	0.046056581	NA	Down	ENSG00000074527	NTN4
ENST00000434319	1515	-3.073307062	0.046056581	NA	Down	ENSG00000172322	CLEC12A
ENST00000476503	1006	-3.073307062	0.046056581	NA	Down	ENSG00000171163	ZNF692
ENST00000425986	1973	3.066157274	0.04606163	NA	Ups	ENSG00000040608	RTN4R
ENST00000456936	3757	3.045429053	0.04608832	NA	Ups	ENSG00000134970	TMED7
ENST00000316105	4217	2.034899437	0.046109879	0.605043348	Ups	ENSG00000177839	PCDHB9
ENST00000609989	4217	2.034899437	0.046109879	0.605043348	Ups		
ENST00000561278	578	-1.909659061	0.046142047	0.605043348	Down	ENSG00000138606	SHF
ENST00000409028	1110	2.184827893	0.04614618	0.605043348	Ups	ENSG00000071082	RPL31
ENST00000513162	4250	-1.051031925	0.046206028	0.605043348	Down	ENSG00000145916	RMND5B
ENST00000593704	569	1.894050272	0.046206873	0.605043348	Ups	ENSG00000012124	CD22
ENST00000549548	1698	1.177940307	0.046256291	0.605043348	Ups	ENSG00000135407	AVIL
ENST00000473414	3706	1.69262358	0.046282325	0.605043348	Ups	ENSG00000114744	COMMD2
ENST00000596009	3130	1.441668543	0.046305158	0.605043348	Ups	ENSG00000268949	MRPS17P1
ENST00000261182	5202	1.811929894	0.046338304	0.605043348	Ups	ENSG00000187109	NAP1L1
ENST00000234668	3000	2.049974401	0.046362999	0.605043348	Ups	ENSG00000097096	SYDE2
ENST00000578824	720	-1.184814512	0.046380392	0.605043348	Down	ENSG00000072778	ACADVL
ENST00000395855	1862	3.063816101	0.046401577	NA	Ups	ENSG00000196338	NLGN3
ENST00000586458	2674	1.096056595	0.046401899	0.605043348	Ups	ENSG00000186020	ZNF529
ENST00000477930	901	-3.075054323	0.046450843	NA	Down	ENSG00000146232	NFKBIE
ENST00000253699	6678	1.551422628	0.046474617	0.605043348	Ups	ENSG00000131381	ZFYVE20
ENST00000467610	1366	-1.224963266	0.046483768	0.605043348	Down	ENSG00000134107	BHLHE40
ENST00000476417	6945	1.934183386	0.046518326	0.605043348	Ups	ENSG00000123243	ITIH5
ENST00000580261	669	1.374072481	0.046547176	0.605043348	Ups	ENSG00000265681	RPL17
ENST00000393276	5701	1.5778481	0.046640082	0.605043348	Ups	ENSG00000175984	DENND2C
ENST00000395354	1572	-3.046843869	0.04666248	NA	Down	ENSG00000113368	LMNB1
ENST00000466568	466	3.057505624	0.046681285	NA	Ups	ENSG00000214655	ZSWIM8
ENST00000587350	361	1.965932474	0.046703117	0.605043348	Ups	ENSG00000141150	RASL10B
ENST00000550321	1646	2.438071496	0.046710261	0.605043348	Ups	ENSG00000258363	GRAMD4P4
ENST00000377751	1430	-3.041961172	0.046732669	NA	Down	ENSG00000197043	ANXA6

ENST00000176763	6060	-1.408178789	0.046734128	0.605043348	Down	ENSG00000072786	STK10
ENST00000375354	543	3.034104734	0.046748443	NA	Ups	ENSG00000175787	ZNF169
ENST00000462590	1026	-2.268772524	0.046789686	NA	Down	ENSG00000196924	FLNA
ENST00000599010	1026	-2.268772524	0.046789686	NA	Down		
ENST00000248553	909	-1.922011332	0.046847191	0.605043348	Down	ENSG00000106211	HSPB1
ENST00000346141	1490	1.122950414	0.046852621	0.605043348	Ups	ENSG00000108423	TUBD1
ENST00000577240	743	1.35521576	0.046887809	0.605043348	Ups	ENSG00000125457	MIF4GD
ENST00000559323	2002	2.40898	0.046899679	NA	Ups	ENSG00000181991	MRPS11
ENST00000357647	469	-2.039046186	0.046910954	0.605043348	Down	ENSG00000198366	HIST1H3A
ENST00000437626	3977	-3.068807772	0.046931737	NA	Down	ENSG00000197381	ADARB1
ENST00000605140	1244	1.620457134	0.047006851	0.605043348	Ups		
ENST00000377707	2783	3.005767273	0.047042689	NA	Ups	ENSG00000107338	SHB
ENST00000480316	786	3.005767273	0.047042689	NA	Ups	ENSG00000154803	FLCN
ENST00000587604	513	3.005767273	0.047042689	NA	Ups	ENSG00000130816	DNMT1
ENST00000325203	5416	2.01023806	0.047058095	0.605043348	Ups	ENSG00000091879	ANGPT2
ENST00000418513	2341	-2.38942814	0.047112413	0.605043348	Down	ENSG00000172985	SH3RF3
ENST00000409887	3157	-1.245454915	0.047129598	0.605043348	Down	ENSG00000181350	FAM211A
ENST00000437429	1541	3.054443333	0.047134246	NA	Ups	ENSG00000168679	SLC16A4
ENST00000332674	3470	1.478700318	0.047135619	0.605043348	Ups	ENSG00000119950	MXI1
ENST00000306984	7668	1.025873122	0.047166045	0.605043348	Ups	ENSG00000168303	MPLKIP
ENST00000603716	632	1.802875314	0.047271393	0.605043348	Ups	ENSG00000271278	TCEB1P33
ENST00000532917	2678	2.535558942	0.047297674	NA	Ups	ENSG00000160654	CD3G
ENST00000393216	1638	3.002178629	0.047313163	NA	Ups	ENSG00000188523	C9orf171
ENST00000467939	552	3.002178629	0.047313163	NA	Ups	ENSG00000167614	TTYH1
ENST00000571065	552	3.002178629	0.047313163	NA	Ups		
ENST00000575109	552	3.002178629	0.047313163	NA	Ups		
ENST00000399022	4438	1.598204684	0.047335929	0.605043348	Ups	ENSG00000141425	RPRD1A
ENST00000408957	2448	1.462088505	0.047339839	0.605043348	Ups	ENSG00000221944	TIGD1
ENST00000292823	5597	1.328514016	0.047393654	0.605043348	Ups	ENSG00000161217	PCYT1A
ENST00000576379	1716	1.850568201	0.047404571	0.605043348	Ups		
ENST00000424606	642	1.349748215	0.047448907	0.605043348	Ups	ENSG00000236745	YRDCP2
ENST00000258662	2098	1.000463099	0.047455453	0.605043348	Ups	ENSG00000136159	NUDT15
ENST00000336470	2357	2.063588048	0.047467869	0.605043348	Ups	ENSG00000130119	GNL3L
ENST00000343304	3190	3.049680437	0.04760024	NA	Ups	ENSG00000132128	LRRC41
ENST00000394894	3201	3.049680437	0.04760024	NA	Ups	ENSG00000112984	KIF20A
ENST00000377276	1571	-3.034265348	0.047640109	NA	Down	ENSG00000165059	PRKACG
ENST00000526020	1512	-1.210218173	0.047669117	0.607058654	Down	ENSG00000236287	ZBED5

ENST00000371671	1603	-3.02870868	0.047699436	NA	Down	ENSG00000196642	RABL6
ENST00000446365	2570	-3.02870868	0.047699436	NA	Down	ENSG00000004897	CDC27
ENST00000533415	2570	-3.02870868	0.047699436	NA	Down	ENSG00000004897	CDC27
ENST00000551757	1151	1.040545574	0.047783011	0.607106835	Ups	ENSG00000139624	CERS5
ENST00000502687	883	3.016915356	0.047839466	NA	Ups	ENSG00000163697	APBB2
ENST00000433891	527	1.256306907	0.047841236	0.607106835	Ups	ENSG00000108292	MLLT6
ENST00000266037	8755	-1.593679577	0.047871633	0.607106835	Down	ENSG00000088538	DOCK3
ENST00000554206	1420	3.018407666	0.047878045	NA	Ups	ENSG00000100784	RPS6KA5
ENST00000605193	268	3.012000278	0.047896058	NA	Ups	ENSG00000270531	RP11-169K17.2
ENST00000466486	861	3.021500431	0.047969449	NA	Ups	ENSG00000123560	PLP1
ENST00000337995	3535	3.018540184	0.047972797	NA	Ups	ENSG00000197050	ZNF420
ENST00000473805	1013	3.018540184	0.047972797	NA	Ups	ENSG00000078618	NRD1
ENST00000409174	5278	3.046124453	0.047996182	NA	Ups	ENSG00000159399	HK2
ENST00000470655	5003	-1.279631944	0.048012234	0.607106835	Down	ENSG00000133466	C1QTNF6
ENST00000492479	866	-1.593860997	0.048029242	0.607106835	Down	ENSG00000132334	PTPRE
ENST00000559759	2994	1.181037106	0.048033065	0.607106835	Ups	ENSG00000100767	PAPLN
ENST00000216727	2001	1.273967054	0.048054494	0.607106835	Ups	ENSG00000100836	PABPN1
ENST00000523267	741	-3.050882845	0.048056554	NA	Down	ENSG00000161010	C5orf45
ENST00000538666	1168	-3.050882845	0.048056554	NA	Down	ENSG00000127318	IL22
ENST00000338352	1689	1.479344434	0.048094493	0.607106835	Ups	ENSG00000189401	OTUD6A
ENST00000296214	1525	-3.02305313	0.048140424	NA	Down	ENSG00000163875	MEAF6
ENST00000444778	551	-3.02305313	0.048140424	NA	Down	ENSG00000100297	MCM5
ENST00000254719	4340	-3.049903175	0.048145801	NA	Down	ENSG00000132383	RPA1
ENST00000426641	1206	-3.049903175	0.048145801	NA	Down	ENSG00000233719	GOT2P3
ENST00000472841	361	-3.049903175	0.048145801	NA	Down	ENSG00000124225	PMEPA1
ENST00000426195	243	1.535528295	0.048228022	0.607106835	Ups	ENSG00000236684	AL645728.3
ENST00000296142	1321	-3.046749767	0.048254937	NA	Down	ENSG00000163825	RTP3
ENST00000296511	1762	-3.046749767	0.048254937	NA	Down	ENSG00000164111	ANXA5
ENST00000426514	1493	-3.046749767	0.048254937	NA	Down	ENSG00000063176	SPHK2
ENST00000466106	1419	-3.046749767	0.048254937	NA	Down	ENSG00000141127	PRPSAP2
ENST00000469000	587	-3.046749767	0.048254937	NA	Down	ENSG00000164091	WDR82
ENST00000491613	959	-3.046749767	0.048254937	NA	Down	ENSG00000143774	GUK1
ENST00000527429	1612	-3.046749767	0.048254937	NA	Down	ENSG00000137726	FXYD6
ENST00000586866	4120	-3.046749767	0.048254937	NA	Down	ENSG00000180448	HMHA1
ENST00000543610	1967	-1.275748471	0.048255907	0.607106835	Down	ENSG00000196118	C16orf93
ENST00000409986	2005	-2.497167699	0.048299947	NA	Down	ENSG00000115318	LOXL3
ENST00000444664	1305	-1.281952586	0.048329191	0.607106835	Down	ENSG00000160691	SHC1

ENST00000507205	1215	-2.80128046	0.048380751	NA	Down	ENSG00000185324	CDK10
ENST00000493417	2833	-1.672385357	0.048393897	0.607106835	Down	ENSG00000148358	GPR107
ENST00000598662	2833	-1.672385357	0.048393897	0.607106835	Down		
ENST00000308666	6238	-3.019104168	0.048449559	NA	Down	ENSG00000173208	ABCD2
ENST00000426717	5927	-3.019104168	0.048449559	NA	Down	ENSG00000150672	DLG2
ENST00000434254	824	1.480993676	0.048462709	0.607106835	Ups	ENSG00000228681	AC008072.1
ENST00000396568	1962	-1.705035371	0.048532675	0.607106835	Down	ENSG00000181284	TMEM102
ENST00000435459	2849	3.039653136	0.048555252	NA	Ups	ENSG00000206561	COLQ
ENST00000465552	878	3.039653136	0.048555252	NA	Ups	ENSG00000168036	CTNNB1
ENST00000484975	3059	-2.161716526	0.048563282	0.607106835	Down	ENSG00000101473	ACOT8
ENST00000521942	1991	1.823845029	0.04856939	0.607106835	Ups	ENSG00000145736	GTF2H2
ENST00000512494	411	2.02784449	0.048587366	0.607106835	Ups	ENSG00000138735	PDE5A
ENST00000232496	1691	3.005620759	0.048596054	NA	Ups	ENSG00000114383	TUSC2
ENST00000522883	601	3.005620759	0.048596054	NA	Ups	ENSG00000102225	CDK16
ENST00000575920	1691	3.005620759	0.048596054	NA	Ups		
ENST00000603554	601	3.005620759	0.048596054	NA	Ups		
ENST00000514183	1439	-1.501033327	0.04860682	0.607106835	Down	ENSG00000204628	GNB2L1
ENST00000461609	1545	-3.048362109	0.048608949	NA	Down	ENSG00000144891	AGTR1
ENST00000510089	7608	-3.048362109	0.048608949	NA	Down	ENSG00000164180	TMEM161B
ENST00000529919	4780	-3.048362109	0.048608949	NA	Down	ENSG00000198561	CTNND1
ENST00000393644	945	1.825429803	0.048625311	0.607106835	Ups	ENSG00000213358	AC092933.4
ENST00000594901	2118	3.009617364	0.048683903	NA	Ups	ENSG00000269343	ZNF587B
ENST00000376377	1350	3.044432688	0.048708904	NA	Ups	ENSG00000137331	IER3
ENST00000439476	4532	3.033326781	0.048727507	NA	Ups	ENSG00000176697	BDNF
ENST00000468389	1393	1.78288229	0.048750689	0.607106835	Ups	ENSG00000123243	ITIH5
ENST00000417948	697	3.001014369	0.048768179	NA	Ups	ENSG00000185022	MAFF
ENST00000341976	4020	1.505209477	0.048792356	0.607106835	Ups	ENSG00000187792	ZNF70
ENST00000355653	2734	-1.054787712	0.048794616	0.607106835	Down	ENSG00000108828	VAT1
ENST00000463083	1487	-2.405427975	0.048841164	0.607149173	Down	ENSG00000186577	C6orf1
ENST00000254835	3964	2.99980697	0.048863693	NA	Ups	ENSG00000132498	ANKRD20A3
ENST00000469580	174	2.99980697	0.048863693	NA	Ups	ENSG00000239576	COX6CP14
ENST00000470252	2541	2.694792749	0.048909328	NA	Ups	ENSG00000107819	SFXN3
ENST00000406606	4691	-3.018102192	0.048915047	NA	Down	ENSG00000160293	VAV2
ENST00000444645	1180	-3.018102192	0.048915047	NA	Down		
ENST00000462612	1791	3.03722931	0.048944252	NA	Ups	ENSG00000179152	TCAIM
ENST00000258412	2371	-3.043837908	0.048971677	NA	Down	ENSG00000135926	TMBIM1
ENST00000326965	3051	3.041023456	0.048982927	NA	Ups	ENSG00000164691	TAGAP

ENST00000473129	1518	2.170036632	0.049005741	NA	Ups	ENSG00000163607	GTPBP8
ENST00000509910	4333	3.007887194	0.049043347	NA	Ups	ENSG00000145782	ATG12
ENST00000564546	2104	-1.225621796	0.04905172	0.609228421	Down	ENSG00000149925	ALDOA
ENST00000558657	1852	2.15555043	0.049059993	NA	Ups	ENSG00000259388	CTD-2647E9.1
ENST00000475031	6966	2.059391059	0.049107768	0.60938669	Ups	ENSG00000105792	C7orf63
ENST00000432131	682	3.012539966	0.04912543	NA	Ups	ENSG00000183696	UPP1
ENST00000576768	6342	-2.260332358	0.049207223	0.609591794	Down	ENSG00000167291	TBC1D16
ENST00000451754	1977	1.367590611	0.049210935	0.609591794	Ups	ENSG00000223724	RAD17P2
ENST00000591041	812	-3.008517222	0.049281243	NA	Down	ENSG00000126247	CAPNS1
ENST00000317872	2552	-1.363778098	0.049358557	0.61049873	Down	ENSG00000178972	AC097658.1
ENST00000469255	1803	2.316247935	0.049391444	0.61049873	Ups	ENSG00000159208	C1orf51
ENST00000486832	3095	1.637502584	0.049414302	0.61049873	Ups	ENSG00000114098	ARMC8
ENST00000568881	587	2.99259749	0.049442396	NA	Ups	ENSG00000140398	NEIL1
ENST00000551712	1573	1.71994269	0.049515835	0.610906539	Ups	ENSG00000177425	PAWR
ENST00000498153	1114	2.513055691	0.04953317	NA	Ups	ENSG00000198223	CSF2RA
ENST00000510501	1779	1.245468471	0.049534136	0.610906539	Ups	ENSG00000250031	RP11-114M5.1
ENST00000514051	1058	3.025426157	0.049584878	NA	Ups	ENSG00000164024	METAP1
ENST00000429849	957	1.721007421	0.049586386	0.611015434	Ups	ENSG00000214700	C12orf71
ENST00000484964	1870	-3.033312318	0.049679832	NA	Down		
ENST00000367630	7028	1.918267157	0.049705484	0.611412213	Ups	ENSG00000009844	VTA1
ENST00000553572	3055	-3.00310869	0.049710374	NA	Down	ENSG00000258705	AE000660.4
ENST00000461239	617	2.99870281	0.049789788	NA	Ups	ENSG00000093144	ECHDC1
ENST00000431952	1922	2.596021621	0.049808301	NA	Ups	ENSG00000067066	SP100
ENST00000379953	1197	-2.167678318	0.049825628	NA	Down	ENSG00000112799	LY86
ENST00000304141	3229	-3.033036352	0.049830774	NA	Down	ENSG00000168497	SDPR
ENST00000471443	524	3.005093793	0.049837851	NA	Ups	ENSG00000115419	GLS
ENST00000595236	2062	1.165141487	0.049912612	0.612679947	Ups		
ENST00000571896	3498	2.990869607	0.049934492	NA	Ups	ENSG00000072864	NDE1
