# Supplementary material for: The Role of lncRNA AF117829.1 in the Immunological Pathogenesis of Severe Aplastic Anaemia
Source: Oxid Med Cell Longev. 2021 Mar 16;2021:5587921. doi: 10.1155/2021/5587921 (PMC10435305; doi:10.1155/2021/5587921)
Supplement: Supplementary 2 — Supplementary file S2: the RNA-seq results of 194 differentially expressed lncRNAs (107 upregulated and 87 downregulated lncRNAs) in CD8+ T lymphocytes from SAA patients, including lncRNA ID, its length, log2 fold change, p values, adjusted p values, and expression (up- or downregulation). [file 5587921.f2.pdf]

gene_id	length	log2FoldChange	pvalue	padj	Regulation
TCONS_00021883	2258	-4.544933697	8.01E-05	0.049014877	Down
TCONS_00126226	1430	3.994025687	9.46E-05	0.049014877	Ups
TCONS_00198388	1178	-4.277291549	0.000226651	0.068809639	Down
TCONS_00412525	1684	-1.583067364	0.000312063	0.068809639	Down
TCONS_00188170	1102	-3.154583519	0.000332093	0.068809639	Down
TCONS_00275509	4407	-3.097139996	0.000529255	0.091384678	Down
TCONS_00188744	622	-3.599914979	0.000684857	0.101358791	Down
TCONS_00392672	901	2.645547149	0.001032363	0.133690988	Ups
TCONS_00361902	315	2.550948369	0.00135681	0.13641179	Ups
TCONS_00061949	818	3.454001724	0.001391142	0.13641179	Ups
TCONS_00429205	357	3.771383361	0.001450927	NA	Ups
TCONS_00240278	2238	-2.69718737	0.001490526	0.13641179	Down
TCONS_00144773	475	2.456741682	0.001623994	0.13641179	Ups
TCONS_00152812	416	2.407138912	0.001902615	0.13641179	Ups
TCONS_00392720	1150	-3.695883485	0.001980886	NA	Down
TCONS_00132046	953	2.788091152	0.001994012	0.13641179	Ups
TCONS_00361009	682	3.345664778	0.002102524	0.13641179	Ups
TCONS_00406127	4999	-1.964439253	0.002106746	0.13641179	Down
TCONS_00329529	1288	2.348718703	0.002510129	0.145105183	Ups
TCONS_00000163	2293	-2.291707455	0.002713137	0.145105183	Down
TCONS_00048874	346	2.354090443	0.002880057	0.145105183	Ups
TCONS_00412269	2809	-3.551528912	0.00300475	NA	Down
TCONS_00340307	1268	-2.32220906	0.003037826	0.145105183	Down
TCONS_00217935	1036	-2.236976854	0.003085508	0.145105183	Down
TCONS_00371279	719	3.011413211	0.003196844	0.145105183	Ups
TCONS_00412061	603	2.936019818	0.003344169	0.145105183	Ups
TCONS_00021654	692	-3.110098921	0.00336151	0.145105183	Down
TCONS_00160151	280	3.19222427	0.003625763	NA	Ups
TCONS_00413280	348	-2.44543571	0.004134377	0.165012731	Down
TCONS_00065869	3355	-2.075872686	0.004150576	0.165012731	Down
TCONS_00100383	3381	1.832458376	0.004332313	0.165012731	Ups
TCONS_00140949	932	3.393887601	0.00442698	NA	Ups
TCONS_00412324	737	-3.422683102	0.004528912	NA	Down
TCONS_00275523	1277	2.315071738	0.004561694	0.165012731	Ups
TCONS_00363479	547	2.09289182	0.004619082	0.165012731	Ups
TCONS_00043996	2311	1.569867118	0.005258523	0.181199369	Ups
TCONS_00119811	3333	-3.361050167	0.005308934	NA	Down
TCONS_00091220	904	-3.384340517	0.005366918	NA	Down
TCONS_00341215	3023	-2.853018286	0.005421989	0.181199369	Down
TCONS_00380395	841	1.992613667	0.005654943	0.183078779	Ups
TCONS_00160152	630	1.413009841	0.005912997	0.184555494	Ups
TCONS_00251924	3282	-1.943007379	0.006056841	0.184555494	Down
TCONS_00266360	204	2.561444672	0.006279389	0.185869923	Ups
TCONS_00164640	219	2.317104523	0.006600224	0.189939782	Ups
TCONS_00164085	2461	-3.305159629	0.006712293	NA	Down

TCONS_00027557	6472	-2.385164851	0.007193915	0.201429619	Down
TCONS_00085376	1688	-3.25495951	0.007443367	NA	Down
TCONS_00166191	951	1.790329693	0.007480049	0.203929754	Ups
TCONS_00392674	1516	-3.250845026	0.007864331	NA	Down
TCONS_00068091	206	2.979267934	0.008241184	0.218919648	Ups
TCONS_00041562	907	-2.964849215	0.008601482	NA	Down
TCONS_00165562	278	3.180351801	0.008751429	NA	Ups
TCONS_00100984	3372	1.834263795	0.008907623	0.22792115	Ups
TCONS_00118863	861	3.157285943	0.008946731	NA	Ups
TCONS_00316829	591	1.816403679	0.009020046	0.22792115	Ups
TCONS_00016718	1443	2.176967478	0.0092584	0.228373871	Ups
TCONS_00156556	632	3.128475263	0.009727975	NA	Ups
TCONS_00119782	2059	-2.014779626	0.010253502	0.234661952	Down
TCONS_00220845	662	-2.502641148	0.010511013	0.234661952	Down
TCONS_00355496	1172	2.155309949	0.010648894	0.234661952	Ups
TCONS_00178467	1545	-1.783385837	0.010721185	0.234661952	Down
TCONS_00022526	438	3.079279031	0.011025143	NA	Ups
TCONS_00398810	4968	-1.756651022	0.011045102	0.234661952	Down
TCONS_00012207	2219	2.11046804	0.011437913	0.234661952	Ups
TCONS_00068776	254	2.140469567	0.011523263	0.234661952	Ups
TCONS_00063412	1401	-1.548839656	0.011955096	0.234661952	Down
TCONS_00316728	732	-2.458762734	0.012047328	0.234661952	Down
TCONS_00116853	571	1.793907681	0.012050215	0.234661952	Ups
TCONS_00160787	1816	1.812425003	0.012530053	0.234661952	Ups
TCONS_00164101	7546	-1.410857609	0.012771037	0.234661952	Down
TCONS_00406128	4584	-1.497674768	0.012856859	0.234661952	Down
TCONS_00177371	286	1.395539716	0.012881546	0.234661952	Ups
TCONS_00142384	1839	3.027913513	0.013556812	NA	Ups
TCONS_00026350	1892	-2.880905713	0.013753444	NA	Down
TCONS_00291677	471	1.425518128	0.013820503	0.234661952	Ups
TCONS_00103649	1583	1.724937342	0.013824151	0.234661952	Ups
TCONS_00188172	1682	-1.717210203	0.013898159	0.234661952	Down
TCONS_00164086	2097	-1.720681098	0.013995001	0.234661952	Down
TCONS_00021720	1981	-2.267223584	0.014125636	0.234661952	Down
TCONS_00352189	1825	-1.542129869	0.01417098	0.234661952	Down
TCONS_00334414	444	1.980317238	0.014269984	0.234661952	Ups
TCONS_00272470	2232	2.944979123	0.015676696	NA	Ups
TCONS_00007789	877	-2.971544915	0.015952591	NA	Down
TCONS_00225946	2050	-1.592892998	0.016595979	0.268647414	Down
TCONS_00133376	1986	2.238187706	0.017204114	NA	Ups
TCONS_00227969	6200	-1.589709331	0.018335238	0.290373417	Down
TCONS_00047737	1649	-2.510159485	0.018468613	NA	Down
TCONS_00085019	1731	-1.44459076	0.018695948	0.290373417	Down
TCONS_00244164	616	2.658770079	0.018745679	NA	Ups
TCONS_00149085	2099	1.529670155	0.018778976	0.290373417	Ups
TCONS_00264550	762	2.307646551	0.019303069	NA	Ups

TCONS_00169172	483	2.619600571	0.019413852	NA	Ups
TCONS_00360056	1171	1.882920677	0.019978026	0.296230201	Ups
TCONS_00251921	3395	-1.99021061	0.020134722	0.296230201	Down
TCONS_00060779	340	2.844047127	0.02014596	NA	Ups
TCONS_00006148	580	2.852324923	0.020167076	NA	Ups
TCONS_00048955	654	-2.282317575	0.020242344	0.296230201	Down
TCONS_00008255	743	2.808587332	0.020307281	0.296230201	Ups
TCONS_00043638	4747	-1.330251042	0.020587427	0.296230201	Down
TCONS_00248885	1823	1.385104601	0.021161374	0.300317576	Ups
TCONS_00389665	704	-2.322807292	0.021265694	NA	Down
TCONS_00014085	596	1.353269494	0.021460553	0.300447739	Ups
TCONS_00140754	394	1.281671789	0.021909093	0.302637608	Ups
TCONS_00411193	2730	-2.722336919	0.022057418	NA	Down
TCONS_00116933	4740	-2.834106814	0.022065099	NA	Down
TCONS_00048913	272	2.274575157	0.02224135	NA	Ups
TCONS_00361903	263	1.859249624	0.022704335	0.305802269	Ups
TCONS_00375781	467	1.664998533	0.022981921	0.305802269	Ups
TCONS_00117395	1295	1.471734771	0.023023723	0.305802269	Ups
TCONS_00025171	271	2.022983731	0.023791017	0.309195863	Ups
TCONS_00119810	13740	-1.702549453	0.023876128	0.309195863	Down
TCONS_00109722	419	-2.174542546	0.025721022	0.322960875	Down
TCONS_00260310	30303	1.365911534	0.025929434	0.322960875	Ups
TCONS_00166059	1446	1.236656906	0.02598988	0.322960875	Ups
TCONS_00374303	558	2.745376877	0.026024273	NA	Ups
TCONS_00157090	1458	-2.203264537	0.026186017	0.322960875	Down
TCONS_00094490	1477	-2.612019246	0.026579092	NA	Down
TCONS_00219346	3179	2.435917798	0.026843017	NA	Ups
TCONS_00320552	2182	-2.537657825	0.027346289	NA	Down
TCONS_00272636	515	2.067761911	0.027586475	0.336230443	Ups
TCONS_00218053	1947	-2.393419867	0.027681617	NA	Down
TCONS_00063221	7114	-2.723702267	0.027963328	NA	Down
TCONS_00142383	1682	-2.349239348	0.028071493	NA	Down
TCONS_00335440	2825	-2.725997771	0.028093844	NA	Down
TCONS_00142840	721	1.186722523	0.028157117	0.339195041	Ups
TCONS_00390160	361	2.701301705	0.028623486	NA	Ups
TCONS_00120135	486	-2.545048687	0.02865866	NA	Down
TCONS_00257954	2125	-1.975660182	0.028862036	0.339771298	Down
TCONS_00264987	1073	-1.383435747	0.029024951	0.339771298	Down
TCONS_00048910	208	1.390223441	0.029624585	0.339771298	Ups
TCONS_00421124	216	1.275123166	0.029820222	0.339771298	Ups
TCONS_00221988	1383	1.449351282	0.030051729	0.339771298	Ups
TCONS_00091431	966	2.675445239	0.030311143	NA	Ups
TCONS_00352129	2075	1.863056914	0.030337675	0.339771298	Ups
TCONS_00195286	483	1.477334245	0.030500705	0.339771298	Ups
TCONS_00111256	306	-1.531256488	0.030923051	0.340811501	Down
TCONS_00391439	635	2.051226513	0.031791018	0.344109903	Ups

TCONS_00117176	2460	1.692315277	0.031886811	0.344109903	Ups
TCONS_00374352	545	1.703538032	0.032635594	0.344109903	Ups
TCONS_00145489	1493	-1.853209988	0.032672479	0.344109903	Down
TCONS_00222746	616	1.598461746	0.032883089	0.344109903	Ups
TCONS_00413294	3073	1.364648032	0.033530713	0.345941758	Ups
TCONS_00309937	4602	1.644755726	0.033725982	0.345941758	Ups
TCONS_00212108	3516	1.872032543	0.034150826	0.346865257	Ups
TCONS_00342259	1784	2.614951423	0.035149448	NA	Ups
TCONS_00185433	393	-2.333914736	0.035579204	0.355635886	Down
TCONS_00059573	1294	-1.238419105	0.036056282	0.355635886	Down
TCONS_00227821	370	1.84123985	0.036056641	0.355635886	Ups
TCONS_00013819	4822	-1.742316519	0.036387455	0.355635886	Down
TCONS_00377689	528	2.257970561	0.036864134	NA	Ups
TCONS_00100602	563	-2.594113114	0.036864421	NA	Down
TCONS_00379951	1572	-2.469351421	0.037362493	NA	Down
TCONS_00003405	790	-2.579697697	0.038176609	NA	Down
TCONS_00256567	475	-2.323281688	0.038228426	NA	Down
TCONS_00148635	258	1.199396236	0.038288727	0.370332095	Ups
TCONS_00002554	428	2.152388395	0.03831879	NA	Ups
TCONS_00113608	3609	-1.240661751	0.039173895	0.370332095	Down
TCONS_00221322	1123	1.555673338	0.039307017	0.370332095	Ups
TCONS_00129120	546	-2.302745782	0.039315069	NA	Down
TCONS_00134845	546	-2.302745782	0.039315069	NA	Down
TCONS_00115214	4227	1.57750451	0.039320975	0.370332095	Ups
TCONS_00277536	439	-2.56501062	0.039540087	NA	Down
TCONS_00374191	396	1.998890059	0.039934306	NA	Ups
TCONS_00390986	1266	-1.505985611	0.041171157	0.381012635	Down
TCONS_00087041	781	1.374250771	0.041914367	0.381012635	Ups
TCONS_00316623	328	1.176377124	0.042035029	0.381012635	Ups
TCONS_00308930	227	1.227034656	0.042517003	0.381012635	Ups
TCONS_00111255	328	-1.519175008	0.042808198	0.381012635	Down
TCONS_00113045	715	-1.943847201	0.043281875	0.381012635	Down
TCONS_00412968	675	-1.273022952	0.043318146	0.381012635	Down
TCONS_00396952	251	2.507814282	0.043507907	NA	Ups
TCONS_00375628	735	1.519542043	0.043657449	0.381012635	Ups
TCONS_00088719	1386	-1.386778949	0.043859749	0.381012635	Down
TCONS_00311947	564	1.44298802	0.044132738	0.381012635	Ups
TCONS_00336036	1014	2.371306579	0.044511399	NA	Ups
TCONS_00116850	549	2.214763842	0.045282864	NA	Ups
TCONS_00213344	510	2.484136761	0.045416812	NA	Ups
TCONS_00407557	367	2.485855075	0.04565448	NA	Ups
TCONS_00199446	315	-2.358089958	0.045747342	NA	Down
TCONS_00084828	552	-2.238324019	0.045945464	NA	Down
TCONS_00410927	1916	-1.656362357	0.04608521	0.382363071	Down
TCONS_00046821	283	1.192122162	0.046191768	0.382363071	Ups
TCONS_00014855	773	-2.483243217	0.046254056	NA	Down

TCONS_00410436	232	1.804162923	0.046502396	0.382363071	Ups
TCONS_00333608	597	1.533610863	0.046763069	0.382363071	Ups
TCONS_00415606	329	1.207225023	0.046814361	0.382363071	Ups
TCONS_00335573	1370	-1.477745809	0.046872693	0.382363071	Down
TCONS_00138996	504	-1.301016658	0.047535181	0.384737875	Down
TCONS_00216064	808	1.240899757	0.048155885	0.385312343	Ups
TCONS_00148636	235	1.009589527	0.048350004	0.385312343	Ups
TCONS_00135720	296	2.241404187	0.048778059	NA	Ups
TCONS_00152811	589	1.470320166	0.049085149	0.388184846	Ups
TCONS_00146784	789	-2.448673674	0.049448242	NA	Down
TCONS_00215116	806	2.438887785	0.049472921	NA	Ups
